# Implementation of a complex intervention to improve interprofessional collaboration in long-term care: results of the mixed-methods process evaluation within the *interprof* ACT trial

**DOI:** 10.1186/s12913-026-14270-2

**Published:** 2026-03-07

**Authors:** Frederike Lüth, Clarissa Elisabeth Weber, Christian Kortkamp, Linda Steyer, Christiane Annette Müller, Britta Tetzlaff, Ana Lucia Mazur, Anja Kühn, Martin Scherer, Sascha Köpke, Tim Friede, Eva Hummers, Indre Maurer, Katrin Balzer

**Affiliations:** 1 Institute for Social Medicine and Epidemiology, Nursing Research Group, University of Lübeck, Lübeck, Germany; 2 Department of Business and Society, School of Business and Management, Queen Mary University of London, London, UK; 3 Independent Researcher, Göttingen, Germany; 4 Department of General Practice, University Medical Center Göttingen, Göttingen, Germany; 5 Department of General Practice and Primary Care, University Medical Center Hamburg-Eppendorf, Hamburg, Germany; 6 Institute of Nursing Science, University of Cologne, Medical Faculty & University Hospital Cologne, Köln, Germany; 7 Department of Medical Statistics, University Medical Center Göttingen, Göttingen, Germany; 8 Chair of Organization and Corporate Development, Faculty of Business and Economics, Georg-August-University Göttingen, Göttingen, Germany

**Keywords:** Nursing homes, Interprofessional collaboration, Complex intervention, Process evaluation, Mixed-methods study, Cluster-randomized controlled trial

## Abstract

**Background:**

The cluster randomized controlled trial *interprof* ACT evaluated the effects of a complex intervention designed to improve collaboration between general practitioners (GPs) and registered nurses (RNs) in nursing homes (NHs). The intervention includes six components (“Name badges”, “Mandatory availability rules”, “Designated contact persons”, “Standardized GPs’ home visits”, “Pro re nata medication”, “Shared goal setting”). The findings showed a nonsignificant reduction in hospital admissions in the intervention group (IG) compared to the control group (CG) within twelve months. The aims of this process evaluation were to describe (1) the dose, reach and fidelity of implementation (“implementation performance”), (2) the effects on the quality of RN-GP collaboration, and (3) potential moderating factors.

**Methods:**

Process evaluation with a mixed-methods triangulation design involving all clusters (17 NHs per IG and CG) and 323 nursing home residents (NHRs) (*n* = 166 IG, *n* = 157 CG): We collected quantitative and qualitative data from multiple perspectives (e.g., RNs, GPs, NHRs) at several measurement points. We quantitatively compared groups by means of medians, interquartile ranges, proportions (all outcome domains) or Mann‒Whitney U tests (implementation performance) and analyzed qualitative data inductively via content analysis. The key findings were triangulated narratively and via a joint display.

**Results:**

Compared to those in the CG, we noted relevant improvements in the implementation of “Name badges”, “Mandatory availability rules”, “Designated contact persons” and “Pro re nata medication” in ≥50% of the IG clusters, of which the group difference for “Mandatory availability rules” reached statistical significance. The implementation performance of IG clusters was moderated by resource-related and other organizational attributes of NHs and GP offices and attributes of involved professionals, especially their attitudes and awareness. Implementation of the components induced greater standardization of care processes together with positive changes in interprofessional communication and coordination among GPs and RNs.

**Conclusions:**

Implementation of the *interprof* ACT components varied between components and NHs but showed potential for improving RN-GP collaboration. The standardization of shared care procedures emerged as a key mediator for improvement. For larger and more sustainable implementation we recommend a stronger focus on locally available resources and communication of potential benefits for all involved parties.

**Trial registration:**

ClinicalTrials.gov, NCT03426475; registered 07 February 2018, https://www.clinicaltrials.gov/study/NCT03426475?lead=NCT03426475%26;rank=1.

**Supplementary Information:**

The online version contains supplementary material available at 10.1186/s12913-026-14270-2.

## Background

Nursing home residents (NHRs) have a high risk of hospital admission. On average, one NHR experiences approximately 0.5 emergency department visits and 1.2 hospital admissions per year [1] Up to 27% of these hospital admissions seem to be avoidable [2], if there is improved needs-based care. This care requires effective interprofessional collaboration between registered nurses (RNs) and general practitioners (GPs). Therefore, improving this collaboration seems to be an important mechanism for preventing avoidable hospitalization [3–5].

**Fig. 1:**
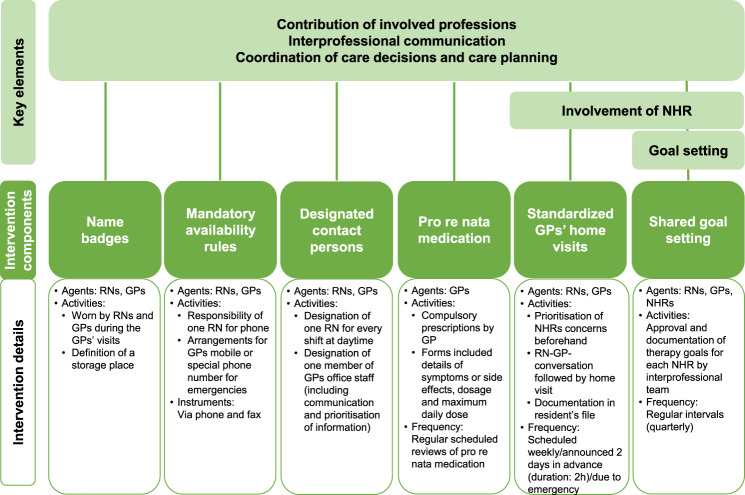
Overview of the *interprof* ACT intervention components and the addressed key elements in interprofessional collaboration. Notes: *use of a standardized *interprof* ACT sheet for the intervention component was possible. The key elements of interprofessional collaboration are based on Tsakitzidis et al. [10] (with slight adaptations to the terminology). Details of the intervention components are reported elsewhere [6, 8]. Abbreviations: *GP*, general practitioner; *NHR*, nursing home resident; *RN*, registered nurse

In the *interprof* ACT study (ClinicalTrials.gov: NCT03426475), we evaluated the effectiveness of a newly developed complex intervention to improve the interprofessional collaboration between RNs and GPs in German nursing homes (NHs) [6–8]. Building upon a previous qualitative study [9], the intervention includes six components that can be tailored to local needs (Fig. 1). Components address one or several key elements of interprofessional collaboration in primary care for NHRs [10] (Fig. 1).

To evaluate the effectiveness of the *interprof* ACT intervention package, we conducted a cluster randomized controlled trial (cRCT): participating NHs were randomly assigned to implement the intervention package or to care for NHRs as usual. The implementation was facilitated by preplanned implementation strategies: (1) the designation of *interprof* ACT agents (“*interprof* ACT-Verantwortliche”, IPAVs) in each NH, (2) two training sessions for IPAVs, (3) in-house kick-off meetings with representatives of all parties involved (e.g., NHRs, nursing home directors (NHDs)) to discuss and tailor the intervention package, and (4) regular IPAV supervision by the study team (Table 1, Additional file 2: Chapter 2.2.2).

**Table 1:** Description of implementation strategies

Designation of interprof ACT agents	First training	In-house kick-off meeting	Second training	Regular supervision
**Timepoint:** Shortly after randomization **Aim**: Designation of *interprof* ACT agent and substitute **Stakeholders:** NHDs, RNs **Content:** •Information about the *interprof* ACT study and intervention package •Initiation, coordination and monitoring of local activities for the implementation of the components **Methods:** Designation by NHDs **Duration:** not applicable	**Timepoint:** Shortly after designation of *interprof* ACT agents **Aim:** Information of the *interprof* ACT agents about the study, the objectives, components and procedures of the intervention package and their own roles and tasks **Stakeholders:** NHDs, IPAVs, STM **Content:** •Background of *interprof* ACT study •Information about the *interprof* ACT study and intervention package •Purpose, target populations and procedures of the kick-off meeting **Methods:** •Presentation •Discussion **Duration:** 120 min	**Timepoint:** 4–5 weeks after randomized allocation **Aim:** Agreement on the shape of the *interprof* ACT intervention components to be implemented in the NH **Stakeholders:** NHDs, NHRs, Legal guardians, Representatives of NHs’ advisory board, Representatives of relatives’ advisory board or interested relatives, GPs, medical assistants, RNs, IPAVs **Content:** •Introduction of *interprof* ACT intervention package •Discussion of *interprof* ACT intervention package **Methods:** In preparation: •All participants received three materials: a pro re nata medication form, a meeting form (for meetings to establish common goals) and a standardized fax form. •RNs of participating NH wards rated the local relevance and feasibility of the single components in advance, and these ratings (yes, no, which) were taken into account during the kick-off meeting discussion At the kick-off meeting: Discussion **Duration:** 120 min	**Timepoint:** 1–2 weeks after kick-off meeting **Aim:** First steps to pave the way for the local implementation of the *interprof* ACT intervention components as agreed-upon during the kick-off meeting **Stakeholders:** NHDs, IPAVs, STM **Content:** •Discussion of the kick-off meeting outcomes •Identification and reflection of potential local facilitators and barriers to successful implementation •Next steps for local implementation, including activities to be initiated by the *interprof* ACT agent **Methods:** Presentation •Discussion •Role play **Duration:** 120 min	**Timepoint:** After second training until end of study **Aim:** Reflection on the advancements and barriers occurring in the implementation of agreed-upon *interprof* ACT components **Stakeholders:** IPAVs, STM **Content:** •Degree of implementation (total and for every intervention component) •Barriers and facilitators of implementation **Methods:** Telephone/electronic contact Face-to-face meetings **Duration:** First three months after kick-off meeting: •Two to four telephone/electronic contacts per month •One to three face-to-face meetings Remaining study period: •One to two monthly telephone/electronic contacts •One face-to-face meeting every second month

Abbreviations: *GP,* general practitioner; *IPAV, interprof* ACT agent*; NH,* nursing home; *NHD,* nursing home director; *NHR,* nursing home resident; *RN,* registered nurse; *STM,* study team member

The primary trial outcome was the incidence proportion of hospital admissions to NHRs within twelve months. The findings revealed a nonsignificant reduction in hospital admissions and length of hospital stay in intervention group (IG) NHs compared to the control group (CG) but no changes in favor of the intervention in further outcomes [7]. To interpret the trial findings and understand how the *interprof* ACT components were implemented and influenced interprofessional collaboration, we conducted a process evaluation alongside the main trial, which is the subject of the present paper.

## Methods

### Aims

The process evaluation aimed at (1) assessing the implementation of the intervention package, (2) examining the effects of this implementation on the quality of RN-GP collaboration, and (3) exploring the role of potential moderating factors on implementation performance and included a quantitative and a qualitative strand. The quantitative strand addressed the following questions:

- Were implementation strategies used as planned?
- What were the reach, dose and fidelity of implementation of the *interprof* ACT intervention package, and which adaptations were made to individual components?
- What were the effects of the implementation on the quality of the RN-GP collaboration?

Furthermore, we qualitatively explored how working processes in the RN-GP collaboration and medical care for NHRs changed based on the implementation. Both strands explored context factors of the implementation.

### Design

We used a mixed-methods triangulation design [8, 11]. This design followed the recommendations for process evaluations [12, 13] and reporting [14] of complex interventions and implementation studies (StaRI) [15] (Additional file 1). The detailed methods are published elsewhere [8].

**Fig. 2:**
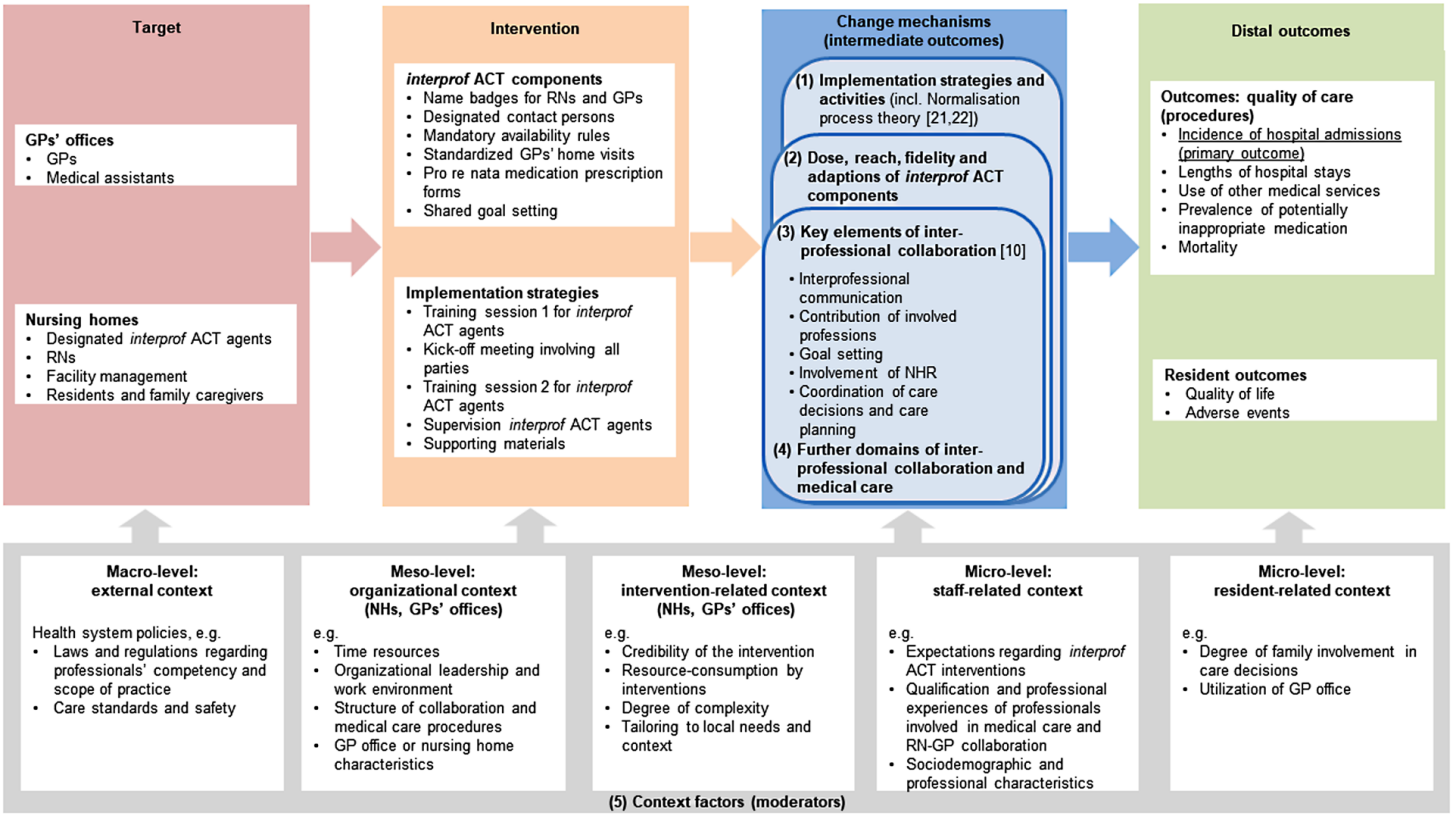
Logic model of the causal pathway of the *interprof* ACT intervention. Abbreviations: *GP*, general practitioner; *NH,* nursing home; *NHR*, nursing home resident; *RN*, registered nurse

As a theoretical foundation, we developed a logic model that illustrates the expected causal pathway of the intervention (Fig. 2). We assumed that implementation strategies would result in implementational work (intermediate outcome level 1) to adapt and tailor components, leading to a certain dose, reach and fidelity of implementation and adjustments to components (intermediate outcome level 2). Furthermore, we expected the implementation to induce changes in the key elements of RN-GP collaboration [10] (intermediate outcome level 3) and in further domains of interprofessional collaboration and medical care (intermediate outcome level 4). We also included context factors as potential implementation moderators in the model [16].

### Setting

The trial was conducted in 34 NHs in Germany at three study centers (Göttingen, Hamburg, and Lübeck). Based on the cRCT design, 17 NHs (clusters) were randomly allocated to the IG and CG.

### Target populations and data sources

We collected quantitative data repeatedly from all 34 participating NHs from all parties involved: NHDs, NHRs, RNs, GPs, IPAVs, kick-off meeting participants and moderators, and study team members. For the qualitative strand, we conducted repeated nonparticipating observations and semistructured interviews with GPs and RNs in a subsample of 10 NHs (IG: *n* = 5, CG: *n* = 5). Table 2 provides an overview of the target populations at each measuring point. The eligibility criteria and recruitment procedures are described in the protocol [8].

**Table 2:** Overview of data sources and measuring points by target groups for process evaluation (quantitative/qualitative)

Target population	Data collection	Measurement points							Target population (target sample size)
		T0a	T0b	First training (only in IG)	Kick-off meeting (only in IG)	Second training (only in IG)	T1	T2	
*Nursing/facility directors* *(both study groups)*	quantitative	x*** (Q)			x* (Q)			x*** (Q)	Nursing or facility directors of all nursing homes participating *interprof* ACT trial [6]. (total: *n* = 34, per cluster: *n* = 1)
	qualitative								Participants of nonparticipating observations during kick-off meetings (see below)
*Nursing home residents* *(both study groups)*	quantitative	x** (Q)			x* (Q)			x** (Q)	All NHRs who met the inclusion criteria for the*interprof* ACT trial [6]: •1 GP contact in the last 3 months or 2 GP contacts in the last 6 months or nursing home admission during the preceding 6 months independently of the actual number of GP contacts •18 years or older •Written informed consent for the study, either by themselves or through their legal guardian Of these, only NHRs able to answer simple questions on satisfaction with medical care were included in the process evaluation (total: *n* = 680, per cluster: *n* = 20)
	qualitative								Potential cosubject of nonparticipating observations
*General practitioners* *(both study groups)*	quantitative	x (Q)			x* (Q)			x (Q)	All GPs involved in the medical care for participating NHRs and interested in participation (total: *n* = 95, per cluster: *n* = 2–3)
	qualitative		x (I, O)				x (I, O)	x (I, O)	GPs who participated in the main trial and agreed to participate in the process evaluation (total: *n* = 16, per cluster: *n* = 2)
*Registered nurses* *(both study groups)*	quantitative	x (Q)			x* (Q)			x (Q)	All RNs working in direct nursing and medical care for the NHRs at data collection days (total: *n* = 136, per cluster: *n* = 4)
	qualitative		x (I, O)				x (I, O)	x (I, O)	RNs preselected by nursing or facility directors based on their involvement in interprofessional collaboration. RNs were included if they were available and willing to take part in the qualitative process evaluation (total: *n* = 16, per cluster: *n* = 2)
*IPAVs* *(only IG)*	quantitative			x (Q)	x* (Q)	x (Q)	x (Q)	x (Q)	All designated IPAVs or their substitutes (total: *n* = 34, per cluster: *n* = 2)
	qualitative								Participants of nonparticipating observations during kick-off meetings (see below)
*Study team members (only IG)*	quantitative			x (Q, M)	x (Q*, M)	x (Q, M)	x**** (Q, M)	x**** (Q, M)	STM involved in the supervision and training of IPAVs (total: *n* = 34–68, per cluster: *n* = 2–4)
	qualitative								Participants of nonparticipating observations during kick-off meetings (see below)
*Participants and moderators of kick-off meetings (only IG)*	quantitative				x* (Q)				Participants: all relevant stakeholders, especially NHRs and their relatives, RNs, the nursing or facility director, associated GPs, and members of the nursing home advisory board. Moderators: IPAV and 1 STM (total: *n* = 170–255, per meeting (i.e., cluster): *n* = 10–15)
	qualitative				x (O)				

*Pre-post questionnaire. **Face-to-face interview. ***Case report form of the main study. ****During supervision (questionnaire: baseline characteristics)

Abbreviations: *GP,* general practitioner; *I,* interview; *IG,* intervention group; *IPAV, interprof* ACT agent; *M,* minutes; *NHR,* nursing home resident; *O,* observation; *Q,* questionnaire; *RN,* registered nurse; *STM,* study team member; *T0a,* baseline assessment (before randomized allocation); *T0b,* shortly post randomization; *T1,* follow-up after 6 months; *T2,* follow-up after 12 months

### Outcomes and data collection methods

Table 3 displays the outcome domains and related data sources for each measurement point. We addressed the following outcome domains: (1) implementation strategies and activities; (2) implementation of *interprof* ACT components: dose, reach, fidelity, and adaptations; (3) key elements of interprofessional collaboration; (4) further domains related to interprofessional collaboration and medical care; and (5) context factors at the meso- and micro-levels.

**Table 3:** Description of outcome domains, subdomains, and dimensions at specific measurement points

Domain	Subdomain and/or dimensions	Source	Target population at specific measurement points					
			NHD	NHR	GP	RN	IPAV	STM
**(1) Implementation strategies and activities**								
Implementation of *interprof* ACT measures, barriers and facilitators		Interviews and observations			T0b T1 T2	T0b T1 T2		
Implementation strategies	Kick-off meeting, training, supervision	Self-developed	T0b	T0b	T0b	T0b	T0b T1 T2	T0b T1 T2
IPAV work	Implementation activities, e.g., discussions with the nursing team	Self-developed					T1 T2	T1 T2
Implementational work within a team	Coherence, cognitive participation, collective action, reflexive monitoring	NoMAD, self-developed				T2	T1 T2	
**(2) Implementation of interprof ACT components**								
Feasibility of *interprof* ACT measures Tailoring of *interprof* ACT measures during kick-off meeting Process changes through *interprof* ACT intervention		Interviews and observations			T0b T1 T2	T0b T1 T2		
Name badges	*Choice and adaptation, current practice*	Self-developed	T0a T2		T0a T2	T0a T2	T1 T2	T0b
Mandatory availability rules	*Choice and adaptation, attitudes, current practice, quality and satisfaction*	Koverdem, InDemA, self-developed	T2		T0a T2	T0a T2	T1 T2	T0b
Designated contact persons	*Choice and adaptation, attitudes, current practice, quality and satisfaction*	InDemA, self-developed	T0a T2		T0a T2	T0a T2	T1 T2	T0b
Standardized GPs’ home visits	*Choice and adaptation, attitudes, current practice, quality and satisfaction*	InDemA, self-developed	T0a T2	T0a T2	T0a T2	T0a T2	T1 T2	T0b
Pro re nata medication	*Choice and adaptation, quality and satisfaction*	Self-developed	T2		T0a T2	T0a T2	T1 T2	T0b
Shared goal setting	*Choice and adaptation, attitudes, current practice, quality and satisfaction*	Koverdem, InDemA, PSAT, self-developed	T0a T2		T0a T2	T0a T2	T1 T2	T0b
**(3) Key elements of interprofessional collaboration**								
Perceptions of work processes between RNs and GPs Communication and interaction between participants during kick-off meeting* Communication and interaction between RNs and GPs in everyday work		Interviews and observations			T0b T1 T2	T0b T1 T2		
Involvement of NHR	*Current practice, quality and satisfaction*	ZAP, Self-developed	T0a T2	T0a T2	T2	T2	T2	
Interprofessional communication	*Attitudes, current practice, quality and satisfaction*	Koverdem, CPAT, self-developed	T2	T0a T2	T2	T2	T1 T2	
Contribution of involved professions	*Attitudes, current practice, quality and satisfaction*	Jefferson scale, InDemA, CPAT, PSAT, self-developed			T0a T2	T0a T2	T1 T2	
Coordination of care decisions and care planning	*Attitudes, current practice*	InDemA, self-developed	T0a T2		T0a T2	T0a T2		
**(4) Further domains related to interprofessional collaboration and medical care**								
Process sequences of care, interprofessional collaboration and hospital admissions		Interviews and observations			T0b T1 T2	T0b T1 T2		
General interprofessional collaboration	*Attitudes, quality and satisfaction*	InDemA, self-developed	T0a T2		T0a T2	T0a T2	T1 T2	
General quality of (medical) care for NHR	*Quality and satisfaction*	ZAP, EUROPEP, self-developed	T0a T2	T0a T2	T2	T2	T2	
**(5) Context factors**								
Everyday work context of RNs and GPs Barriers and facilitators of process performance		Interviews			T0b T1 T2	T0b T1 T2		
Meso: organizational level	Leadership and work environment	Self-developed					T1 T2	
	Structures of collaboration and medical care procedures *Current practice, financial and staff resources*	Self-developed			T0a T2	T0a T2	T1 T2	
	GP office and NH characteristics	Koverdem, self-developed	T0a T2		T0a T2			
Micro: staff level	Sociodemographic characteristics	Self-developed			T0a T0b* T2	T0a T0b* T2	T0b	T0a T0b*
	Competence of RNs, GPs and IPAVs	Self-developed					T1 T2	
Micro: NHR level	Utilization of GP office (formal characteristics)	ZAP, self-developed		T0a T2				
	Family involvement in medical care	Koverdem		T0a T2				

*As participants and moderators of the kick-off meeting. Abbreviations: *GP,* general practitioner; *IPAV, interprof* ACT agent*; NH,* nursing home; *NHD,* nursing home director; *NHR,* nursing home resident; *RN,* registered nurse; *STM,* study team member*; T0a,* baseline assessment (before randomized allocation); *T0b,* shortly post randomization; *T1,* follow-up after 6 months; *T2,* follow-up after 12 months. Instruments: *CPAT* Collaborative Practice Assessment Tool [17]*; EUROPEP* Measures of the European Project on Patient Evaluation of General Practice Care [18]*; InDemA* Interdisciplinary Implementation of Quality Instruments for the Care of Residents with Dementia in Nursing Homes [19]; *Jefferson Scale* of Attitudes toward Physician–Nurse Collaboration [20]; *Koverdem* Survey instruments of the research project “Optimizing Cooperation between General Practitioners and Home Care Services” [21]; *NoMAD* Normalization Measure Development [22, 23]; *PSAT* Partnership Self-Assessment Tool [24]; *ZAP* Zufriedenheit in der Arztpraxis (“Satisfaction in GPs Office”) [25]

In the quantitative strand, we assessed these outcome domains by means of paper-based questionnaires and minutes containing self-developed items and/or established measurement tools [8]. The details are described in Table 3. The qualitative data collection involved nonparticipating observations of work processes and interprofessional collaboration in NHs and kick-off meetings as well as semistructured interviews with RNs and GPs. The details are described in Table 4. Additional file 2 (Chapter: 1.1) displays all items, data sources and rater perspectives used for the quantitative measurements and the themes covered by the qualitative interviews/observation guidelines, each grouped by outcome domain.

**Table 4:** Qualitative data collection

Data Source	Main focus	Procedure
Interviews	Exploring RNs’ and GPs’ perceptions and assessments of collaboration in general and in the context of the intervention	We conducted interviews with RNs and GPs at T0b, T1 and T2 based on semistructured interview guidelines. Interviews were audio-recorded and transcribed. During the data-collection process, we regularly discussed initial findings and adjusted the guidelines to emerging themes.
Nonparticipatory observations of work processes and interprofessional collaboration	Observing RN-GP interaction and communication, the use of intervention components and changes in work processes	A team of two researchers visited NHs at T0b, T1 and T2 to conduct observations in the everyday work context. We held short conversations with RNs and GPs before or after the observations to reflect on the authenticity of the situation (e.g., unusual events due to presence of researchers). Observations were guided by semistructured guidelines and did not include any active participation. Notes were taken and subsequently converted into observational reports. We discussed field experiences in team meetings with researchers that were not involved in the data collection to gain an in-depth understanding while maintaining insider-outsider roles.
Nonparticipatory observations of kick-off meetings	Observing interaction between RNs and GPs during kick-off meetings	One researcher attended the kick-off meetings of IG NHs to observe the interaction and communication between RNs and GPs when choosing and tailoring *interprof* ACT components.

Abbreviations: *GP,* general practitioner; *NH,* nursing home; *RN,* registered nurse; *T0b,* shortly post randomization; *T1,* follow-up after 6 months; *T2,* follow-up after 12 months

### Data analysis

We analyzed the quantitative and qualitative data separately, followed by iterative triangulation. We performed the statistical analyses using IBM SPSS Statistics (version 22) and Microsoft Excel. The MAXQDA (VERBI GmbH) and SAP Signavio software packages were used for qualitative data analyses.

## Quantitative strand

We analyzed the quantitative data descriptively, with one exception, as explained below. We performed analyses at the individual (baseline characteristics and outcome domain “implementation strategies and activities”) or cluster levels (all other outcome domains). For categorical variables, we determined the frequencies (proportions); for all other variables, we calculated medians, interquartile ranges (IQRs) and ranges. If psychometrically justified, we summarized items referring to the same dimension into sum scores.

To evaluate the implementation of the components and the effects on RN-GP collaboration and medical care and the effects of context factors on implementation, we aggregated single items or sum scores into summary change measures per outcome domain using a multistep approach (Table 5, Additional file 2: Chapter 1.2). For each cluster and each rater perspective (e.g., NHRs, GPs), we retrieved a global ±360° performance indicator for each component/key element of the domains “implementation of *interprof* ACT components” or “key elements of interprofessional collaboration” and “further domains related to interprofessional collaboration and medical care”. These global indicators can be interpreted as follows: The higher the indicator value of one cluster, the more consistently minimum relevant changes were measured in this cluster across all subdomains or dimensions of the domain. For each global ±360° performance indicator, we also estimated the proportion (%) of items/sum scores for which we could not estimate a summary change measure due to missing data. We named this proportion as certainty; indicator values with a certainty < 50% were marked as uncertain.

**Table 5:** Aggregation steps performed for the analysis of quantitative outcome data

Step	Aggregation procedure	Affected outcome domains	Aggregation level	
1	Descriptive determination of the pre-post changes between T0a/T1 and T2 median values for all single items or sum scores	•Implementation of *interprof* ACT components •Key elements of interprofessional collaboration •Further domains related to interprofessional collaboration and medical care •Context factors	Per cluster	Per rater perspective
2	•Aggregation of item- or sum score-specific change estimates to a ± 360° summary change measure for each subdomain or dimension (i.e., attitudes, current practice or quality and satisfaction) per outcome domain, by means of vote counting •Assessment of the certainty of these summary change estimates, based on the number of missing values per underlying items or sum scores	•Implementation of *interprof* ACT components •Key elements of interprofessional collaboration •Further domains related to interprofessional collaboration and medical care •Context factors	Per cluster	Per rater perspective
3	Aggregation of the subdomain- or dimension-specific ±360° summary change measures (except attitudes) into one global domain-specific ±360° performance indicator for each intervention component or key element of interprofessional collaboration or medical care (see details in Additional file 2: Chap. 1.1.1)	Implementation of *interprof* ACT components Key elements of interprofessional collaboration	Per cluster	Per rater perspective
4	Determination of the mean ±360° performance indicator for each intervention component	Implementation of *interprof* ACT components	Per cluster	Across rater perspectives

For the domain “implementation of *interprof* ACT components”, the ±360° global performance indicator represents a combined measure of the dose, reach and fidelity of the implementation of the respective component, summarized as “implementation performance”. We further summarized the rater-specific implementation performance values for each intervention component into a mean ±360° global performance value per cluster. Since all *interprof* ACT components addressed common practices of RN-GP collaboration, we determined the implementation performance for both the IG and CG to retrieve an exploratory indication of changes in implementation across groups during the follow-up.

To assess the implementation performance for each component and the impact of implementation on interprofessional collaboration and medical care procedures, we visually compared IGs and CGs, displaying the median and IQR values of the clusters’ ±360° global performance indicator in the domain of interest, stratified for each rater perspective (e.g., RNs, GPs). For the outcome domain “implementation of *interprof* ACT components”, we also determined the median and IQR of the clusters’ mean implementation performance across all rater perspectives for each study group and compared them using the Wilcoxon‒Mann‒Whitney U test (two-sided level of 0.05). We post hoc decided to apply this inferential test to assess the statistical reliability of the observed differences between groups. As IPAVs were only nominated in the IG, we also performed a sensitivity analysis on group comparisons excluding this rater perspective from the mean implementation performance value per cluster.

To explore potentially relevant context factors (moderators) for the implementation of components, we divided the IG clusters into two groups based on the IG-specific median of the mean implementation performance values per cluster (≥median versus <median). For each component, we descriptively compared these two groups with regard to the median and IQR values of potentially relevant cluster attributes at the organizational (meso) or staff-related (micro) levels based on our logic model. We considered only attributes with valid data available for more than 80% of the clusters. We also descriptively summarized the ratings provided by IPAVs on potential implementation facilitators and barriers during the supervision (Additional file 2: Chapter 2.4.2).

## Qualitative strand

We used qualitative content analysis [26] to identify processes and explore intervention-induced process changes in the IG. First, we used process-modeling techniques to analyze dyads of RNs and GPs to describe the process patterns of time-ordered events for each NH. Then we compared cases to identify intervention-induced changes. In addition, we coded assessments of collaboration and of the intervention, especially with regard to barriers and facilitators. Two researchers independently conducted the coding. We discussed the coding with the research team.

## Triangulation

Originally, we planned to triangulate quantitative and qualitative findings for each outcome domain by means of joint displays [8]. The initial independent analyses of each strand revealed that quantitative and qualitative findings address complementary rather than overlapping themes per outcome domain and are interrelated to one another. Therefore, we replaced the domain-specific joint displays for all outcome domains except context factors with one overarching narrative summary of key findings within our discussion, combined with a revision of relevant parts of our logic model. As the findings for the outcome domain “context factors” address adjacent, partially interrelated or overlapping moderators of implementation performance, these findings were integrated into one joint display. Depending on the direction of impact on the implementation, we grouped context factors into facilitators or barriers at the meso- or micro-level.

## Results

### Response rates and characteristics of populations

#### Quantitative strand

All 34 NHs that participated in the main trial (k = 17 per study group) provided data. Less than 60% of all GPs (IG: 65/123, CG: 64/110) involved participated in the process evaluation and received a questionnaire. The response rates of the surveys varied between 57% (RNs (CG), T2) and 100% (NHDs (CG), T0a). (Additional file 2: Chapter 2.1). Table 6 shows the baseline characteristics of the NHs, NHRs (IG: *n* = 166, CG: *n* = 157), GPs (IG: *n* = 55, CG: *n* = 47), and RNs (IG: *n* = 72, CG: *n* = 72). For most characteristics, no differences were noted between the two groups, except for the share of privately owned NHs (IG: 50% vs. CG: 77%) and GP offices (IG: 31% vs. CG: 49%) and the RNs’ median years of work experience in the participating NH (IG: 8 vs. CG: 3 years).

**Table 6:** Sample characteristics for the quantitative strand of the process evaluation (baseline measurements)

	Intervention group		Control group	
	n [%] or median [IQR]	N	n [%] or median [IQR]	N
**Nursing homes**				
Ownership, private	8 [50.0]	16	13 [76.5]	17
Number of places in long-term care	93.5 [61.6–114.8]	16	96.0 [65.0–122.5]	17
Proportion of NHRs with care levels ≥ 3*	75.3 [65.6–81.4]	14	71.8 [64.7–76.1]	17
Number of RNs (fulltime equivalent)	11.3 [9.9–18.3]	14	16.0 [9.0–22.0]	16
RN-NHR ratio	0.2 [0.1–0.2]	14	0.2 [0.2–0.2]	15
Number of all GPs per NH	12.0 [5.0–20.0]	15	10.0 [5.0–16.0]	15
Number of all GPs from participating NHRs per NH	7.0 [5.0–9.0]	16	6.0 [5.0–8.0]	17
Number of participating GPs from participating NHRs per NH	3.0 [2.0–4.0]	16	3.0 [2.0–4.0]	17
Proportion of all NHRs per NH cared for by participating GP	11.6 [7.2–20.0]	13	16.7 [6.0–26.0]	16
**Nursing home residents**				
Support of relatives for medical care, regular	33 [21.4]	154	27 [19.0]	142
Visits of NHRs at GPs office (within last 6 months), yes	35 [21.7]	161	47 [30.3]	155
Medical care by current GP in recent 1–2 years	43 [27.6]	156	33 [22.3]	148
**General Practitioners’ offices**				
Located in municipality < 100.000 inhabitants	18 [32.7]	55	19 [40.4]	47
Academic teaching practice	22 [40.7]	54	17 [36.2]	47
Medical assistants with additional qualifications, yes	29 [50.9]	55	28 [59.6]	47
**General Practitioners**				
Age, years	56.0 [50.0–60.0]	55	54.0 [47.7–60.0]	46
Sex, female	20 [36.4]	55	16 [34.8]	46
Practice ownership, yes (single)	17 [30.9]	55	23 [48.9]	47
Years of experience as GP	16.0 [10.0–25.0]	55	14.7 [8.5–24.0]	46
Number of NHs with NHRs cared for by this GP	4.0 [3.0–6.0]	54	4.0 [2.5–6.0]	47
Number of NHRs in participating NH cared for by this GP	10.0 [2.5–35.5]	53	10.0 [3.0–21.0]	47
Estimated share (%) of NHRs among total number of patients	5.0 [2.8–9.3]	53	5.0 [2.5–10.0]	45
Estimated hours per week spent on medical care for NHRs	5.2 [3.0–7.9]	54	5.0 [2.9–7.6]	47
**Registered Nurses**				
Age, years	39.0 [30.0–53.5]	69	35.0 [28.5–44.5]	65
Sex, female	56 [78.9]	71	57 [82.6]	69
First cycle nursing degree in geriatric nursing care, yes	57 [80.3]	71	57 [82.6]	69
Additional nursing qualifications, yes	32 [44.4]	72	30 [41.7]	72
Years of experience in the participating nursing home	8.0 [3.0–14.3]	70	3.0 [1.5–7.5]	64
Working hours per week	38.7 [35.5–40.0]	64	38.5 [35.0–40.0]	61
*interprof* **ACT agents (first/second training or retraining)**				
Age, years	39.0 [34.0–51.0]	43	Not applicable	
Sex, female	38 [84.4]	45	Not applicable	
First cycle nursing degree in geriatric nursing care, yes	28 [62.2]	45	Not applicable	
Additional nursing qualifications, yes	35 [79.5]	44	Not applicable	
Years of experience in the participating nursing home	5.0 [1.1–10.0]	44	Not applicable	
Working hours per week	39.0 [38.5–40.0]	43	Not applicable	

*According to the German social long-term care insurance, levels 0 to 5, with higher levels representing greater care needs. Abbreviations: *GP,* general practitioner; *IQR, interquartile range; NH,* nursing home; *NHR,* nursing home resident; *RN,* registered nurse

## Qualitative strand

The qualitative strand was conducted in a subsample of five IG and five CG NHs. The characteristics of these NHs were similar to those of the whole sample, apart from the fact that only one IG NH but all CG NHs were privately owned (Additional file 2: Chapter 2.1). In total, 2490 minutes of observation, 48 interviews with GPs and 49 interviews with RNs were conducted, lasting an average of 34 minutes.

### Implementation strategies and activities

#### Quantitative strand

The implementation strategies were used as planned, with some minor deviations regarding duration, content and methodic-didactic concepts of the IPAV training sessions. In all IG clusters, a kick-off meeting with a median number of 8 (IQR 5–10) participants was conducted. A median of 2 (minimum–maximum: 1–7) RNs, 2 (0–4) GPs, 2 (0–4) NHRs, 2 (0–5) relatives of NHRs and 1 (1–4) NHD(s) participated in each kick-off meeting. The IPAVs reported multiple activities they had executed to facilitate the implementation. The most frequently reported activities involved clarification of technical issues (18/19, varying numerators due to missing values), organization of materials (16/18), discussions with the nursing team (15/18), and accompanying care processes or GPs’ home visits in the NH (15/18). In our surveys using modified items of the Normalization Measure Development (NoMAD) [22, 23], IPAVs and RNs rated the translation of the *interprof* ACT components into routine practice. The most frequently agreed-upon items from IPAVs’ view are “I will continue to support the implementation” (T2, 21/24), “NHDs adhere to the agreements made at the kick-off meeting” (T1, 15/19), and “RNs will continue to support the implementation” (T2, 19/24), while the least frequently agreed-upon items are “The *interprof* ACT intervention package and its implementation are regularly addressed in team meetings” (T2, 6/24) and “GPs adhere to the agreements made at the kick-off meeting” (T1, 8/19, and T2, 8/24). Compared to the IPAVs, the RNs answered the NoMAD-based items less positively (Additional file 2: Chapter 2.2).

## Qualitative strand

The qualitative data revealed that GPs and RNs had reservations regarding the intervention package or did not use intervention components due to a lack of information and time resources. RNs and GPs described additional activities that had to be initiated (e.g., involvement of colleagues from the quality management unit or IT), which further required time resources.

### Implementation of interprof ACT components

#### Quantitative strand

At kick-off meetings, each cluster decided to implement a median number of four *interprof* ACT components (Min–Max: 2–6). The components “Mandatory availability rules” (16 clusters), “Name badges” (14 clusters) and “Standardized GPs’ home visits” (14 clusters) were most frequently agreed to be implemented, while “Pro re nata medication” and “Shared goal setting” were chosen by only 10 clusters (Table 7, Additional file 2: Chapter 2.3.1). During the follow-up, these decisions were sometimes revised. Eventually, IPAVs reported implementation work in almost all clusters (16 or 17 clusters per component) for all components except for “Pro re nata medication” (13 clusters) and “Shared goal setting” (11 clusters) (Table 7).

**Table 7:** Quantitative and qualitative findings on implementation performance

Decision at the kick-off meeting (IG)	Implementation work reported by IPAVs’ (IG) during follow-up*	Quantitative findings: ±360° global performance indicator (mean values per cluster across rater perspectives)			Qualitative findings
		Parameter	IG (n = 17)	CG (n = 17)	
**Name badges**					
•Decision for implementation: *n* = 14 NHs •Decision for no implementation: *n* = 3 NHs •Adaptations: Revision of NH-specific fax sheet (incl. information about priority), obtaining a reading confirmation, obtaining a special/mobile number) •Preexisting implementation: *n* = 15 NHs	Implementation work in *n* = 16 NHs	Median [IQR]	90.0 [−10.5–223.4]	0 [−69.4–71.3]	Cases of insufficient implementation mainly resulted from GPs forgetting to bring and wear the name badge or the name badge falling off when being used Benefit of the measure was not always clear to RNs and GPs Extreme case: GP not being aware of component (had forgotten of existence)
		Mann‒Whitney-U Test	*U* = 188.0 [Z = 1.51, *p* = 0.132]		
**Mandatory availability rules**					
•Decision for implementation: *n* = 16 NHs •Decision for no implementation: *n* = 1 NH •Adaptations: GP as contact person in GPs’ office, more than one medical assistant as contact person, use of in-house bell system •Preexisting implementation: *n* = 9 NHs	Implementation work in *n* = 17 NHs	Median [IQR]	91.8 [−43.5–188.3]	35.3 [−51.6–72.0]	Different fax numbers within NH not communicated to GPs Extreme case: a newly hired RN was not informed about component
		Mann‒Whitney-U Test	*U* = 218.0 [Z = 2.53, *p* = 0.011]		
**Designated contact persons**					
•Decision for implementation: *n* = 11 NHs •Decision for no implementation: *n* = 6 NHs •Adaptations: GP as contact person in GPs’ office, more than one medical assistant as contact person, use of in-house bell system Preexisting implementation: *n* = 14 NHs	Implementation work in *n* = 17 NHs	Median [IQR]	60.0 [−30.0–60.0]	0 [−135.0–135.0]	GPs were not always informed about individual phone numbers within NH Contact person at NH was not always available when needed Extreme case: Contact person (RN) forgot about task
		Mann‒Whitney-U Test	*U* = 169.0 [Z = 0.85, *p* = 0.395]		
**Standardized GPs’ home visits**					
•Decision for implementation: *n* = 14 NHs •Decision for no implementation: *n* = 3 NHs •Adaptations: Development of a home visit folder/checklist, use of a calendar •Preexisting implementation: *n* = 12 NHs	Implementation work in *n* = 17 NHs	Median [IQR]	8.0 [−30.4–101.3]	2.3 [−57.4–71.4]	Not all RNs in the NH were informed about visit folder GPs partly forgot about registration procedure Use of visit folder was partially replaced by “scribbled notes” in the everyday hustle GPs sometimes forgot to announce their visits or were not aware of announcement requirement Unavailability of contact person hampered standardized visits Ad hoc visits made standardized visits somewhat obsolete Extreme case: GP did not see benefit of component
		Mann‒Whitney-U Test	*U* = 165.5 [Z = 0.72, *p* = 0.469]		
**Pro re nata medication**					
•Decision for implementation: *n* = 10 NHs •Decision for no implementation: *n* = 7 NHs •Adaptations: Review of pro re nata medication once per quartal, addition of the name of substance and maximum dose, feedback in case of frequent or less use •Preexisting implementation: *n* = 13 NHs	Implementation work in *n* = 13 NHs	Median [IQR]	144.0 [44.1–156.6]	72.0 [−18.0–120.0]	Not all involved RNs informed about component GPs forgot review
		Mann‒Whitney-U Test	*U* = 179.0 [Z = 1.19, *p* = 0.234]		
**Shared goal setting**					
Decision for implementation: *n* = 10 NHs Decision for no implementation: *n* = 7 NHs Adaptations: Meeting frequency (3x per year, every 6 months), transfer of standardized form into existing documentation system, exemplary implementation Preexisting implementation: *n* = 7 NHs	Implementation work in *n* = 11 NHs	Median [IQR]	36.0 [−7.0–90.0]	60.0 [12.0–162.0]	RN seem to be too little informed Responsibility for initiation unclear Extreme case: Goal setting did not take place despite agreement
		Mann‒Whitney-U Test	*U* = 112.0 [Z = −1.12, *p* = 0.263]		

Abbreviations: *CG,* control group; *GP,* general practitioner; *IG,* intervention group; *IPAV, interprof* ACT agent*; IQR,* interquartile range; *NH,* nursing home; *RN,* registered nurse. ^*^Some NHs decided to implement the intervention component in the aftermath of the kick-off meeting. As a result, the number of NHs in this column may differ slightly from that displayed for the outcome “decision for implementation” in the column “decision at the kick-off meeting (IG)”

**Fig. 3:**
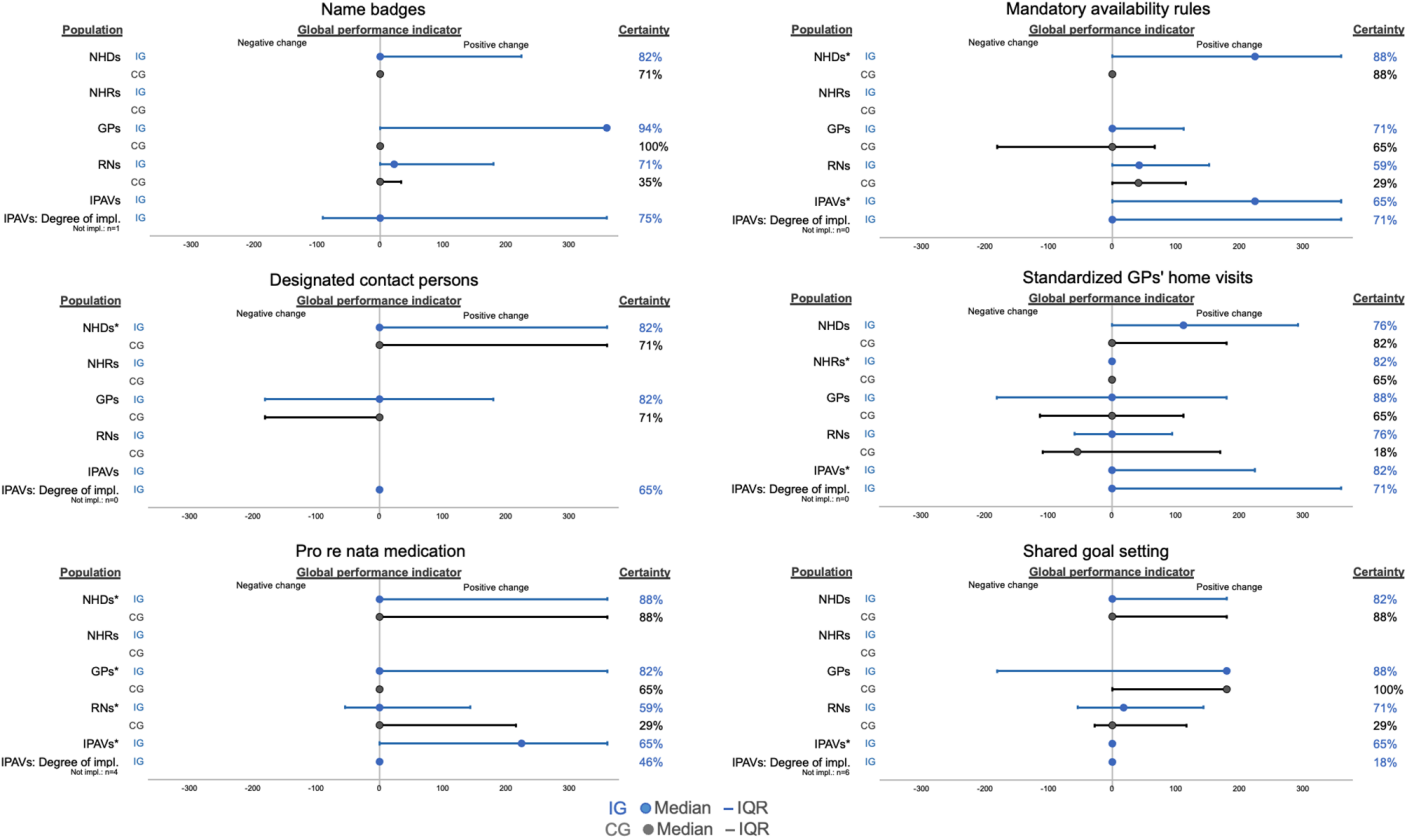
±360° global performance indicators for implementation performance at the cluster level stratified by rater perspective. Certainty = in cases where the ±360° global performance indicator contained some uncertainties due to nondisplayable changes (n.D.) because of missing data for more than 50% of items, the ±360° global performance indicator was highlighted as “uncertain”. Thus, the “certainty” represents the proportion of certain degrees of change from different perspectives. Abbreviations: *CG,* control group; *GP,* general practitioner; *IG,* intervention group; *IPAV, interprof* ACT agent*; IQR,* interquartile range; *NHD,* nursing home director; *NHR,* nursing home resident; *RN,* registered nurse

Group comparisons based on the cross-rater mean ±360° global implementation performance per cluster revealed greater values in the IG than in the CG for the components “Name badges”, “Mandatory availability rules”, “Designated contact persons” and “Pro re nata medication”, with differences ranging from +56.5° (“Mandatory availability rules”) to +90.0° (“Name badges”) (Table 7). For the component “Standardized GPs’ home visits,” we retrieved almost equal median values in the two study groups (group difference +5.7°), while for the component “Shared goal setting”, the median in the IG was −24.0° lower than that in the CG. For all but one component (“Mandatory availability rules”), the group differences were not found to be statistically significant (Table 7). Sensitivity analysis confirmed these between-group comparison results for most of the components, except for “Pro re nata medication”, for which the performance values no longer differed between groups when the IPAV values were excluded (Additional file 2: Chapter 2.3.2). Overall, ratings in favor of the IG were most frequently retrieved from the perspectives of GPs, IPAVs and NHDs (Fig. 3). With regard to attitudes toward the components, the median of the rater-specific summary change indicator values is 0° for all components across all rater perspectives both in the IG and CG, with marked uncertainties due to missing data (Additional file 2: Chapter 2.3.3).

## Qualitative strand

The qualitative findings indicate that implementation problems and failures were mainly related to insufficient information flows among NH staff or between RNs and GPs (Table 7). In addition, GPs sometimes lacked awareness of agreed-upon changes to routine practices, and individuals in both groups were sometimes not convinced of the usefulness of the components to be implemented, especially regarding the components “Name badges” and “Standardized GPs’ home visits”. In addition, practical difficulties were noted that hampered the implementation of the components “Mandatory availability rules”, “Designated contact persons” and “Standardized GPs’ home visits”, while ambiguities about responsibilities were attributed to incomplete implementation of “Shared goal setting”.

### Context factors of implementation

#### Quantitative strand

We descriptively compared the IG clusters with a mean ±360° implementation performance value equal to or above the group median (upper group) to the IG clusters below the group median (lower group) with regard to several cluster attributes at the meso- and micro-levels. The comparisons revealed heterogeneous group differences in size and direction for most attributes across the intervention components (Table 8, Additional file 2: Chapter 2.4.1). For five factors at the meso-level, we noted higher median values in the upper group for ≥3 components, indicating facilitation of implementation: a greater number of NHRs cared for by one GP office (6 components), better nursing staff resources indicated by larger RN-NHR ratios (4 components), larger NHs (4 components), a greater proportion of GP offices involved in academic teaching (4 components), and subjectively perceived improvements in GPs’ financial resources for care for NHRs (3 components). For three context factors at the meso-level, we repeatedly (≥3 components) found higher median values in the lower group, indicating hindering effects: subjectively perceived improvements in nursing staff resources by RNs and IPAVs (4 components), preexisting formal cooperation agreements between the NH and GP offices (3 components), and higher proportions of NHRs with increased care needs (3 components). The inverse association between subjectively perceived improvements in nursing staff resources and implementation performance affected all four components for which larger RN-NHR ratios were noted in the upper group. At the micro-level, for three components, we found a lower median RNs’ length of professional experience in the higher-performance group, indicating an inverse impact on implementation.

**Table 8:** Joint display of quantitative and qualitative findings on context factors

		Name badges	Mandatory availability rules	Designated contact persons	Standardized GPs’ home visits	Pro re nata medication	Shared goal setting
**Meso (organizational) level**							
**Nursing homes**							
	**Readiness/commitment of NHDs for implementation**	↑^**Quan IPAV**^					
	**Better staff resources (higher RN-NHR ratio or subjectively perceived improvements)**	^↑Quan^	^↑Quan*^	^↑Quan^	^↑Quan*^	^↑Quan*^	^↑Quan*^
	**Larger size**	↓^**Quan**^	^↑Quan^	^↑Quan^	^↑Quan^	^↑Quan^	↓^**Quan**^
	Higher number of GPs involved in medical care for NHRs	↔^Quan^	↑^Quan^	↔^Quan^	↔^Quan^	↔^Quan^	↔^Quan^
	Preexisting formal cooperation agreement with GPs	↓^Quan^	↓^Quan^	↔^Quan^	↑^Quan^	↔^Quan^	↓^Quan^
	Higher proportion of NHRs with care levels ≥ 3	↑^Quan^	↔^Quan^	↓^Quan^	↓^Quan^	↓^Quan^	↔^Quan^
**GPs/GPs’ offices**							
	**Higher number of NHRs cared for by GPs’ office**	↑^**Quan**^	^↑Quan^	^↑Quan^	^↑Quan^	^↑Quan^	^↑Quan^
	**Involvement in academic teaching**	^↓**Quan**^	^↑Quan^	^↓**Quan**^	^↑Quan^	^↑Quan^	^↑Quan^
	Improved financial resources for care for NHRs (subjective assessment)	↔^Quan^	↑^Quan^	↔^Quan^	↑^Quan^	↑^Quan^	↔^Quan^
**Micro-level**							
**RNs**							
	Longer professional experience	↓^Quan^	↑^Quan^	↓^Quan^	↔^Quan^	↓^Quan^	↔^Quan^
**GPs**							
	**Medical competencies of GPs**	↑^**Quan IPAV**^					
**RNs and GPs**							
	Lack of perceived usefulness of components	↓^Qual^	↔^Qual^	↔^Qual^	^↓Qual^	↔^Qual^	↔^Qual^
	**Insufficient recall/lacking awareness**	^↓Qual^	^↓**Qual**^	^↓**Qual**^	^↓**Qual**^	^↓**Qual**^	^↓**Qual**^
	**Unchanged attitudes toward the component**	^↓Quan^	^↓**Quan**^	^↓**Quan**^	^↓**Quan**^	^↓**Quan**^	^↓**Quan**^
	**Insufficient time resources**	^↓Qual^					
*interprof* **ACT agents**							
	**Collaboration with NHD**	^↑Quan IPAV^					
	**Own professional competencies and experiences**	^↑Quan IPAV^					
	**Experienced recognition from GPs**	^↓Quan IPAV^					
	**Experienced recognition from NHRs and relatives**	^↓Quan IPAV^					
	**Experienced recognition from nursing team colleagues**	^↓Quan IPAV^					
	**Insufficient time resource**	^↓Quan IPAV^					
**Everyday work procedures**							
	Practical/technical issues	↓^Qual^	↔^Qual^	↓^Qual^	↓^Qual^	↔^Qual^	↔^Qual^
	Information gaps between RNs and GPs	↔^Qual^	↓^Qual^	↓^Qual^	↔^Qual^	↔^Qual^	↓^Qual^

↑Facilitating effects; ↓ Hindering effects; ↔ No indication of association in quantitative data or not noted as facilitator or barrier in qualitative data. Bold letters indicate context confirmed for ≥4 intervention components or component-independent factors (shown as one arrow per row). *Positive association between a higher RN-NHR ratio and implementation performance (facilitating effects) coupled with an inverse association with subjectively perceived improvements in staff resources (hindering effects). Abbreviations: *GP,* general practitioner; *IPAV, interprof* ACT agent; *NHD,* nursing home director; *NHR,* nursing home resident; *Qual,* qualitative strand; *Quan,* quantitative strand: comparison of upper versus lower group of clusters in the intervention group, based on the median split for the mean ±360° performance indicator value per cluster (Additional file 2: Chapter 2.4.1); *Quan IPAV,* quantitative strand: factors with facilitating effects confirmed by most IPAV ratings and factors with hindering effects confirmed by fewer IPAV ratings (Additional file 2: Chapter 2.4.2); *RN,* registered nurse

During supervision, the IPAVs rated several context factors at the meso- and micro-levels with regard to the perceived impact on implementation progress (Additional file 2: Chapter 2.4.2). During the supervision, especially in the second half of the intervention phase, most of the IPAVs judged factors related to their own competencies and the competencies of the GPs and the support by the NHDs as helpful for implementation. In contrast, at some of the later supervisions, less than 50% experienced supporting recognition of their competencies by nursing staff members, GPs or NHRs and their families or rated the number of RNs in the NH and available time resources as helpful for implementation (Table 8).

## Qualitative strand

In addition to the implementation problems described above (Table 7), a major theme in the qualitative data analysis was the general problem of insufficient time available in everyday work for both RNs and GPs, which either caused or amplified the implementation problems, for example, forgetting about component use. For GPs, the more time-consuming components were also considered from a cost‒benefit perspective. 

### Interprofessional collaboration and medical care

#### Quantitative strand

**Fig. 4:**
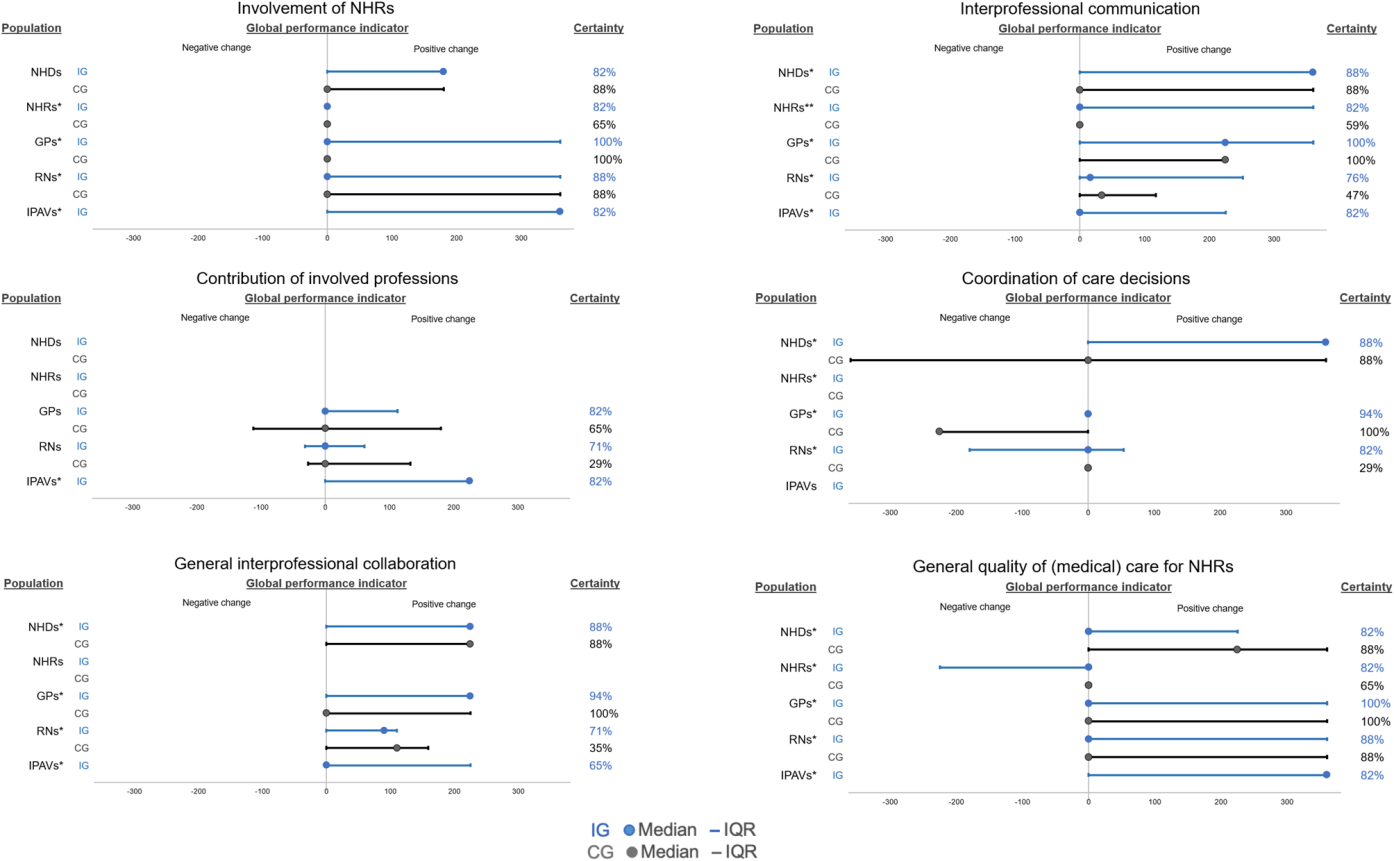
±360° global performance indicators for interprofessional collaboration at the cluster level stratified by rater perspective. Certainty = in cases where the ±360° global performance indicator contained some uncertainties due to nondisplayable changes (n.D.) because of missing data for more than 50% of items, the ±360° global performance indicator was highlighted as “uncertain”. Thus, the “certainty” represents the proportion of certain degrees of change from different perspectives. Abbreviations: *CG,* control group; *GP,* general practitioner; *IG,* intervention group; *IPAV, interprof* ACT agent*; IQR,* interquartile range; *NHD,* nursing home director; *NHR,* nursing home resident; *RN,* registered nurse

For three out of the six outcome subdomains of interprofessional collaboration and medical care, the median and IQR values of the rater-specific ±360° global performance indicator are more consistently located in the range of positive changes (1°–360°) and are larger in the IG than in the CG: involvement of NHRs, interprofessional communication, and coordination of care decisions (Fig. 4). For the other subdomains, the respective rater-specific values of the IG were mostly equal to or even lower than those of the CG. Across all subdomains, positive changes in the IG were mostly reported by GPs, IPAVs and NHDs. From the NHRs’ perspective, positive changes in favor of the IG were only noted for “interprofessional collaboration”. For attitudes toward interprofessional collaboration, the median rater-specific change indicator values were consistently 0° for all subdomains in both groups, except for NHDs’ attitudes toward “interprofessional collaboration” and GPs’ attitudes toward “coordination of care decisions” in the CG (Additional file 2: Chapter 2.5).

## Qualitative strand

Despite the implementation hurdles outlined above, the qualitative data showed that the *interprof* ACT intervention package impacted interprofessional work processes. In particular, the following four aspects surfaced: 1) fostering process standardization, 2) preventing process loops, 3) establishing uniform communication processes and process accountability, and 4) establishing information systems.

1. The introduction of *interprof* ACT helped foster process standardization; i.e., it resulted in agreements that provided a framework for both RNs and GPs regarding what their interaction should look like and resulted in both actors’ efforts to adhere to this frame. For example, while GPs sometimes left the NH after a consultation without providing information to the RN; having information exchange included as a fixed step in the standardized visit prevented such action.
2. The standardization of processes also implied a simplification and streamlining of processes. In particular, it prevented time-consuming loops that resulted from a lack of coordination. Before the introduction of the standardized visit, for example, there were no arrangements for how to initiate the visit. The “Standardized GPs’ home visits” included an agreement that the GP announced a visit in advance. This agreement allowed RNs and GPs to meet at a fixed time instead of either leading to a GP searching for an RN when arriving.
3. In particular, the “Mandatory availability rules” and the “Pro re nata medication” created more uniform communication processes that prevented inefficient communication attempts, such as when RNs could not reach a GP via fax or had contacted them with minor issues that could have been solved by administering pro re nata medication. Both of these measures allowed for quicker and more efficient communication and coordination between RNs and GPs that strained their time resources to a lesser extent. At the same time, such measures provided clear accountability distribution, the latter being particularly valued by the RNs who were not able to provide medication to an NHR without the GPs’ explicit agreement before the implementation of pro re nata medication.
4. The standardization of processes, especially those of the GP visit, further allowed for up-to-date information systems that both actors perceived as helpful. Using standardized folders and pro re nata medication forms, for example, helped to keep both RNs and GPs up-to-date with regard to an NHR’s current situation and thus facilitated communication and coordination even when one actor was not available for communication.

Taken together, the implementation of components led to more efficient processes that both RNs and GPs valued because it left both more flexible in their overall work execution. Measures that allowed for standardization of processes (“Mandatory availability rules”, “Standardized GPs’ home visits”, and “Pro re nata medication”) were particularly helpful.

## Discussion

### Summary of findings (triangulation)

Our process evaluation revealed that the majority of the IG clusters undertook targeted efforts to implement four or more of the six *interprof* ACT components. However, the quantitative and qualitative strands show that the implementation of components varied both within and between IG clusters. For four components, namely, “Name badges”, “Mandatory availability rules”, “Designated contact persons” and “Pro re nata medication”, we found indications of minimum consistent implementation improvements measured from several perspectives in the IG clusters compared to pre-post changes in the CG clusters. For the component “Mandatory availability rules”, these improvements appear to be more reliable than for the other components, as it was the only difference that reached statistical significance. For the components “Standardized GPs’ home visits” and “Shared goal setting”, our quantitative data demonstrated no relevant improvements. Regardless of the quantitatively detected differences between the *interprof* ACT components, qualitative findings revealed repeated cases of insufficient implementation for all components.

Our quantitative and qualitative data suggest that the clusters’ implementation performance in IGs was moderated by an interplay of several partially component-specific context factors at the meso- and micro-levels. The most consistent facilitators at the meso-level are a greater number of NHRs cared for by the same GP office, better nursing staff resources, a larger NH size, and GPs’ involvement in academic teaching. RNs, IPAVs and GPs lacking time and awareness of agreed-upon procedures were mentioned as barriers to the implementation of all or most components. Notably, the facilitating impact of a higher RN-NHR ratio on the implementation of the four components was coupled with an inverse (i.e., hindering) impact of subjectively perceived improvements in nursing staff resources on implementation performance. This contradictory finding may be an indication of impeding influences induced by changes in team cohesion. Additionally, we cannot rule out that RNs in the better-staffed, high-performance clusters viewed changes in available resources more critically. From the IPAVs’ perspective, the commitment of NHDs and their own and the GPs’ professional competencies were supportive. At the micro-level, we noted that RNs’ and GPs’ attitudes toward the *interprof* ACT components remained unchanged during follow-up for all components, and the implementation of five components was disrupted by persistent information gaps among and between RNs and GPs. Furthermore, IPAVs perceived a lack of recognition of their implementation work by team colleagues, GPs, and NHRs or their relatives as hindering.

Despite the heterogeneous implementation improvements observed for each component, both quantitative and qualitative data indicate positive changes in interprofessional collaboration induced by the implementation of the components. While the quantitative findings suggest small but notable improvements in interprofessional communication, coordination of care decisions and the involvement of NHRs, the qualitative findings illustrate mechanisms that may have mediated these effects. The standardization of structures and procedures in shared medical care for NHRs appears to enable improvements in interprofessional collaboration, as it helps to streamline communication procedures, clarifies professionals’ accountability for process steps and care decisions and improves access to and the reliability of information.

**Fig. 5:**
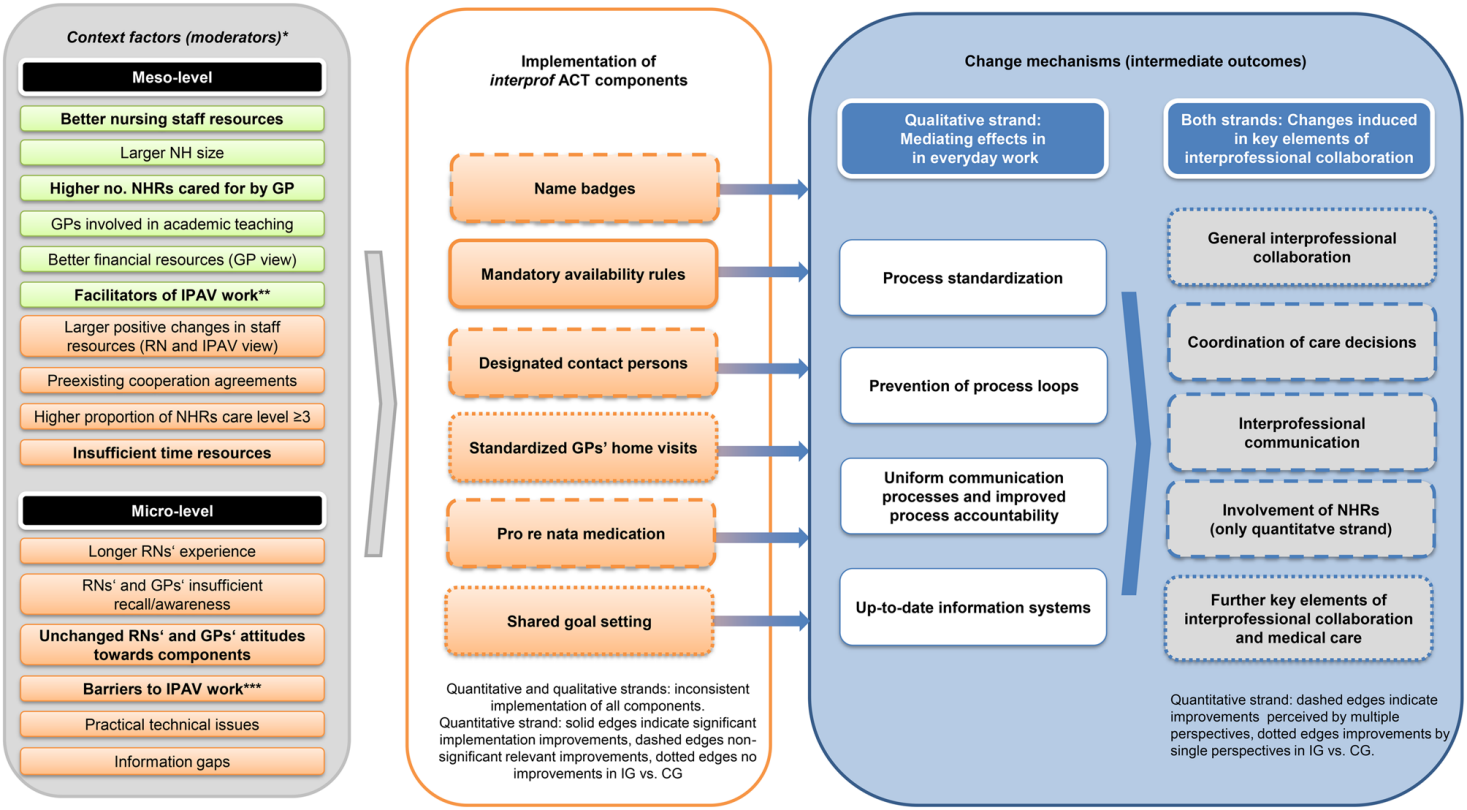
Revised logic model on the implementation of *interprof* ACT components, related moderators and change mechanisms. *Consistent facilitating (green colored boxes) or hindering (orange colored boxes) effects observed on ≥ 3 or more components. Context factors with consistent or overarching impact on all the *interprof* ACT components are highlighted by bold letters. The factors are supported by either quantitative or qualitative data (Table 8). **IPAV rating during supervision encounters: meso-level: commitment of NHDs to implementation of *interprof* ACT components; micro-level: collaboration with NHDs, IPAVs’ own and GPs’ professional competencies. ***IPAV rating during supervision encounters: insufficient recognition of IPAVs’ implementation work by nursing team colleagues, GPs, NHRs and their relatives and insufficient time resources. Abbreviations: *CG,* control group; *GP,* general practitioner; *IG,* intervention group; *IPAV, interprof* ACT agent*; NH,* nursing home; *NHR,* nursing home resident; *RN,* registered nurse

To account for the insights into the moderators of component implementation and change mechanisms, we revised parts of the a priori logic model (Fig. 5). While not all context factors appear equally relevant to each component, we recommend considering all of them when implementing the *interprof* ACT intervention package or similar measures. The observed standardization of communication and care procedures induced by the implementation of the components extends our prior understanding of change mechanisms and is now integrated as a key mediator of successive effects on interprofessional collaboration and medical care for NHRs. Although we assume that the individual *interprof* ACT components vary in their impact on this standardization and related changes, our data are too limited to discriminate between individual effects, as this change mechanism is an unexpected finding.

### Implications for the effectiveness of the interprof ACT intervention package

The results of our process evaluation corroborate and help explain the findings of the main trial, which indicated a small but nonsignificant improvement in the 12-month cumulative incidence of hospital admissions among NHRs (IG: 44.6%, CG: 46.7%, adjusted odds ratio 0.82, 95% confidence interval 0.55; 1.22) [7]. The implementation of *interprof* ACT components not only differed between components and clusters but was also likely too limited in reach and dose to cause relevant improvements in RN-GP collaboration and shared care procedures. As both the findings of the process evaluation and the main trial indicate that positive changes were induced, they underscore the potential of the *interprof* ACT intervention package to positively influence the health and well-being of NHRs and prevent potentially avoidable hospital admissions. Improvements in interprofessional collaboration are still assumed to be an important mechanism for reducing inappropriate emergency department admissions [3] and hospitalizations in NHRs [27, 28]. Therefore, the NHR-relevant benefits of the *interprof* ACT components should be tested in further trials, which should be complemented by revised strategies to facilitate more effective implementation.

### Implications for future implementation strategies

In the current trial, we used a combination of theory-based, widely recommended strategies to promote the learning and implementation of the *interprof* ACT components by all involved parties, e.g., by joint kick-off meetings, local tailoring and nomination of IPAVs acting as change agents [8]. Our data show that these strategies were mostly implemented as planned, and the IPAVs reported high levels of commitment and undertook a range of activities to boost implementation. However, our strategies appeared to be insufficient for addressing prevalent barriers.

The relevant context factors observed in our study affect the meso- and micro-levels and are confirmed by existing implementation research on integrative [16] or interprofessional care [29]. While our logic model did not hierarchize context factors, a more recent evidence-based model of context factors assigns “human factors” (micro-level) a central role for successful implementation of complex interventions to change care practices in the NH setting [30]. These human factors include attitudes toward and confidence in the intervention of the people involved and team cohesion and are regarded as key to successful implementation. In our trial, the attitudes of the main actors (RNs, GPs) toward the intervention components remained unaffected by our implementation strategies.

Human factors are moderated by other attributes of the implementation context, e.g., the characteristics of the organization and the intervention [30, 31]. Our study revealed several organizational attributes relevant to implementation, including NH size, number of NHRs cared for by one GP office, nursing staff and time resources or GPs’ involvement. While not all of them are amenable to change, at least in the short term, they should be subject to policy efforts to promote a care infrastructure that facilitates interprofessional collaboration and improves the quality of care in the NH setting. Furthermore, interprofessional education should be systematically included in health professionals’ pre- and postregistration education programs to promote their long-term competencies in interprofessional collaboration [32, 33].

While GP choice is up to the discretion of individual NHRs in Germany, our findings indicate improved facilitation of interprofessional care when a greater number of NHRs in one NH are cared for by the same GP, i.e., when NHs collaborate with fewer GPs. We therefore recommend that NHs and GPs invest in formal partnerships [34]. In our study, the existence of such agreements was related to a lower implementation performance for three *interprof* ACT components. This seemingly contradictory finding should be interpreted with caution, as the ±360° performance indicator reflects changes achieved over time, and clusters with preexisting formal agreements may have had less potential for further improvements.

Our data only allow indirect assessment of the perceived complexity and resource demands of individual components. We considered the heterogeneous implementation performance and the partially component-specific impact of context factors as indications that the attributes of the components also matter. The lack of implementation improvements noted for the components “Standardized GPs’ home visits” and “Shared goal setting” suggest that these components were perceived as more challenging to implement than others. Furthermore, our qualitative data revealed that GPs’ commitment to the implementation of components was subject to cost‒benefit considerations, i.e. accounting required time resources against potential benefits.

Future implementation strategies should therefore target the interaction between “human factors” [30], the resource demands required for component implementation, and further environmental attributes. Our previously used strategies should be embedded in a more team-oriented, participatory and bottom-up approach that could use elements of promising “Living Lab” methods [35]. As part of this approach, representatives of all involved parties achieve consensus regarding the components to be implemented, considering the benefits to expect, for example, due to standardization, against the efforts required for implementation. Established criteria such as the F.A.C.E. instrument [36] and the findings of our process evaluation may facilitate this priority setting. The effectiveness of these strategies should be evaluated using hybrid trial designs that allow for inferences on intervention and implementation outcomes [37].

### Limitations

One major challenge of the quantitative strand was the aggregation of the multiple measures used to assess the single outcome domains across different evaluation perspectives. We developed the new ±360° global performance indicator, which reflects the consistency of the within-group changes noted across the single items or subscores and the rater perspectives per outcome domain but not the size of changes. In the IG, the IPAVs’ perspective was included in the performance indicator values, while this perspective did not exist in the CG. To control for this impact on the group comparisons, we performed a sensitivity analysis excluding the IPAV data. The results largely confirmed the findings from the main analysis. Furthermore, while the aggregation of item or subscore values was cross-checked for a subset of items with no indication of systematic errors, the validity and reliability of the performance indicators were not tested before application in our analyses. However, given the theoretical plausibility of our findings and the consistency of the qualitative and quantitative results, we assume that the performance indicator is sufficiently valid.

Additionally, response rates below 80% for some target groups (NHRs, GPs and RNs) and missing values (especially for attitudes) limit the reliability and generalizability of the quantitative findings. Due to the descriptive nature of our process evaluation, no imputation of missing values or adjustments for cluster-related effects were conducted.

A limitation of the qualitative strand is that we were only able to use data from 5 NHs, since 5 of the 10 NHs belonged to the CG.

## Conclusions

Our findings indicate that the *interprof* ACT intervention package has the potential to improve interprofessional collaboration between RNs and GPs in NHs. The standardization of interprofessional communication and care coordination appears to be an important change mechanism for achieving these effects. However, the implementation of components varied markedly between components and clusters and was likely too small to induce clinically relevant changes in the main trial outcomes. The attitudes of RNs and GPs were merely affected by the implementation strategies used in our trial, but a number of context factors at the organizational level were identified to potentially facilitate the implementation.

For practice development and future research, we suggest a revision of the implementation strategies in future evaluations of the *interprof* ACT components or similar interventions. In a participatory and team-oriented approach, implementation should start with criteria-based prioritization of the interventions to be implemented. The results of our process evaluation provide valuable information for defining and applying these criteria.

## Electronic Supplementary Material

Below is the link to the electronic supplementary material.

Supplementary Material 1

Supplementary Material 2

## Data Availability

The data are available upon reasonable request to the publication committee of the *interprof* ACT project.

## Abbreviations

CG: Control group
cRCT: Cluster-randomized controlled trial
GP: General practitioner
IG: Intervention group
IPAV: *Interprof* ACT agent
IQR: Interquartile range
Max: Maximum
Min: Minimum
NH: Nursing home
NHD: Nursing home director
NHR: Nursing home resident
NoMAD: Normalization measure development
RN: Registered nurse
StaRI: Standards for Reporting Implementation Studies
T0a: Baseline before randomized allocation
T0b: Shortly post randomization
T1: Follow-up after 6 months
T2: Follow-up after 12 months
