# Supplementary material for: Implementation of a complex intervention to improve interprofessional collaboration in long-term care: results of the mixed-methods process evaluation within the interprof ACT trial
Source: BMC Health Serv Res. 2026 Mar 7;26:484. doi: 10.1186/s12913-026-14270-2 (PMC13063920; doi:10.1186/s12913-026-14270-2)
Supplement: Supplementary file 1 — Supplementary Material 1 [file 12913_2026_14270_MOESM1_ESM.docx]

**Additional file 1:** **Standards for Reporting Implementation Studies: the StaRI checklist [1]**

**Table 1.1** Reporting guideline: Standards for Reporting Implementation Studied (StaRI)

| **Checklist item** | | **Reported on page #** | **Implementation Strategy** | **Reported on page #** | **Intervention** |
| --- | --- | --- | --- | --- | --- |
|  | |  | “Implementation strategy” refers to how the intervention was implemented |  | “Intervention” refers to the healthcare or public health intervention that is being implemented. |
| **Title and abstract** | | | | | |
| Title | **1** | 1 | Identification as an implementation study, and description of the methodology in the title and/or keywords | | |
| Abstract | **2** | 2-4 | Identification as an implementation study, including a description of the implementation strategy to be tested, the evidence-based intervention being implemented, and defining the key implementation and health outcomes. | | |
| **Introduction** | | | | | |
| Introduction | **3** | 5-6 | Description of the problem, challenge or deficiency in healthcare or public health that the intervention being implemented aims to address. | | |
| Rationale | **4** | 5-6 | The scientific background and rationale for the implementation strategy (including any underpinning theory/framework/model, how it is expected to achieve its effects and any pilot work). | 5-6 | The scientific background and rationale for the intervention being implemented (including evidence about its effectiveness and how it is expected to achieve its effects). |
| Aims and objectives | **5** | 6 | The aims of the study, differentiating between implementation objectives and any intervention objectives. | | |
| **Methods: description** | | | | | |
| Design | **6** | 7 | The design and key features of the evaluation, (cross referencing to any appropriate methodology reporting standards) and any changes to study protocol, with reasons | | |
| Context | **7** | 7 | The context in which the intervention was implemented. (Consider social, economic, policy, healthcare, organisational barriers and facilitators that might influence implementation elsewhere). | | |
| Targeted ‘sites’ | **8** | 7-8 | The characteristics of the targeted ‘site(s)’ (e.g locations/personnel/resources etc.) for implementation and any eligibility criteria. | 7-8 | The population targeted by the intervention and any eligibility criteria. |
| Description | **9** | 5-6 | A description of the implementation strategy | 5-6 | A description of the intervention |
| Sub-groups | **10** | n.a. | Any sub-groups recruited for additional research tasks, and/or nested studies are described | | |
| **Methods: evaluation** | | | | | |
| Outcomes | **11** | 8 | Defined pre-specified primary and other outcome(s) of the implementation strategy, and how they were assessed. Document any pre-determined targets | 5-6 | Defined pre-specified primary and other outcome(s) of the intervention (if assessed), and how they were assessed. Document any pre-determined targets |
| Process evaluation | **12** | 5-6,8 | Process evaluation objectives and outcomes related to the mechanism by which the strategy is expected to work | | |
| Economic evaluation | **13** | n.a. | Methods for resource use, costs, economic outcomes and analysis for the implementation strategy | n.a. | Methods for resource use, costs, economic outcomes and analysis for the intervention |
| Sample size | **14** | 7-8 | Rationale for sample sizes (including sample size calculations, budgetary constraints, practical considerations, data saturation, as appropriate) | | |
| Analysis | **15** | 8-11 | Methods of analysis (with reasons for that choice) | | |
| Sub-group analyses | **16** | n.a. | Any a priori sub-group analyses (e.g. between different sites in a multicentre study, different clinical or demographic populations), and sub-groups recruited to specific nested research tasks | | |
| **Results** | | | | | |
| Characteristics | **17** | 12 | Proportion recruited and characteristics of the recipient population for the implementation strategy | 12 | Proportion recruited and characteristics (if appropriate) of the recipient population for the intervention |
| Outcomes | **18** | 13-15, 17-19 | Primary and other outcome(s) of the implementation strategy | n.a. | Primary and other outcome(s) of the Intervention (if assessed) |
| Process outcomes | **19** | 13-15, 17-19 | Process data related to the implementation strategy mapped to the mechanism by which the strategy is expected to work | | |
| Economic evaluation | **20** | n.a. | Resource use, costs, economic outcomes and analysis for the implementation strategy | n.a. | Resource use, costs, economic outcomes and analysis for the intervention |
| Sub-group analyses | **21** | n.a. | Representativeness and outcomes of subgroups including those recruited to specific research tasks | | |
| Fidelity/ adaptation | **22** | 12-13 (Additional file 2: Chapter 2.2) | Fidelity to implementation strategy as planned and adaptation to suit context and preferences | 14-15  (Additional file 2: Chapter 2.3) | Fidelity to delivering the core components of intervention (where measured) |
| Contextual changes | **23** | 16-17  (Additional file 2: Chapter 2.4) | Contextual changes (if any) which may have affected outcomes | | |
| Harms | **24** | n.a. | All important harms or unintended effects in each group | | |
| **Discussion** | | | | | |
| Structured discussion | **25** | 20-26 | Summary of findings, strengths and limitations, comparisons with other studies, conclusions and implications | | |
| Implications | **26** | 23-25 | Discussion of policy, practice and/or research implications of the implementation strategy (specifically including scalability) | 22-23 | Discussion of policy, practice and/or research implications of the intervention (specifically including sustainability) |
| **General** | | | | | |
| Statements | **27** | 27-30 | Include statement(s) on regulatory approvals (including, as appropriate, ethical approval, confidential use of routine data, governance approval), trial/study registration (availability of protocol), funding and conflicts of interest | | |

Abbreviations: *n.a.,* not applicable.

**Reference**

1. Pinnock H, Barwick M, Carpenter CR, Eldridge S, Grandes G, Griffiths CJ, et al. Standards for Reporting Implementation Studies (StaRI) Statement. BMJ. 2017;356:i6795.
