# Supplementary material for: Implementation of a complex intervention to improve interprofessional collaboration in long-term care: results of the mixed-methods process evaluation within the interprof ACT trial
Source: BMC Health Serv Res. 2026 Mar 7;26:484. doi: 10.1186/s12913-026-14270-2 (PMC13063920; doi:10.1186/s12913-026-14270-2)
Supplement: Supplementary file 2 — Supplementary Material 2 [file 12913_2026_14270_MOESM2_ESM.docx]

**Additional file 2: Additional information for the publication “****Implementation of a complex intervention to improve interprofessional collaboration in long-term care: Results of the mixed-methods process evaluation within the *interprof* ACT trial”**

**Table of contents**

[1 Methods 2](#_Toc175319010)

[1.1 Outcomes and data collection methods 2](#_Toc175319011)

[1.1.1 Quantitative strand 2](#_Toc175319012)

[1.1.2 Qualitative strand 29](#_Toc175319013)

[1.2 Data analysis 30](#_Toc175319014)

[1.2.1 Detailed description of the data analysis for quantitative process evaluation 30](#_Toc175319015)

[2 Results 35](#_Toc175319016)

[2.1 Response rates and characteristics of populations 35](#_Toc175319017)

[2.1.1 Response rates 35](#_Toc175319018)

[2.1.2 Sample description: Nursing homes 36](#_Toc175319019)

[2.1.3 Sample description: Nursing home residents 39](#_Toc175319020)

[2.1.4 Sample description: General practitioners’ offices 40](#_Toc175319021)

[2.1.5 Sample description: General practitioners 41](#_Toc175319022)

[2.1.6 Sample description: Registered Nurses 43](#_Toc175319023)

[2.1.7 Sample description: *interprof* ACT agents (intervention group in quantitative process evaluation only) 45](#_Toc175319024)

[2.2 Implementation strategies and activities 46](#_Toc175319025)

[2.2.1 Use of implementation strategies 46](#_Toc175319026)

[2.2.2 Implementation activities (*interprof* ACT agents) 51](#_Toc175319027)

[2.2.3 Incorporation of the *interprof* ACT components into routine care (NoMAD questionnaire) 52](#_Toc175319028)

[2.3 Implementation of *interprof* ACT components 53](#_Toc175319029)

[2.3.1 Decisions made during the kick-off meetings 53](#_Toc175319030)

[2.3.2 Sensitivity analysis for implementation performance 57](#_Toc175319031)

[2.3.3 Changes in attitudes toward intervention components 58](#_Toc175319032)

[2.4 Context factors of implementation 59](#_Toc175319033)

[2.4.1 Relevant context factors for the six intervention components 59](#_Toc175319034)

[2.4.2 Context factors mentioned by *interprof* ACT agents during supervision 65](#_Toc175319035)

[2.5 Interprofessional collaboration and medical care 67](#_Toc175319036)

# Methods

## Outcomes and data collection methods

### Quantitative strand

**Note:** Details of the item origins and pre-test of single measurement instruments are described in the protocol of the process evaluation [1]. Originally German-language items or items that were adopted from existing scales but had to be adapted to the subject of our study have been translated into English specifically for this manuscript.

**Table 2.1** Items of questionnaires and minutes for the outcome domain “implementation strategies and activities”

| **Domain,** Subdomain and/or *dimensions* | **Source** | **Target population at specific measurement points** | | | | | | | | | | | |
| --- | --- | --- | --- | --- | --- | --- | --- | --- | --- | --- | --- | --- | --- |
| Items of questionnaires/minutes |  | **NHD** | | **NHR** | | **GP** | | **RN** | | **IPAV** | | **STM** | |
|  |  | **T0b** | **T2** | **T0b** | **T2** | **T0b** | **T2** | **T0b** | **T2** | **T1** | **T2** | **T0b** | **T1–T2** |
| **Implementation strategies** |  |  |  |  |  |  |  |  |  |  |  |  |  |
| Kick-off meeting |  |  |  |  |  |  |  |  |  |  |  |  |  |
| Date, start and end time | self-developed |  |  |  |  |  |  |  |  |  |  | M |  |
| Participants (name and role: nursing home director, RN, GP, nursing home resident, relative, other) | self-developed |  |  |  |  |  |  |  |  |  |  | M |  |
| Which intervention component(s) is/are already being implemented in the nursing home?  (name badges, mandatory availability rules, designated contact persons, standardized GPs‘ home visits, pro re nata medication, shared goal setting) | self-developed |  |  |  |  |  |  |  |  |  |  | M |  |
| For each of the six intervention components:  For each topic the speakers and comments were documented (free text) | self-developed |  |  |  |  |  |  |  |  |  |  | M |  |
| Which intervention components should be implemented in the nursing home, with adaptation if necessary?  (name badges, mandatory availability rules, designated contact persons, standardized GPs‘ home visits, pro re nata medication, shared goal setting) | self-developed |  |  |  |  |  |  |  |  |  |  | M |  |
| I am satisfied with the results of the discussion. (1=agree completely … 5=disagree completely) | self-developed | Q (Mo,P) |  | Q (P) |  | Q (P) |  | Q (P) |  | Q (Mo) |  | Q (Mo) |  |
| How confident are you that the implementation of the agreed intervention components will be successful?  (1=very confident … 6=not at all confident) | self-developed | Q (Mo,P) |  | Q (P) |  | Q (P) |  | Q (P) |  | Q (Mo) |  | Q (Mo) |  |
| First and second training and retraining |  |  |  |  |  |  |  |  |  |  |  |  |  |
| Date, start and end time | self-developed |  |  |  |  |  |  |  |  |  |  | M (1^st^, 2^nd^, re-training) | |
| Participants (number and role):  *Interprof* ACT agent and substitute, other participants (free text) | self-developed |  |  |  |  |  |  |  |  |  |  | M (1^st^, 2^nd^, re-training) | |
| How satisfied were you with the training provided? ( 1=highly satisfied … 5=highly dissatisfied) | self-developed, based on Richter et al. [2] |  |  |  |  |  |  |  |  |  |  | M (1^st^, 2^nd^, re-training) | |
| Please assess whether all the contents of the different topics were covered as planned?  (1=all topics were covered as planned … 5=no topics were covered as planned + free text to describe adaptations) | self-developed, based on Richter et al. [2] |  |  |  |  |  |  |  |  |  |  | M (1^st^, 2^nd^, re-training) | |
| Were there any deviations from the methodological-didactical concept within the individual topics?  (1=no deviations … 5=complete deviations + free text to describe adaptations) | self-developed, based on Richter et al. [2] |  |  |  |  |  |  |  |  |  |  | M (1^st^, 2^nd^, re-training) | |
| How satisfied were you with the training regarding the content?  (1=totally…4=not at all) | self-developed, based on Richter et al. [2] |  |  |  |  |  |  |  |  | Q (1^st^, 2^nd^, re-training) | |  |  |
| How confident are you to implement the tasks and roles covered in the training at your workplace? (1=very confident … 6=not at all confident) | self-developed, based on Richter et al. [2] |  |  |  |  |  |  |  |  | Q (1^st^, 2^nd^, re-training) | |  |  |
| When you take a look back and think about the trainings you received at the beginning of the study: How useful were they for you?  (1=very useful … 6=not at all useful) | self-developed, based on Richter et al. [2] |  |  |  |  |  |  |  |  |  | Q |  |  |
| Supervision |  |  |  |  |  |  |  |  |  |  |  |  |  |
| Date, start and end time | self-developed |  |  |  |  |  |  |  |  |  |  | M | |
| Participants (number and role: *interprof* ACT agent/substitute, other (free text) + reason for supervision with other participants (holiday of *interprof* ACT agent/substitute, illness, other working time, change of *interprof* ACT agent, other (free text)) | self-developed |  |  |  |  |  |  |  |  |  |  | M | |
| How satisfied are you with the current level of implementation?  (1=very dissatisfied … 10=very satisfied) | self-developed, based on Richter et al. [2] |  |  |  |  |  |  |  |  | M | |  |  |
| On a scale of 1 to 10, how would you rate the degree of implementation of the individual intervention components at the present time? (1=no implementation noticeable … 10=fully implemented)  Please note any difficulties. (free text) | self-developed, based on Richter et al. [2] |  |  |  |  |  |  |  |  | M | |  |  |
| Mode of contact  (telepfone, e-mail, other (free text)) | self-developed |  |  |  |  |  |  |  |  |  |  | M | |
| Over the past months, you have been regularly supervised by a study team member in your work as *interprof* ACT agent or substitute, including phone calls and face-to-face meetings: How useful was this supervision for you?  (1=very useful … 6=not at all useful) | self-developed |  |  |  |  |  |  |  |  |  | Q |  |  |
| **IPAV work** |  |  |  |  |  |  |  |  |  |  |  |  |  |
| What activities have you undertaken or planned to undertake to support the implementation of the intervention? Rate your answer for each of the following activities:   - Discussions with the nursing team involved - Discussions with the designated contact person in nursing home - Discussions with the participating GPs - Discussions with the practice staff or the participating GPs' offices - Discussions with the nursing home residents - Discussions with relatives - Discussions with the nursing home director or management - Accompanying care processes or GPs' home visits in the nursing home - Organization of materials, e.g. name badges or "pro re nata medication" forms - Clarification of technical questions, e.g. about fax or telecommunication with GPs’ offices - Clarification of technical questions, e.g. about fax or telecommunication with GPs’ offices - Specialist training for individual RN (e.g. geriatrics) - Joint case discussions - Joint treatment pathways - Regular meetings between IPAV and nursing home director or management - Recruitment of additional nursing staff - Creation of time resources for IPAV work   (1=yes, already executed, 2=no, but planned; 3=no, and not yet planned) | self-developed |  |  |  |  |  |  |  |  | Q |  |  |  |
| In your opinion, how important have the following activities been in supporting the implementation of the intervention in the last 6 months?   - Discussions with the nursing team involved - Discussions with the designated contact person in nursing home - Discussions with the participating GPs - … (same activities as listed for the T1 item above)   (1=very important … 6=absolutely not important) | self-developed |  |  |  |  |  |  |  |  |  | Q |  |  |
| **Implementational work within a team** |  |  |  |  |  |  |  |  |  |  |  |  |  |
| Coherence |  |  |  |  |  |  |  |  |  |  |  |  |  |
| The nursing team is aware of the *interprof* ACT intervention package that has been introduced. (1=strongly agree ... 5=strongly disagree) | NoMAD (adapted) |  |  |  |  |  |  |  |  | Q | Q |  |  |
| Cognitive participation |  |  |  |  |  |  |  |  |  |  |  |  |  |
| RNs adhere to the agreements made at the kick-off meeting.  (1=strongly agree ... 5=strongly disagree) | NoMAD (adapted) |  |  |  |  |  |  |  |  | Q | Q |  |  |
| GPs adhere to the agreements made at the kick-off meeting.  (1=strongly agree ... 5=strongly disagree) | NoMAD (adapted) |  |  |  |  |  |  |  |  | Q | Q |  |  |
| Nursing home directors adhere to the agreements made at the kick-off meeting.  (1=strongly agree ... 5=strongly disagree) | NoMAD (adapted) |  |  |  |  |  |  |  |  | Q | Q |  |  |
| I will continue to support the implementation of the *interprof* ACT intervention package.  (1=strongly agree ... 5=strongly disagree) | NoMAD (adapted) |  |  |  |  |  |  |  | Q |  | Q |  |  |
| RNs will continue to support the implementation of the *interprof* ACT intervention package.  (1=strongly agree ... 5=strongly disagree) | NoMAD (adapted) |  |  |  |  |  |  |  | Q |  | Q |  |  |
| Collective action |  |  |  |  |  |  |  |  |  |  |  |  |  |
| The *interprof* ACT intervention package and its implementation are regularly addressed in team meetings.  (1=strongly agree ... 5=strongly disagree) | self-developed |  |  |  |  |  |  |  |  | Q | Q |  |  |
| The *interprof* ACT intervention package could be fully integrated into the existing work routine as agreed in the kick-off meeting.  (1=strongly agree ... 5=strongly disagree) | NoMAD (adapted) |  |  |  |  |  |  |  |  | Q | Q |  |  |
| I feel well supported by the management in my work for the *interprof* ACT intervention package.  (1=strongly agree ... 5=strongly disagree) | NoMAD (adapted) |  |  |  |  |  |  |  |  | Q | Q |  |  |
| I can easily integrate the *interprof* ACT intervention package into my existing work.  (1=strongly agree ... 5=strongly disagree) | NoMAD (original) |  |  |  |  |  |  |  | Q |  | Q |  |  |
| RNs can easily integrate the *interprof* ACT intervention package into their existing work.  (1=strongly agree ... 5=strongly disagree) | NoMAD (adapted) |  |  |  |  |  |  |  | Q |  | Q |  |  |
| Reflexive monitoring |  |  |  |  |  |  |  |  |  |  |  |  |  |
| The staff agree that the *interprof* ACT intervention package is worthwhile.  (1=strongly agree ... 5=strongly disagree) | NoMAD (original) |  |  |  |  |  |  |  |  | Q | Q |  |  |

Abbreviations: *GP,* general practitioner; *IPAV, interprof* ACT agent*; M,* minutes; *Mo,* moderator; *NH,* nursing home; *NHD,* nursing home director; *NHR,* nursing home resident; *P,* participant; *Q,* questionnaire; *RN,* registered nurse; *STM,* study team member*; T0b,* shortly post randomization; *T1,* follow-up after 6 months; *T2,* follow-up after 12 months.

Instruments: *NoMAD* Normalization Measure Development [3, 4].

**Table 2.2** Items of questionnaires and minutes for the outcome domain “implementation of *interprof* ACT components”

| **Domain,** Subdomain and/or *dimensions* | **Source** | **Target population at specific measurement points** | | | | | | | | | | | |
| --- | --- | --- | --- | --- | --- | --- | --- | --- | --- | --- | --- | --- | --- |
| Items of questionnaires/minutes |  | **NHD** | | **NHR** | | **GP** | | **RN** | | **IPAV** | | **STM** | |
|  |  | **T0a** | **T2** | **T0a** | **T2** | **T0a** | **T2** | **T0a** | **T2** | **T1** | **T2** | **T0a** | **T1–T2** |
| **Name badges** |  |  |  |  |  |  |  |  |  |  |  |  |  |
| *Choice and adaptation (only IG)* |  |  |  |  |  |  |  |  |  |  |  |  |  |
| Choice and adaptation: see kick-off meeting |  |  |  |  |  |  |  |  |  |  |  | M (kick-off) | |
| Degree of implementation and adaptations: see supervision |  |  |  |  |  |  |  |  |  | M (supervision) | |  |  |
| *Current practice* |  |  |  |  |  |  |  |  |  |  |  |  |  |
| The GP wears a name badge during the home visit.  (1=always ... 5=never) | self-developed | Q | Q |  |  |  |  |  |  |  |  |  |  |
| RNs wear name badges visible.  (1=always ... 5=never) | self-developed | Q | Q |  |  |  |  |  |  |  |  |  |  |
| How often do you wear a name badge?  (1=very often ... 5=never) | self-developed |  |  |  |  |  |  | Q* | Q* |  |  |  |  |
| How often do you/GP wear a name badge when you/GP visit the nursing home for a home visit?  (1=very often ... 5=never) | self-developed |  |  |  |  | Q | Q | Q* | Q* |  |  |  |  |
| **Mandatory availability rules** |  |  |  |  |  |  |  |  |  |  |  |  |  |
| *Choice and adaptation (only IG)* |  |  |  |  |  |  |  |  |  |  |  |  |  |
| Choice and adaptation: see kick-off meeting |  |  |  |  |  |  |  |  |  |  |  | M (kick-off) | |
| Degree of implementation and adaptations: see supervision |  |  |  |  |  |  |  |  |  | M (supervision) | |  |  |
| *Attitudes* |  |  |  |  |  |  |  |  |  |  |  |  |  |
| How important do you think it is that there are clear regulations on the availability of GPs?**  (1=very important ... 6=absolutely not important) | self-developed |  |  |  |  | Q | Q | Q | Q |  |  |  |  |
| How important do you think it is that there are clear regulations for responsibility of GPs at night and at weekends?**  (1=very important ... 6=absolutely not important) | self-developed |  |  |  |  | Q | Q | Q | Q |  |  |  |  |
| *Current practice* |  |  |  |  |  |  |  |  |  |  |  |  |  |
| How often do you not reach the RN/GP in charge of the nursing home resident at your first call?**  (1=very often ... 5=never) | InDemA (original) |  |  |  |  | Q | Q | Q* | Q* |  |  |  |  |
| In urgent cases: How often can you be reached immediately by phone by the responsible RN/GP during practice hours?**  (1=very often ... 5=never) | Koverdem (adapted) |  |  |  |  | Q | Q | Q* | Q* |  |  |  |  |
| In urgent cases: How often does it happen that you reach the GP during out of office hours?** (1=very often ... 5=never) | Koverdem (adapted) |  |  |  |  |  |  | Q* | Q* |  |  |  |  |
| *Quality and satisfaction* |  |  |  |  |  |  |  |  |  |  |  |  |  |
| The availability of GPs by fax has …  (1=markedly improved ... 5=markedly worsened) | self-developed |  | Q |  |  |  |  |  |  |  |  |  |  |
| The availability of GPs by phone has …  (1=markedly improved … 5=markedly worsened) | self-developed |  | Q |  |  |  | Q |  | Q* |  | Q |  |  |
| How satisfied are you with the existing arrangements for medical responsibility at night and at weekends?  (1=absolutely ... 5=absolutely not) | self-developed |  |  |  |  | Q | Q | Q* | Q* | Q | Q |  |  |
| **Designated contact persons** |  |  |  |  |  |  |  |  |  |  |  |  |  |
| *Choice and adaptation (only IG)* |  |  |  |  |  |  |  |  |  |  |  |  |  |
| Choice and adaptation: see kick-off meeting |  |  |  |  |  |  |  |  |  |  |  | M (kick-off) | |
| Degree of implementation and adaptations: see supervision |  |  |  |  |  |  |  |  |  | M (supervision) | |  |  |
| *Attitudes* |  |  |  |  |  |  |  |  |  |  |  |  |  |
| How important do you think it is that there are designated contact persons for GPs from the team of RNs? (1=very important ... 6=absolutely not important) | self-developed |  |  |  |  | Q | Q | Q | Q |  |  |  |  |
| *Current practice* |  |  |  |  |  |  |  |  |  |  |  |  |  |
| Designated contact persons are nominated for the GP in nursing home wards.  (1=always ... 5=never) | InDemA (adapted) | Q | Q |  |  |  |  |  |  |  |  |  |  |
| How often a designated contact person has been nominated for you in the nursing home ward of interest?  (1=very often ... 5=never) | InDemA (original) |  |  |  |  | Q | Q |  |  |  |  |  |  |
| *Quality and satisfaction* |  |  |  |  |  |  |  |  |  |  |  |  |  |
| How satisfied are you with the transparency of the designated contact persons in the nursing home?  (1=absolutely ... 5=absolutely not) | self-developed |  |  |  |  | Q | Q |  |  |  |  |  |  |
| **Standardized GPs’ home visits** |  |  |  |  |  |  |  |  |  |  |  |  |  |
| *Choice and adaptation (only IG)* |  |  |  |  |  |  |  |  |  |  |  |  |  |
| Choice and adaptation: see kick-off meeting |  |  |  |  |  |  |  |  |  |  |  | M (kick-off) | |
| Degree of implementation and adaptations: see supervision |  |  |  |  |  |  |  |  |  | M (supervision) | |  |  |
| *Attitudes* |  |  |  |  |  |  |  |  |  |  |  |  |  |
| I consider a joint ward round with the responsible RN/GP to be useful.  (1=strongly agree ... 4=disagree) | self-developed |  |  |  |  | Q | Q | Q | Q |  |  |  |  |
| How important do you think it is that there are prepared ward rounds, e.g. where information relevant to the home visit is collected in advance by a RN and listed point by point?  (1=very important ... 6=absolutely not important) | self-developed |  |  |  |  | Q | Q | Q | Q |  |  |  |  |
| *Current practice* |  |  |  |  |  |  |  |  |  |  |  |  |  |
| GPs home visits take place at fixed frequencies.**  (1=always ... 5=never) | self-developed | Q | Q |  |  |  |  |  |  |  |  |  |  |
| A RN is the designated contact person for the GP during home visits.**  (1=always ... 5=never) | self-developed | Q | Q |  |  |  |  |  |  |  |  |  |  |
| The responsible RN is always released from other tasks when the GPs home visit takes place.**  (1=always ... 5=never) | self-developed | Q | Q |  |  |  |  |  |  |  |  |  |  |
| When the GP visits the nursing home resident, it is arranged for a RN to be present.**  (1=always ... 5=never) | self-developed | Q | Q |  |  |  |  |  |  |  |  |  |  |
| How often fix appointments for the nursing home visits are being agreed on by you and the nursing staff of the nursing home ward in advance of the visit?**  (1=very often ... 5=never) | self-developed |  |  |  |  | Q | Q | Q* | Q* |  |  |  |  |
| If there are no fixed appointments, how often do you/the GP give at least one day a priori notice of your/the GPs home visit?**  (1=very often ... 5=never) | InDemA (original) |  |  |  |  | Q | Q | Q* | Q* |  |  |  |  |
| How often is there sufficient time in the nursing home wards to discuss current treatment issues with the RNs?**  (1=very often ... 5=never) | InDemA (original) |  |  |  |  | Q | Q | Q* | Q* |  |  |  |  |
| How often do you/the GP records prescriptions in the nursing home residents’ documentation?**  (1=very often ... 5=never) | InDemA (original) |  |  |  |  | Q | Q | Q* | Q* |  |  |  |  |
| How often do home visits take place on a regular basis?**  (1=very often ... 5=never) | self-developed |  |  |  |  | Q | Q |  |  |  |  |  |  |
| How often do you initiate home visits without a fixed frequency?**  (1=very often ... .5=never) | self-developed |  |  |  |  | Q | Q |  |  |  |  |  |  |
| How often do RN initiate home visits without a fixed frequency?**  (1=very often ... 5=never) | self-developed |  |  |  |  | Q | Q |  |  |  |  |  |  |
| *Quality and satisfaction* |  |  |  |  |  |  |  |  |  |  |  |  |  |
| How satisfied are you with the frequency of your GP’s home visits?  (1=very satisfied ... 4=very dissatisfied) | self-developed |  |  | Q | Q |  |  |  |  |  |  |  |  |
| Appointments for GPs home visits have …**  (1=markedly improved … 5=markedly worsened) | self-developed |  | Q |  |  |  | Q |  | Q* |  | Q |  |  |
| The preparation of home visits has ...**  (1=markedly improved … 5=markedly worsened) | self-developed |  |  |  |  |  | Q |  | Q* |  | Q |  |  |
| The procedure for home visits (incl. discussion before and after the visit with the GPs) has …**  (1=markedly improved … 5=markedly worsened) | self-developed |  |  |  |  |  | Q |  | Q* |  | Q |  |  |
| How satisfied are you with the procedure of the home visit in your nursing home?  (1=absolutely ... 5=absolutely not) | self-developed |  |  |  |  | Q | Q | Q* | Q* | Q | Q |  |  |
| The possibility for GP to document prescriptions on site in the nursing home has ...  (1=markedly improved … 5=markedly worsened) | self-developed |  |  |  |  |  | Q |  | Q |  | Q |  |  |
| **Pro re nata medication** |  |  |  |  |  |  |  |  |  |  |  |  |  |
| *Choice and adaptation (only IG)* |  |  |  |  |  |  |  |  |  |  |  |  |  |
| Choice and adaptation: see kick-off meeting |  |  |  |  |  |  |  |  |  |  |  | M (kick-off) | |
| Degree of implementation and adaptations: see supervision |  |  |  |  |  |  |  |  |  | M (supervision) | |  |  |
| *Quality and satisfaction* |  |  |  |  |  |  |  |  |  |  |  |  |  |
| The safety of prescribing and administering medication has ...  (1=markedly improved … 5=markedly worsened) | self-developed |  | Q |  |  |  |  |  | Q |  | Q |  |  |
| How satisfied are you with the completeness of the information provided in GPs’ prescription for regular and pro re nata medication (indication, dose, duration, form of administration, any side effects to be observed)?**  (1=absolutely ... 5=absolutely not) | self-developed |  |  |  |  |  |  | Q* | Q* | Q | Q |  |  |
| How satisfied are you with the readability of GPs’ prescriptions, e.g. for regular and pro re nata medication?**  (1=absolutely ... 5=absolutely not) | self-developed |  |  |  |  |  |  | Q* | Q* | Q | Q |  |  |
| How satisfied are you with the timing of the communication by GPs in the case of new or changed prescriptions, e.g. for regular or pro re nata medication?**  (1=absolutely ... 5=absolutely not) | self-developed |  |  |  |  |  |  | Q* | Q* | Q | Q |  |  |
| How satisfied are you with the transparency of medication applications (incl. pro re nata medication) by the RNs? (1=absolutely ... 5=absolutely not) | self-developed |  |  |  |  | Q | Q |  |  |  |  |  |  |
| **Shared goal setting** |  |  |  |  |  |  |  |  |  |  |  |  |  |
| *Choice and adaptation (only IG)* |  |  |  |  |  |  |  |  |  |  |  |  |  |
| Choice and adaptation: see kick-off meeting |  |  |  |  |  |  |  |  |  |  |  | M (kick-off) | |
| Degree of implementation and adaptations: see supervision |  |  |  |  |  |  |  |  |  | M (supervision) | |  |  |
| *Attitudes* |  |  |  |  |  |  |  |  |  |  |  |  |  |
| How important do you think it is that there are clear patient-individual agreements per resident with the RNs/GPs in the nursing home on how to proceed in emergency situations?  (1=very important ... 6=absolutely not important) | self-developed |  |  |  |  | Q | Q | Q | Q |  |  |  |  |
| *Current practice* |  |  |  |  |  |  |  |  |  |  |  |  |  |
| RNs and GPs jointly agree on goals for each nursing home resident to be cared for.  (1=strongly agree ... 5=strongly disagree) | self-developed | Q | Q |  |  |  |  |  |  |  |  |  |  |
| How often do you make shared arrangements for the care for nursing home residents together with the responsible RN/GP?**  ( 1=very often ... 5=never) | InDemA (original) |  |  |  |  | Q | Q | Q* | Q* |  |  |  |  |
| How often do you agree with the decisions made by the interprofessional team?**  ( 1=very often ... 5=never) | Koverdem (adapted) |  |  |  |  | Q | Q |  |  |  |  |  |  |
| How often does it happen that decisions are being made about the medical care for your patients in the nursing home without involving you?**  ( 1=very often ... 5=never) | PSAT (adapted) |  |  |  |  | Q | Q |  |  |  |  |  |  |
| *Quality and satisfaction* |  |  |  |  |  |  |  |  |  |  |  |  |  |
| The joint planning of goals for nursing home residents by RNs and GPs has ...  (1=markedly improved … 5=markedly worsened) | self-developed |  | Q |  |  |  |  |  |  |  |  |  |  |
| How satisfied are you with existing joint agreements to achieve the goals?**  (1=absolutely ... 5=absolutely not) | PSAT (adapted) |  |  |  |  | Q | Q | Q* | Q* | Q | Q |  |  |
| How satisfied are you with the ways how decisions are being made in the collaboration?**  (1=absolutely ... 5=absolutely not) | PSAT (adapted) |  |  |  |  | Q | Q | Q* | Q* | Q | Q |  |  |

*Questions from part B questionnaire of registered nurses related to questions for several specific GPs, i.e. registered nurses rated these questions separately for multiple GPs (see Table 2.9).

**Items were summarized as sum score for data analysis.

Abbreviations: *GP,* general practitioner; *IPAV, interprof* ACT agent*; M,* minutes; *NH,* nursing home; *NHD,* nursing home director; *NHR,* nursing home resident; *Q,* questionnaire; *RN,* registered nurse; *STM,* study team member*; T0a,* baseline assessment (before randomized allocation); *T1,* follow-up after 6 months; *T2,* follow-up after 12 months.

Instruments: *InDemA* Interdisciplinary Implementation of Quality Instruments for the Care of Residents with Dementia in Nursing Homes ; *Koverdem* Survey instruments of the research project “Optimizing Cooperation between General Practitioners and Home Care Services” [7]; *PSAT* Partnership Self-Assessment Tool [8].

**Table 2.3** Items of questionnaires and minutes for the outcome domain “key elements of interprofessional collaboration”

| **Domain,** Subdomain and/or *dimensions* | **Source** | **Target population at specific measurement points** | | | | | | | | | | | |
| --- | --- | --- | --- | --- | --- | --- | --- | --- | --- | --- | --- | --- | --- |
| Items of questionnaires/minutes |  | **NHD** | | **NHR** | | **GP** | | **RN** | | **IPAV** | | **STM** | |
|  |  | **T0a** | **T2** | **T0a** | **T2** | **T0a** | **T2** | **T0a** | **T2** | **T1** | **T2** | **T0a** | **T1–T2** |
| **Involvement of NHR** |  |  |  |  |  |  |  |  |  |  |  |  |  |
| *Current practice* |  |  |  |  |  |  |  |  |  |  |  |  |  |
| The wishes and needs of the nursing home residents are the most important.  (1=strongly agree … 5=strongly disagree) | self-developed | Q | Q |  |  |  |  |  |  |  |  |  |  |
| *Quality and satisfaction* |  |  |  |  |  |  |  |  |  |  |  |  |  |
| Nursing home residents involvement in decisions on medical care has …  (1=markedly improved … 5=markedly worsened) | self-developed |  | Q |  |  |  |  |  |  |  |  |  |  |
| How satisfied are you with the time the GP takes for you?  (1=very satisfied … 4=very dissatisfied) | ZAP (original) |  |  | Q | Q |  |  |  |  |  |  |  |  |
| How satisfied are you with the information given by your GP about your health conditions and treatments?  (1=very satisfied … 4=very dissatisfied) | ZAP (adapted) |  |  | Q | Q |  |  |  |  |  |  |  |  |
| How often does your GP talk to you about the advantages and disadvantages of different treatment options?  (1=always … 4=never) | ZAP (adapted) |  |  | Q | Q |  |  |  |  |  |  |  |  |
| How often does your GP explain the given information to you in an easily to understand way?  (1=always … 4=never) | ZAP (adapted) |  |  | Q | Q |  |  |  |  |  |  |  |  |
| How often does your GP involves you as much as you would like in decisions about your treatment?  (1=always … 4=never) | ZAP (adapted) |  |  | Q | Q |  |  |  |  |  |  |  |  |
| Nursing home resident involvement in decisions about medical care in the nursing home has …  (1=markedly improved … 5=markedly worsened) | self-developed |  |  |  |  |  | Q |  | Q |  | Q |  |  |
| **Interprofessional communication** |  |  |  |  |  |  |  |  |  |  |  |  |  |
| *Attitudes* |  |  |  |  |  |  |  |  |  |  |  |  |  |
| There is a high level of willingness among RNs to communicate with GPs.  (1=fully applicable … 5=fully not applicable) | self-developed |  |  |  |  |  |  |  |  | Q | Q |  |  |
| There is a high level of willingness among GPs to communicate with RNs.  (1=fully applicable … 5=fully not applicable) | self-developed |  |  |  |  |  |  |  |  | Q | Q |  |  |
| *Current practice* |  |  |  |  |  |  |  |  |  |  |  |  |  |
| When you think about the medical care in the nursing home, how often do you feel that the RNs and your GP discuss your health and your treatment?  (1=always … 4=never) | Koverdem (adapted) |  |  | Q | Q |  |  |  |  |  |  |  |  |
| *Quality and satisfaction* |  |  |  |  |  |  |  |  |  |  |  |  |  |
| It is easy for RNs to communicate openly with GPs.  (1=fully applicable … 5=fully not applicable) | self-developed |  |  |  |  |  |  |  |  | Q | Q |  |  |
| RNs and GPs do not always communicate openly with each other.  (1=fully applicable … 5=fully not applicable) | self-developed |  |  |  |  |  |  |  |  | Q | Q |  |  |
| RNs and GPs are not always cooperative with each other.  (1=fully applicable … 5=fully not applicable) | self-developed |  |  |  |  |  |  |  |  | Q | Q |  |  |
| How satisfied are you with the general quality of communication between you and the GP/RN in this nursing home?  (1=absolutely … 5=absolutely not) | self-developed |  |  |  |  |  | Q |  | Q* |  | Q |  |  |
| The general quality of communication with the GP/RN in the nursing home has …  (1=markedly improved … 5=markedly worsened) | self-developed |  |  |  |  |  | Q |  | Q* |  | Q |  |  |
| The communication with the GP/RN by phone and fax has …  (1=markedly improved … 5=markedly worsened) | self-developed |  |  |  |  |  | Q |  | Q* |  | Q |  |  |
| The flow of information between RNs and GPs has …  (1=markedly improved … 5=markedly worsened) | self-developed |  | Q |  |  |  | Q |  | Q* |  | Q |  |  |
| The communication between me and the RNs/GPs is characterized by appreciation and mutual trust.**  (1=strongly agree … 4=disagree) | self-developed |  |  |  |  |  | Q |  | Q* |  |  |  |  |
| The communication between me and RNs/GPs is characterized by openness.**  (1=strongly agree … 4=disagree) | CPAT (adapted) |  |  |  |  |  | Q |  | Q* |  |  |  |  |
| In the communication between me and the RNs/GPs, suspected or observed faults relating to the care of the nursing home residents are addressed openly and constructively, and there are opportunities to learn from these faults.**  (1=strongly agree … 4=disagree) | CPAT (adapted) |  |  |  |  |  | Q |  | Q* |  |  |  |  |
| Ways are jointly identified to avoid faults in the future and to improve care.**  (1=strongly agree … 4=disagree) | CPAT (adapted) |  |  |  |  |  | Q |  | Q* |  |  |  |  |
| **Contribution of involved professions** |  |  |  |  |  |  |  |  |  |  |  |  |  |
| *Attitudes* |  |  |  |  |  |  |  |  |  |  |  |  |  |
| I have an equal relationship with colleagues in my own discipline.  (1=fully applicable … 5=fully not applicable) | self-developed |  |  |  |  |  |  |  |  | Q | Q |  |  |
| There are many overlapping areas of responsibility between GPs and RNs.**  (1=strongly agree … 4=disagree) | Jefferson Scale (original) |  |  |  |  | Q | Q | Q | Q |  |  |  |  |
| RNs should also have responsibility for monitoring the effects of medical treatments.**  (1=strongly agree … 4=disagree) | Jefferson Scale (original) |  |  |  |  | Q | Q | Q | Q |  |  |  |  |
| RNs should be accountably to nursing home residents for the medical care tasks delegated by GPs.**  (1=strongly agree … 4=disagree) | Jefferson Scale (adapted) |  |  |  |  | Q | Q | Q | Q |  |  |  |  |
| GPs and RNs should jointly decide on hospital admissions of nursing home residents.  (1=strongly agree … 4=disagree) | Jefferson Scale (adapted) |  |  |  |  | Q | Q | Q | Q |  |  |  |  |
| RNs should critically review and clarify GPs’ order when they feel that it might have the potential for detrimental effects on the nursing home resident.  (1=strongly agree … 4=disagree) | Jefferson Scale (adapted) |  |  |  |  | Q | Q | Q | Q |  |  |  |  |
| The most important function of RNs is to execute the GP’s instructions.**  (1=strongly agree … 4=disagree) | Jefferson Scale (adapted) |  |  |  |  | Q | Q | Q | Q |  |  |  |  |
| GPs should be the dominant authority in all matters of nursing home residents’ care.**  (1=strongly agree … 4=disagree) | Jefferson Scale (adapted) |  |  |  |  | Q | Q | Q | Q |  |  |  |  |
| *Current practice* |  |  |  |  |  |  |  |  |  |  |  |  |  |
| How often do RNs adhere to shared care decisions?  (1=very often … 5=never) | InDemA (original) |  |  |  |  | Q | Q |  |  |  |  |  |  |
| How often are your prescriptions implemented in a timely and reliable manner?  (1=very often … 5=never) | InDemA (original) |  |  |  |  | Q | Q |  |  |  |  |  |  |
| How often are nursing home residents’ concerns realized in a timely and reliable manner by the GP?  (1=very often … 5=never) | InDemA (adapted) |  |  |  |  |  |  | Q* | Q* |  |  |  |  |
| How often do you have the impression that relevant changes in nursing home residents’ health are overlooked or misinterpreted by the team of RNs in the nursing home?  (1=very often … 5=never) | CPAT (adapted) |  |  |  |  |  | Q |  |  |  |  |  |  |
| How often do you have the impression that relevant information about the medical treatment of nursing home residents (e.g. prescriptions) is ‘lost’ by the nursing staff/in the GPs’ office?  (1=very often … 5=never) | CPAT (adapted) |  |  |  |  |  | Q |  | Q* |  |  |  |  |
| *Quality and satisfaction* |  |  |  |  |  |  | Q |  |  |  |  |  |  |
| How satisfied are you with your influence in the partnership?  (1=absolutely … 5=absolutely not) | PSAT (adapted) |  |  |  |  | Q | Q | Q* | Q* | Q | Q |  |  |
| How satisfied are you with the distribution of tasks between RNs and GPs in the medical care of nursing home residents in your nursing home?  (1=absolutely … 5=absolutely not) | self-developed |  |  |  |  | Q | Q | Q | Q |  | Q |  |  |
| The implementation of medical prescriptions has …  (1=markedly improved … 5=markedly worsened) | self-developed |  |  |  |  |  | Q |  | Q |  | Q |  |  |
| How satisfied are you with your own role within the collaboration?  (1=absolutely … 5=absolutely not) | PSAT (adapted) |  |  |  |  | Q | Q | Q* | Q* |  |  |  |  |
| How satisfied are you with the timeliness of the communication of information on the state of health of the nursing home residents you care for in this nursing home?  (1=absolutely … 5=absolutely not) | CPAT (adapted) |  |  |  |  | Q | Q |  |  |  |  |  |  |
| How satisfied are you with the relevance and accuracy of the written and verbal information you receive from the nursing staff about the state of health of the nursing home residents you care for in this nursing home?  (1=absolutely … 5=absolutely not) | self-developed |  |  |  |  | Q | Q |  |  |  |  |  |  |
| **Coordination of care decisions and care planning** |  |  |  |  |  |  |  |  |  |  |  |  |  |
| *Attitudes* |  |  |  |  |  |  |  |  |  |  |  |  |  |
| I consider fixed phone consultations with the RN/GP to be useful.  (1=strongly agree … 4=disagree) | InDemA (adapted) |  |  |  |  | Q | Q | Q | Q |  |  |  |  |
| How important is it for you that there are jointly developed, shared pathways/algorithms for making decisions about hospital admissions?  (1=very important … 6=absolutely not important) | self-developed |  |  |  |  | Q | Q | Q | Q |  |  |  |  |
| *Current practice* |  |  |  |  |  |  |  |  |  |  |  |  |  |
| There are ongoing interprofessional discussions about the care of nursing home residents.  (1=strongly agree … 5=strongly disagree) | self-developed | Q | Q |  |  |  |  |  |  |  |  |  |  |
| How often do you contact the nursing home?  (1=very often … 5=never) | self-developed |  |  |  |  | Q | Q |  |  |  |  |  |  |
| How often does the practice staff contact the nursing home?  (1=very often … 5=never) | self-developed |  |  |  |  | Q | Q |  |  |  |  |  |  |
| How often do you contact the GP?  (1=very often … 5=never) | self-developed |  |  |  |  |  |  | Q* | Q* |  |  |  |  |
| How often do RNs ask you to make an emergency home visit of a nursing home resident?  (1=very often … 5=never) | self-developed |  |  |  |  | Q | Q |  |  |  |  |  |  |
| In retrospect, how often was it necessary for you to make an emergency home visit if RNs ask you?  (1=very often … 5=never) | InDemA (original) |  |  |  |  | Q | Q |  |  |  |  |  |  |

*Questions from part B questionnaire of registered nurses related to questions for several specific GPs, i.e. registered nurses rated these questions separately for multiple GPs (see Table 2.9).

**Items were summarized as sum score for data analysis.

Abbreviations: *GP,* general practitioner; *IPAV, interprof* ACT agent*; NH,* nursing home; *NHD,* nursing home director; *NHR,* nursing home resident; *Q,* questionnaire; *RN,* registered nurse; *STM,* study team member*; T0a,* baseline assessment (before randomized allocation); *T1,* follow-up after 6 months; *T2,* follow-up after 12 months.

Instruments: *CPAT* Collaborative Practice Assessment Tool [9]*; InDemA* Interdisciplinary Implementation of Quality Instruments for the Care of Residents with Dementia in Nursing Homes ; *Jefferson Scale* of Attitudes toward Physician–Nurse Collaboration [10]; *Koverdem* Survey instruments of the research project “Optimizing Cooperation between General Practitioners and Home Care Services” [7]; *NoMAD* Normalization Measure Development [3, 4]; *PSAT* Partnership Self-Assessment Tool [8]; *ZAP* Zufriedenheit in der Arztpraxis (“Satisfaction in GPs Office”) [11].

**Table 2.4** Items of questionnaires and minutes for the outcome domain “further domains related to interprofessional collaboration and medical care”

| **Domain,** Subdomain and/or *dimensions* | **Source** | **Target population at specific measurement points** | | | | | | | | | | | |
| --- | --- | --- | --- | --- | --- | --- | --- | --- | --- | --- | --- | --- | --- |
| Items of questionnaires/minutes |  | **NHD** | | **NHR** | | **GP** | | **RN** | | **IPAV** | | **STM** | |
|  |  | **T0a** | **T2** | **T0a** | **T2** | **T0a** | **T2** | **T0a** | **T2** | **T1** | **T2** | **T0a** | **T1–T2** |
| **General interprofessional collaboration** |  |  |  |  |  |  |  |  |  |  |  |  |  |
| *Attitudes* |  |  |  |  |  |  |  |  |  |  |  |  |  |
| Overall, RNs and GPs have the same understanding of interprofessional collaboration.  (1=strongly agree … 5=strongly disagree) | self-developed | Q | Q |  |  |  |  |  |  |  |  |  |  |
| *Quality and satisfaction* |  |  |  |  |  |  |  |  |  |  |  |  |  |
| In general, cooperation with the GPs works smoothly.  (1=strongly agree … 5=strongly disagree) | self-developed | Q | Q |  |  |  |  |  |  |  |  |  |  |
| Collaboration between the GPs and RNs has …  (1=markedly improved … 5=markedly worsened) | self-developed |  | Q |  |  |  |  |  |  |  |  |  |  |
| How satisfied are you with the collaboration with the RNs/GPs in general? (1=absolutely … 5=absolutely not) | InDemA (original) |  |  |  |  | Q | Q | Q* | Q* | Q | Q |  |  |
| How satisfied are you with the recognition and appreciation by the RNs/GPs in general?  (1=absolutely … 5=absolutely not) | InDemA (original) |  |  |  |  | Q | Q | Q* | Q* | Q | Q |  |  |
| How satisfied are you with the effectiveness of the collaboration (effort versus benefit)?  (1=absolutely … 5=absolutely not) | InDemA (adapted) |  |  |  |  | Q | Q | Q* | Q* | Q | Q |  |  |
| The general quality of collaboration with the GPs/RNs in the nursing home has …  (1=markedly improved … 5=markedly worsened) | self-developed |  |  |  |  |  | Q |  | Q* |  | Q |  |  |
| **General quality of (medical) care for nursing home resident** |  |  |  |  |  |  |  |  |  |  |  |  |  |
| *Quality and satisfaction* |  |  |  |  |  |  |  |  |  |  |  |  |  |
| In my experience, the quality of GP care for nursing home residents is good.  (1=strongly agree … 5=strongly disagree) | self-developed | Q | Q |  |  |  |  |  |  |  |  |  |  |
| The general quality of care for residents has …  (1=markedly improved … 5=markedly worsened) | self-developed |  | Q |  |  |  | Q |  | Q |  | Q |  |  |
| In general, how satisfied are you with the care provided by your GP?  (1=very satisfied … 4=very dissatisfied) | ZAP (adapted) |  |  | Q | Q |  |  |  |  |  |  |  |  |
| If you think of the medical care you receive here in the nursing home: How often do you have the impression that you receive the medication and therapeutic interventions as prescribed by your GP?  (1=always … 4=never) | self-developed |  |  | Q | Q |  |  |  |  |  |  |  |  |
| If you think of the medical care you receive here in the nursing home: How often do you have the impression that you get sufficient and timely help when you experience acute health problems?  (1=always … 4=never) | EUROPEP (adapted) |  |  | Q | Q |  |  |  |  |  |  |  |  |
| If you think of the medical care you receive here in the nursing home: How often do you have the impression that the collaboration between the RNs and your GP is for your well-being?  (1=always … 4=never) | self-developed |  |  | Q | Q |  |  |  |  |  |  |  |  |

*Questions from part B questionnaire of registered nurses related to questions for several specific GPs, i.e. registered nurses rated these questions separately for multiple GPs (see Table 2.9).

Abbreviations: *GP,* general practitioner; *IPAV, interprof* ACT agent*; NH,* nursing home; *NHD,* nursing home director; *NHR,* nursing home resident; *Q,* questionnaire; *RN,* registered nurse; *STM,* study team member*; T0a,* baseline assessment (before randomized allocation); *T1,* follow-up after 6 months; *T2,* follow-up after 12 months.

Instruments: *EUROPEP* Measures of the European Project on Patient Evaluation of General Practice Care [12]*; InDemA* Interdisciplinary Implementation of Quality Instruments for the Care of Residents with Dementia in Nursing Homes ; *ZAP* Zufriedenheit in der Arztpraxis (“Satisfaction in GPs Office”) [11].

**Table 2.5** Items of questionnaires and minutes for the outcome domain “context factors”

| **Domain,** Subdomain and/or *dimensions* | **Source** | **Target population at specific measurement points** | | | | | | | | | | | |
| --- | --- | --- | --- | --- | --- | --- | --- | --- | --- | --- | --- | --- | --- |
| Items of questionnaires/minutes |  | **NHD** | | **NHR** | | **GP** | | **RN** | | **IPAV** | | **STM** | |
|  |  | **T0a** | **T2** | **T0a** | **T2** | **T0a** | **T2** | **T0a** | **T2** | **T1** | **T2** | **T0a** | **T1–T2** |
| **Meso: organizational level** |  |  |  |  |  |  |  |  |  |  |  |  |  |
| Leadership and work environment |  |  |  |  |  |  |  |  |  |  |  |  |  |
| Readiness/commitment of nursing home directors to implementation  (1=very positive/helpful for implementation … 5=very negative/hindering for implementation) | self-developed |  |  |  |  |  |  |  |  | M (supervision) | |  |  |
| Collaboration with colleagues in the nursing team  (1=very positive/helpful for implementation ... 5=very negative/hindering for implementation) | self-developed |  |  |  |  |  |  |  |  | M (supervision) | |  |  |
| Readiness/commitment of colleagues in the nursing team to implementation  (1=very positive/helpful for implementation ... 5=very negative/hindering for implementation) | self-developed |  |  |  |  |  |  |  |  | M (supervision) | |  |  |
| Structures of collaboration and medical care procedures |  |  |  |  |  |  |  |  |  |  |  |  |  |
| *Current practice* |  |  |  |  |  |  |  |  |  |  |  |  |  |
| Cooperation with nursing home directors  (1=very positive/helpful for implementation ... 5=very negative/hindering for implementation) | self-developed |  |  |  |  |  |  |  |  | M (supervision) | |  |  |
| Collaboration with the GPs  (1=very positive/helpful for implementation ... 5=very negative/hindering for implementation) | self-developed |  |  |  |  |  |  |  |  | M (supervision) | |  |  |
| Readiness/commitment of GPs to implementation  (1=very positive/helpful for implementation ... 5=very negative/hindering for implementation) | self-developed |  |  |  |  |  |  |  |  | M (supervision) | |  |  |
| Experienced recognition from colleagues for my *interprof* ACT work  (1=very positive/helpful for implementation ... 5=very negative/hindering for implementation) | self-developed |  |  |  |  |  |  |  |  | M (supervision) | |  |  |
| Experienced recognition from nursing home directors for my *interprof* ACT work  (1=very positive/helpful for implementation ... 5=very negative/hindering for implementation) | self-developed |  |  |  |  |  |  |  |  | M (supervision) | |  |  |
| Experienced recognition from GPs for my *interprof* ACT work  (1=very positive/helpful for implementation ... 5=very negative/hindering for implementation) | self-developed |  |  |  |  |  |  |  |  | M (supervision) | |  |  |
| Experienced recognition from nursing home residents/relatives for my *interprof* ACT work  (1=very positive/helpful for implementation ... 5=very negative/hindering for implementation) | self-developed |  |  |  |  |  |  |  |  | M (supervision) | |  |  |
| *Financial and staff resources* |  |  |  |  |  |  |  |  |  |  |  |  |  |
| How satisfied are you with the number of RNs in your ward?  (1=absolutely ... 5=absolutely not) | self-developed |  |  |  |  |  |  | Q | Q |  | Q |  |  |
| How satisfied are you with the current remuneration of home visits/cost-benefit-ratio?  (1=absolutely ... 5=absolutely not) | self-developed |  |  |  |  | Q | Q |  |  |  |  |  |  |
| Number of RNs in the nursing team  (1=very positive/helpful for implementation ... 5=very negative/hindering for implementation) | self-developed |  |  |  |  |  |  |  |  | M (supervision) | |  |  |
| Stability of the nursing team  (1=very positive/helpful for implementation ... 5=very negative/hindering for implementation) | self-developed |  |  |  |  |  |  |  |  | M (supervision) | |  |  |
| Time resources for *interprof* ACT-work  (1=very positive/helpful for implementation ... 5=very negative/hindering for implementation) | self-developed |  |  |  |  |  |  |  |  | M (supervision) | |  |  |
| GP’s office and nursing home characteristics |  |  |  |  |  |  |  |  |  |  |  |  |  |
| Additional qualifications of medical assistants (e.g. wound management, management of emergencies, vaccination assistance) | self-developed |  |  |  |  | Q | Q |  |  |  |  |  |  |
| Size of practice/nursing home location  (>500,000; <500,000; 20,000–<100,000; 10,000–<20,000; <10,000; <5,000 inhabitants) | self-developed | Q |  |  |  | Q |  |  |  |  |  |  |  |
| Involvement in academic teaching (no/yes) | self-developed |  |  |  |  | Q | Q |  |  |  |  |  |  |
| Practice experience (years) | Koverdem (original) |  |  |  |  | Q |  |  |  |  |  |  |  |
| Number of nursing homes with nursing home residents cared for by this GP | Koverdem (adapted) |  |  |  |  | Q | Q |  |  |  |  |  |  |
| Cooperation agreements (yes/no)   - GP according to §119b Abs. 2 SGBV - GP-centered care according to §73b SGBV - Special medical care according to §140a SGBV - 24-hour availability of GPs every day of the week - No existing cooperation agreements - Others | self-developed | Q | Q |  |  | Q | Q |  |  |  |  |  |  |
| Ownership  (private, not-for-profit, public and church-affiliated) | self-developed | Q | Q |  |  |  |  |  |  |  |  |  |  |
| Number of (occupied) places in long-term care | self-developed | Q | Q |  |  |  |  |  |  |  |  |  |  |
| Number of nursing home residents with care levels 1/2/3/4/5* | self-developed | Q | Q |  |  |  |  |  |  |  |  |  |  |
| Number of RNs (fulltime equivalent) | self-developed | Q | Q |  |  |  |  |  |  |  |  |  |  |
| Number of wards | self-developed | Q | Q |  |  |  |  |  |  |  |  |  |  |
| Number of all GPs per nursing home |  | Q | Q |  |  |  |  |  |  |  |  |  |  |
| Has there been a quality inspection by the quality inspections by the Medical Review Board of the Statutory Health Insurance Funds (MD) in your nursing home within the last 12 months? (no/yes) | self-developed |  | Q |  |  |  |  |  |  |  |  |  |  |
| Have there been any structural changes in your nursing home in the last 12 months? (no/yes: fusion of two or more nursing homes, relocation of the nursing home, merging of nursing home wards, integration of new services (e.g. hospice/palliative care, short-term care), change of nursing home director) | self-developed |  | Q |  |  |  |  |  |  |  |  |  |  |
| **Micro: staff level** |  |  |  |  |  |  |  |  |  |  |  |  |  |
| Sociodemographic characteristics |  |  |  |  |  |  |  |  |  |  |  |  |  |
| Age (years) | self-developed |  |  |  |  | Q |  | Q | Q | Q (1^st^, 2^nd^, re-training) | | Q (kick-off, trainings) | |
| Sex (male, female) | self-developed |  |  |  |  | Q |  | Q | Q | Q (1^st^, 2^nd^, re-training) | | Q (kick-off, trainings) | |
| Practice ownership  (no/yes: single, together with co-owners, together with employed GPs) | self-developed |  |  |  |  | Q | Q |  |  |  |  |  |  |
| Estimated proportion (%) of nursing home residents among total number of patients | self-developed |  |  |  |  | Q | Q |  |  |  |  |  |  |
| Estimated hours per week required for medical care for nursing home residents | self-developed |  |  |  |  | Q | Q |  |  |  |  |  |  |
| Delegation of nursing home resident visits to medical assistants (yes/no) | self-developed |  |  |  |  | Q | Q |  |  |  |  |  |  |
| Delegated activities  (no/yes: venous blood collection, intramuscular injection, application of infusions, removal of suture material, changing the port needle, catheterization, inserting a transnasal tube, wound care, other (free text)) | self-developed |  |  |  |  | Q | Q |  |  |  |  |  |  |
| Type of first cycle nursing degree  (general nursing care, geriatric nursing care, others (free text)) | self-developed |  |  |  |  |  |  | Q | Q | Q (1^st^, 2^nd^, re-training) | |  |  |
| Professional experience as a GP/RN (years) | self-developed |  |  |  |  | Q |  | Q | Q | Q (1^st^, 2^nd^, re-training) | |  |  |
| Professional experience as a GP/RN in long-term care (years) | self-developed |  |  |  |  | Q |  | Q | Q | Q (1^st^, 2^nd^, re-training) | |  |  |
| Professional experience as a GP/RN in the participation nursing home (years) | self-developed |  |  |  |  |  |  | Q | Q | Q (1^st^, 2^nd^, re-training) | |  |  |
| Working hours per week | self-developed |  |  |  |  |  |  | Q | Q | Q (1^st^, 2^nd^, re-training) | |  |  |
| Additional nursing qualifications  (no/yes: management of a ward, geriatric-psychiatric care, palliative care, others (free text)) | self-developed |  |  |  |  |  |  | Q | Q | Q (1^st^, 2^nd^, re-training) | |  |  |
| Specialist qualification  (no/yes: general practice, internal medicine, psychiatry and psychotherapy, psychosomatic medicine and psychotherapy, others (free text)) | self-developed |  |  |  |  | Q |  |  |  |  |  |  |  |
| Competence of RNs, GPs and IPAVs |  |  |  |  |  |  |  |  |  |  |  |  |  |
| Medical and nursing competencies of colleagues in the nursing team  (1=very positive/helpful for implementation ... 5=very negative/hindering for implementation) | self-developed |  |  |  |  |  |  |  |  | M (supervision) | |  |  |
| Medical competencies of GPs  (1=very positive/helpful for implementation ... 5=very negative/hindering for implementation) | self-developed |  |  |  |  |  |  |  |  | M (supervision) | |  |  |
| My own professional experience and competencies  (1=very positive/helpful for implementation ... 5=very negative/hindering for implementation) | self-developed |  |  |  |  |  |  |  |  | M (supervision) | |  |  |
| Previous experiences/results of my *interprof* ACT work  (1=very positive/helpful for implementation ... 5=very negative/hindering for implementation) | self-developed |  |  |  |  |  |  |  |  | M (supervision) | |  |  |
| **Micro: NHR level** |  |  |  |  |  |  |  |  |  |  |  |  |  |
| Utilization of GP’s office (formal characteristics) |  |  |  |  |  |  |  |  |  |  |  |  |  |
| For how long have you already visited your current GP? (1=less than one year, 2=1 to 2 years, 3=3 to 5 years, 4=more than 5 years) | ZAP (adapted) |  |  | Q | Q |  |  |  |  |  |  |  |  |
| Have you visited the practice of your GP in the last six months? (no/yes) | self-developed |  |  | Q | Q |  |  |  |  |  |  |  |  |
| Family involvement in medical care |  |  |  |  |  |  |  |  |  |  |  |  |  |
| How often does it happen that your relatives take care of your medical care, e.g. by arranging appointments with the GP or GP’s visits at your nursing home? (1=regularly … 4=cannot say exactly) | Koverdem (adapted) |  |  | Q | Q |  |  |  |  |  |  |  |  |

*According to the German social long-term care insurance, levels 0 to 5, with higher levels representing greater care needs.

Abbreviations: *GP,* general practitioner; *IPAV, interprof* ACT agent*; M,* minutes; *NH,* nursing home; *NHD,* nursing home director; *NHR,* nursing home resident; *Q,* questionnaire; *RN,* registered nurse; *STM,* study team member*; T0a,* baseline assessment (before randomized allocation); *T1,* follow-up after 6 months; *T2,* follow-up after 12 months.

Instruments: *Koverdem* Survey instruments of the research project “Optimizing Cooperation between General Practitioners and Home Care Services” [7]; *ZAP* Zufriedenheit in der Arztpraxis (“Satisfaction in GPs Office”) [11].

### Qualitative strand

**Table 2.6** Themes of interview or observation guidelines for the five outcome domains

| **Themes of interview/observation guidelines** | **Target population at specific measurement points** | | | | | |
| --- | --- | --- | --- | --- | --- | --- |
|  | **GP** | | | **RN** | | |
|  | **T0b** | **T1** | **T2** | **T0b** | **T1** | **T2** |
| **Implementation strategies and activities** |  |  |  |  |  |  |
| Implementation of *interprof* ACT measures, barriers and facilitators (only IG) | I, O | I, O | I, O | I, O | I, O | I, O |
| **Implementation of *interprof* ACT components** |  |  |  |  |  |  |
| Feasibility of *interprof* ACT measures (only IG) | I, O | I, O | I, O | I, O | I, O | I, O |
| Tailoring of *interprof* ACT measures during kick-off meeting (only IG) | O |  |  | O |  |  |
| Process changes through *interprof* ACT intervention (only IG) | I, O | I, O | I, O | I, O | I, O | I, O |
| **Key elements of interprofessional collaboration** |  |  |  |  |  |  |
| Perceptions of work processes between RNs and GPs | I | I | I | I | I | I |
| Communication and interaction between participants during kick-off meeting (only IG) | O |  |  | O |  |  |
| Communication and interaction between RNs and GPs in everyday work | O | O | O | O | O | O |
| **Further domains related to interprofessional collaboration and medical care** |  |  |  |  |  |  |
| Process sequences of care | I, O | I, O | I, O | I, O | I, O | I, O |
| Process sequences of interprofessional collaboration | I, O | I, O | I, O | I, O | I, O | I, O |
| Process sequences of hospital admissions | I, O | I, O | I, O | I, O | I, O | I, O |
| **Context factors** |  |  |  |  |  |  |
| Everyday work context of RNs and GPs | I | I | I | I | I | I |
| Barriers and facilitators of process performance | I | I | I | I | I | I |

Abbreviations: *GP,* general practitioner; *I,* interview; *IG,* intervention group; *O,* observation; *RN,* registered nurse; *T0b,* shortly post randomization; *T1,* follow-up after 6 months; *T2,* follow-up after 12 months.

## Data analysis

### Detailed description of the data analysis for quantitative process evaluation

The following steps guided the quantitative data analysis at the cluster (nursing home) level for the outcome domains: (1) implementation of *interprof* ACT components, (2) key elements of interprofessional collaboration, (3) further domains related to interprofessional collaboration and medical care, and (4) context factors.

1. Assessment of pre-post changes (T0a or T1 versus T2) in the single items or sum scores, respectively, for each subdomain and dimension of the outcome domain of interest (Table 2.7), separately for each perspective (nursing home directors, nursing home residents, general practitioners, registered nurses, and *interprof* ACT agents) (all four outcome domains).
2. Aggregation of item- or sum score-specific change estimates to summary change estimates for each subdomain or dimension, separately for each perspective (vote counting) (all four outcome domains).
3. Generation of global performance indicators for outcome domains, separately for each perspective (only domains (1) implementation of *interprof* ACT components, (2) key elements of interprofessional collaboration).
4. Determination of the mean ±360° global performance indicator (only domain (1) implementation of *interprof* ACT components).

**Table 2.7** Examples of outcome domains, subdomains, and dimensions (all domains are displayed in Table 3 in the main manuscript or in the study protocol [1])

| **Domains** | **Subdomains** | **Dimensions** |
| --- | --- | --- |
| **Implementation strategies and activities** | | |
| Implementational work within a team (normalization process theory) | Coherence, cognitive participation, collective action, reflexive monitoring |  |
| ***interprof* ACT intervention package** | | |
| Name badges |  | Choice and adaptation, dose and reach, local policies, current practice |
| Mandatory availability rules |  | Choice and adaptation, dose and reach, attitudes, current practice, quality and satisfaction |
| Designated contact persons |  | Choice and adaptation, dose and reach, attitudes, local policies, current practice, quality and satisfaction |
| Standardized GPs’ home visits |  | Choice and adaptation, dose and reach, attitudes, current practice, quality and satisfaction |
| Pro re nata medication |  | Choice and adaptation, dose and reach, current practice, quality and satisfaction |
| Shared goal setting |  | Choice and adaptation, dose and reach, attitudes, current practice, quality and satisfaction |

Abbreviations: *GP,* general practitioner.

1. **Assessment of pre-post changes per perspective and outcome subdomains/dimensions**

First, we assessed the size of the pre-post changes (T0a or T1 versus T2) or the size of subjectively perceived changes (T2 measurements) in the single items or sum scores for each outcome subdomain or dimension (Table 2.8). We estimated these changes at the cluster level for each of the following perspectives:

- Nursing home directors
- Nursing home residents
- General practitioners: Questionnaire with two parts (Part A: general, Part B: nursing home-specific)
- Registered nurses: Questionnaire with two parts (Part A: general, Part B: general practitioner-specific)
- *interprof* ACT agents

This step of aggregation was carried out for each of the following outcome domains: (1) implementation of *interprof* ACT components, (2) key elements of interprofessional collaboration, (3) further domains related to interprofessional collaboration and medical care, and (4) context factors.

**Table 2.8** Assessment of pre-post changes

| **Estimation of changes based on pre- and post- measurements (T0a/T1–T2)** |  | **Estimation of changes based on subjective ratings  at T2 only** |
| --- | --- | --- |
| For each cluster, the pre-post differences in the median for each item or sum score, respectively, was calculated. The size of the difference was classified as the following:   - Relevant change if median difference was 0.5 in case ≤5-step scales or 1.0 in case of >5-step scales - Indifferent change in all other cases |  | For each cluster, the distance between the item’s median value and the most neutral value (reflecting no changes in either direction) of the item scale was calculated. The size of the difference was classified as the following:   - Relevant change if the distance was ≥0.5 in case ≤5-step scales or ≥1.0 in case of >5-step scales - Indifferent change in all other cases |
|  | | |
| **Classification of change directions per item:**  Positive change = relevant change in a favorable direction  Negative change = relevant change in an unfavorable direction No change = indifferent change | | |

Abbreviations: *T0a* baseline assessment (before randomized allocation); *T1* follow-up after 6 months; *T2* follow-up after 12 months.

1. **Aggregation of item- or sum score-specific change estimates to summary change estimates for each subdomain or dimension (vote counting)**

In a three-step approach, we then summarized the item- or sum score-based change estimates into aggregated change measures for each subdomain/dimension, again separately for each cluster. Table 2.9 provides a detailed overview of these aggregation procedures. First, by means of vote counting, we aggregated the item-specific change estimates to overall change estimates per subdomain/dimension for each perspective and cluster, displayed by arrows. Second, we translated the arrow-type change measure into a ±360° summary change measure per cluster for each subdomain or dimension. Except for some change measures retrieved from the registered nurses’ perspective, one arrow-type change measure equals one ±360° summary change measure. Since registered nurses rated several general practitioners for some subdomains/dimensions, multiple arrow-type change measures, each related to a specific practitioner, were retrieved from their perspective for some subdomains/dimensions and had to be combined into one ±360° summary change measure, together with general practitioner-unrelated change measures. Finally, in the third step, uncertainties in the ±360° summary change measures due to missing values were assessed and flagged out.

This three-step aggregation was carried out for each of the following outcome domains: (1) implementation of *interprof* ACT components, (2) key elements of interprofessional collaboration, (3) further domains related to interprofessional collaboration and medical care, and (4) context factors.

**Table 2.9** Three steps of aggregation of change estimates to ±360° summary change measures

| **Step 1: Aggregation of changes per subdomain or dimension for each perspective for each cluster** | | | | | | | |
| --- | --- | --- | --- | --- | --- | --- | --- |
| ↑ | All items for one subdomain/dimension show a positive change | | | | | | |
| 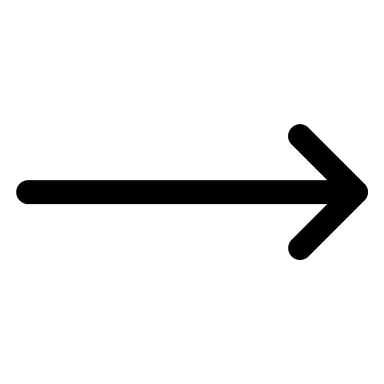 | Most of the items for one subdomain/dimension show a positive change (some items show no change) | | | | | | |
| ↔ | All items for one subdomain/dimension show no change | | | | | | |
| 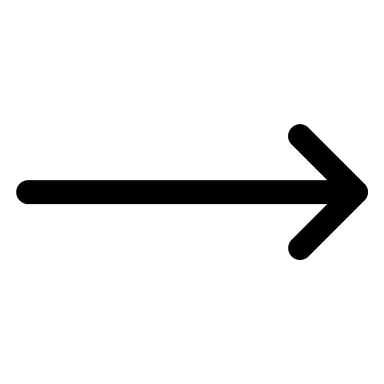 | Most of the items for one subdomain/dimension show a negative change (some items show no change) | | | | | | |
| ↓ | All items for one subdomain/dimension show a negative change | | | | | | |
| 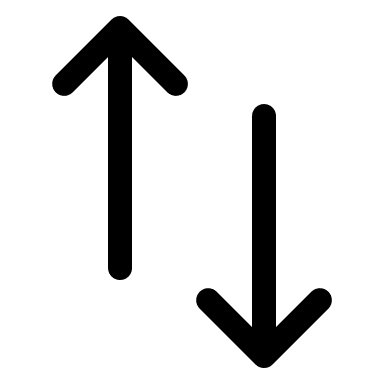 | Indifferent change (some items for one subdomain/dimension show a positive change while other items show a negative or no change) | | | | | | |
| n.d. | Not displayable change for one subdomain/dimension due to missing data | | | | | | |
| **Nursing home director** | | **Nursing home resident** | | **General practitioner** | **Registered nurse** | | ***interprof* ACT agent** |
| One arrow per subdomain/dimension | | One arrow per subdomain/dimension | | One arrow per subdomain/dimension (some unrelated to a specific nursing home, some related to one specific nursing home) | **Part A questionnaire (unrelated to a specific general practitioner):**  One arrow per subdomain/dimension  **Part B questionnaire (related to several specific general practitioners):**  Multiple arrows per subdomain/dimension (each arrow specific to one practitioner) | | One arrow per subdomain/dimension |
| **Step 2: Translation into a ±360° summary change measure per subdomain or dimension for each cluster** | | | | | | | |
| ↑ | | | 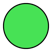 | | | 360° | |
| 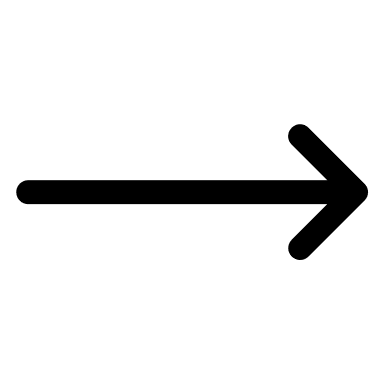 | | | 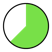 | | | 225° | |
| ↔,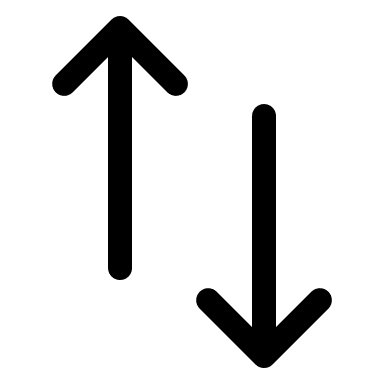 | | | 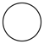 | | | 0° | |
| 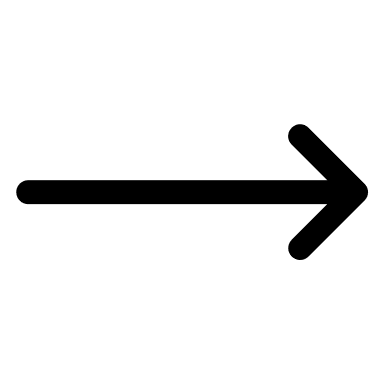 | | | 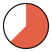 | | | -225° | |
| ↓ | | | 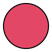 | | | -360° | |
| n.d. | | | 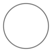 | | | (0°) | |
| **Nursing home director** | | **Nursing home resident** | | **General practitioner** | **Registered nurse** | | ***interprof* ACT agent** |
| One ±360° summary change measure per subdomain/dimension | | One ±360° summary change measure per subdomain/dimension | | One ±360° summary change measure per subdomain/dimension  (some unrelated to a specific nursing home, some related to one specific nursing home) | **For some subdomains/ dimensions:**  One ±360° summary change measure per subdomain/dimension  **For some subdomains/ dimensions:**  Multiple GP-specific and one GP-unspecific arrow-type change measure were first translated into single ±360° summary change measures that were then summarized into one ±360° summary change measure per subdomain/dimension with the following weighting:  60% GP-specific change measures and 40% GP-unspecific change measures | | One degree per subdomain/dimension |
| **Step 3: Assessment of the uncertainty of the ±360° summary change measures** | | | | | | | |
| The measure “certainty” represents the proportion of available data on the degree of changes from the different perspectives. In cases where summary change estimates were not displayable (n.d.) due to missing data in more than 50% of items, the summary change estimates were highlighted as “uncertain”. | | | | | | | |

Abbreviations: *GP,* general practitioner; *n.d.,* not displayable.

1. **Generation of global performance indicators for outcome domains**

For the outcome domains “implementation of *interprof* ACT components” and “key elements of interprofessional collaboration”, the ±360° summary change measures retrieved at step 2 for each subdomain/dimension were added up to a domain-specific ±360° “global performance indicator”, reflecting the reach, dose and fidelity of the implementation of *interprof* ACT components or the overall direction and size of changes in interprofessional collaboration from each party’s perspective (Table 2.10).

**Table 2.10** Development of the ±360° global performance indicator for the domains “implementation of *interprof* ACT components” and “key elements of interprofessional collaboration”

| **Step 1: Aggregation of the summary change estimates for multiple subdomains/dimensions in a domain-specific ±360° “global performance indicator” for each cluster and separately for each perspective** |
| --- |
| Each subdomain/dimension was counted with equal weight in the global performance indicator.  **Example for the development of the global performance indicator for the domain “standardized GPs’ home visit”:**   \| **Perspective** \| **General practitioner** \| \| \| --- \| --- \| --- \| \| **Dimensions** \| **Quality and satisfaction** \| **Current practice** \| \| **±360° summary change estimates** \| 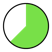  225° \| 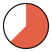  -225° \| \| **= ±360° global performance indicator** \| 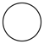  (225°x0.5) + (-225°x0.5) = 0° \| \| |
| **Step 2: Determination of the certainty/uncertainty of the global performance indicator estimate** |
| In cases where the ±360° summary change measures were prone to uncertainty (see Table 2.9), the global performance indicator was highlighted as “uncertain”. |

1. **Determination of the cluster-specific mean ±360° global performance indicator for the implementation of each *interprof* ACT component**

Finally, we summarized the ±360° global performance indicator values retrieved from each perspective for the outcome domain “implementation of the *interprof* ACT components” into one overall cluster-specific ±360° global performance indicator across the rater perspectives representing the overall success of implementation in the respective nursing home; this was done by determining the mean value of all rater-specific ±360° global performance indicator estimates for the outcome domain “implementation of the *interprof* ACT components”, separately for each intervention component, i.e., name badges, mandatory availability rules, designated contact persons, standardized GPs’ home visits, pro re nata medication, and shared goal setting.

# Results

## Response rates and characteristics of populations

### Response rates

**Table 2.11** Overview of the number of questionnaires and response rates

|  | **Nursing home directors** | | **Nursing home residents** | | **General practitioners** | | **Registered nurses** | | ***interprof* ACT agents** |
| --- | --- | --- | --- | --- | --- | --- | --- | --- | --- |
|  | **IG** | **CG** | **IG** | **CG** | **IG** | **CG** | **IG** | **CG** | **IG** |
| Total number (included in the main trial) | 17 | 17 | 320 | 323 | 123 | 110 | - | - | - |
| Number of individuals participating in the process evaluation | 17 | 17 | 191* | 202* | 65 | 64 | - | - | - |
| **T0a** |  |  |  |  |  |  |  |  |  |
| Number of questionnaires handed out | 17 | 17 | 191 | 202 | 65 | 64 | 101** | 126 | - |
| Number of questionnaires returned | 16 | 17 | 166*** | 157*** | 57 | 50 | 73 | 78 | - |
| Number of questionnaires included in analysis | 16 | 17 | 166 | 157 | 57 | 50 | 72 | 72 |  |
| Response rate (%) | 94.1 | 100.0 | 86.9 | 77.7 | 87.7 | 78.1 | 72.3 | 61.9 |  |
| **T1** |  |  |  |  |  |  |  |  |  |
| Number of questionnaires handed out | - | - | - | - | - | - | - | - | 25 |
| Number of questionnaires returned | - | - | - | - | - | - | - | - | 19 |
| Number of questionnaires included in analysis | - | - | - | - | - | - | - | - | 19 |
| Response rate (%) | - | - | - | - | - | - | - | - | 76.0 |
| **T2** |  |  |  |  |  |  |  |  |  |
| Number of questionnaires handed out | 17 | 17 | 111 | 129 | 62 | 55 | 102 | 110 | 28 |
| Number of questionnaires returned | 16 | 15 | 94*** | 96*** | 50 | 42 | 70 | 63 | 24 |
| Number of questionnaires included in analysis | 16 | 15 | 94 | 96 | 50 | 42 | 70 | 62 | 24 |
| Response rate (%) | 94.1 | 88.2 | 84.7 | 74.4 | 80.6 | 76.4 | 68.6 | 57.3 | 85.7 |

*Only nursing home residents with Dementia Screening Scale scores ≤4. **Missing number of questionnaires handed out (n=1 nursing home). ***The reason for missing questionnaires from nursing home residents are explained in Table 2.12.

Abbreviations: *CG,* control group; *IG,* intervention group; *T0a,* baseline assessment (before randomized allocation); *T1,* follow-up after 6 months; *T2,* follow-up after 12 months.

**Table 2.12** Reasons for missing questionnaires

| **Reasons** | **T0a** | | **T2** | |
| --- | --- | --- | --- | --- |
|  | **IG (n=25)** | **CG (n=45)** | **IG (n=17)** | **CG (n=33)** |
| NHR in hospital | 2 | - | - | - |
| NHR rejected interview participation | 4 | 3 | 7 | 7 |
| NHR not able to rate care provided by GP | 3 | 9 | - | 1 |
| NHR not present in the NH at interview time point | - | 2 | - | - |
| NHR not able to answer questions (e.g. due to sleeping) | - | 2 | - | 3 |
| Closure of the NH | - | - | - | 7 |
| No information | 16 | 29 | 10 | 15 |

Abbreviations: *CG,* control group; *GP,* general practitioner; *IG,* intervention group; *NH,* nursing home; *NHR,* nursing home resident; *T0a,* baseline assessment (before randomized allocation); *T2,* 12 months post randomization.

### Sample description: Nursing homes

**Table 2.13** Characteristics of the nursing homes

|  | **Quantitative process evaluation** | | **Qualitative process evaluation** | |
| --- | --- | --- | --- | --- |
|  | Intervention group | Control group | Intervention group | Control group |
| **Nursing homes (Baseline)** |  |  |  |  |
| Number of homes | 17 | 17 | 5 | 5 |
| Number of responses from NHDs | 16 | 17 | 5 | 5 |
| Ownership (n [valid %]) |  |  |  |  |
| Private | 8 [50.0] | 13 [76.5] | 1 [20.0] | 5 [100.0] |
| Not-for-profit | 7 [44.8] | 3 [17.6] | 3 [60.0] | 0 [0.0] |
| Public and ecclesiastical | 1 [6.3] | 1 [5.9] | 1 [20.0] | 0 [0.0] |
| *Missing* | *-* | *-* | *-* | *-* |
| Number of places in long-term care (median [IQR], min–max)  *Missing* | 93.5 [61.6–114.8],  42.0–255.0  *-* | 96.0 [65.0–122.5],  41.0–162.0  - | 92.0 [63.0–157.5],  60.0–220  *-* | 110.0 [57.0–123.5],  41.0–132.0  *-* |
| Number of places occupied in long-term care (median [IQR], min–max)  *Missing* | 91 [60.8–99],  39.0–211.0  *-* | 79.0 [58.0–116.0],  0.0–151.0  - | 90.0 [62.5–137.5]  59.0–183.0  *-* | 57.0 [20.5–107.0],  0.0–129.0  *-* |
| Number of registered nurses (fulltime equivalent) (median [IQR], min–max)  *Missing* | 11.3 [9.9–18.3],  5.0–42.0  *2* | 16.0 [9.0–22.0],  5.0–24.5  *1* | 11.5 [10.7–20.0],  10.3–28.0  *-* | 12.0 [7.1–21.3],  6.9–23.1  *1* |
| RN-NHR ratio (median [IQR], min–max)  *Missing* | 0.2 [0.1–0.2],  0.1–0.3  *2* | 0.2 [0.2–0.2],  0.1–0.3  *2* | 0.2 [0.1–0.2],  0.1–0.2  *-* | 0.2 [0.1–0.2],  0.1–0.2  *2* |
| Proportion [%] of NHRs with care level 3 and higher (median [IQR], min–max)  *Missing* | 75.3 [65.6–81.4]  56.6–100.0  *2* | 71.8 [64.7–76.1]  45.6–95.0  *-* | 76.7 [68.1–80.9]  65.6–81.4  *-* | 63.7 [50.5–79.6]  45.6–83.6  *-* |
| Proportion [%] of NHRs with care level 4 and higher (median [IQR], min–max)  *Missing* | 42.3 [38.3–51.7]  28.3–87.5  *2* | 37.9 [30.4–42.2]  15.8–63.3  *-* | 39.4 [38.8–52.1]  38.3–55.9  *-* | 32.4 [20.5–45.3]  15.8–51.2  *-* |
| Size of nursing home location <100.000 inhabitants (n [valid %])  *Missing* | 8 [50.0]  *-* | 8 [47.1]  - | 3 [60.0]  *-* | 3 [60.0]  *-* |
| Number of wards (median [IQR],  min–max)  *Missing* | 3.0 [2.0–4.8],  1.0–8.0  *-* | 3.0 [2.0–3.8],  1.0–6.0  *1* | 2.0 [2.0–6.0],  2.0–8.0  *-* | 2.0 [1.5–3.5],  1.0–4.0  *-* |
| Cooperation agreements (multiple entries possible) (n [valid %]) |  |  |  |  |
| General practitioner according to §119b Abs. 2 SGBV | 5 [31.3] | 6 [35.3] | 2 [40.0] | 1 [20.0] |
| General practitioner-centered care according to §73b SGBV | 0 [0] | 2 [11.8] | 0 [0.0] | 1 [20.0] |
| Special medical care according to §140a SGBV | 0 [0] | 0 [0] | 0 [0.0] | 0 [0.0] |
| 24-hour availability of general practitioners every day of the week | 2 [12.5] | 0 [0] | 1 [20.0] | 0 [0.0] |
| No existing cooperation agreements | 3 [18.8] | 4 [23.5] | 0 [0.0] | 3 [60.0] |
| Others | 4 [25.0] | 7 [41.2] | 1 [20.0] | 1 [20.0] |
| *Missing* | - | - | *-* | *-* |
| Number of all general practitioners involved in medical care of NHRs (median [IQR], min–max)  *Missing* | 12.0 [5.0–20.0],  0.0–22.0  *1* | 10.0 [5.0–16.0],  1.0–18.0  *2* | 20.0 [16.5–21.5],  16.0–22.0  *-* | 6.5 [1.5–16.0],  1.0–18.0  *1* |
| Number of all GPs from participating NHRs per NH (median [IQR], min–max)  *Missing* | 7.0 [5.0–9.0],  3.0–12.0  *-* | 6.0 [5.0–8.0],  1.0–12.0  *-* | 9.0 [6.0–10.0],  5.0–11.0  *-* | 6.0 [5.0–8.0],  1.0–9.0  *-* |
| Number of participating GPs (with completed questionnaire T0a) from participating NHRs per NH (median [IQR], min–max)  *Missing* | 3.0 [2.0–4.0],  0.0–7.0  *-* | 3.0 [2.0–4.0],  1.0–6.0  *-* | 3.0 [3.0–4.0],  2.0–5.0  *-* | 2.0 [2.0–3.0],  1.0–3.0  *-* |
| Proportion of all NHRs per NH cared for by participating GP (median [IQR], min–max)  *Missing* | 11.6 [7.2–20.0]  1.3–45.0  3 | 16.7 [6.0–26.0]  2.5–40.6  1 | 9.2 [5.2–13.1]  1.7–16.1  - | 18.0 [6.3–22.6]  3.5–23.1  *1* |
| **Nursing homes (T2 measuring point)** |  |  |  |  |
| Number of homes | 17 | 17 | 5 | 5 |
| Number of responses from NHDs | 16 | 15 | 5 | 4 |
| Ownership (n [valid %]) |  |  |  |  |
| Private | 7 [43.8] | 11 [73.3] | 1 [20.0] | 4 [100.0] |
| Not-for-profit | 8 [50.0] | 2 [13.3] | 4 [80.0] | 0 [0.0] |
| Public and ecclesiastical | 1 [6.3] | 1 [6.7] | 0 [0.0] | 0 [0.0] |
| Others | 0 [0.0] | 1 [6.7\| | 0 [0.0] | 0 [0.0] |
| *Missing* | *-* | *-* | *-* | *-* |
| Number of places in long-term care (median [IQR], min–max)  *Missing* | 98.5 [62.3–135.3],  42.0–256.0  *-* | 80.0 [60.0–124.0],  41.0–162.0  *-* | 100 [64.5–165.0],  60.0–220.0  *-* | 87.0 [45.5–127.8],  41.0–132.0  *-* |
| Number of places occupied in long-term care (median [IQR], min–max)  *Missing* | 92.0 [60.0–101.0],  41.0–251.0  *1* | 72.0 [55.0–123.0],  41.0–147.0  *-* | 83.0 [61.8–129.8],  60.0–140.0  *1* | 65.0 [44.0–115.3],  41.0–128.0  *-* |
| Number of registered nurses (fulltime equivalent) (median [IQR], min–max)  *Missing* | 18.0 [12.3–21.4],  7.4–46.0  *1* | 11.9 [8.9–17.8],  4.0–23.8  *1* | 19.1 [14.0–26.40],  12.3–28.8  *1* | 9.1 [8.2–23.8],  8.2–23.8  *1* |
| RN-NHR ratio (median [IQR], min–max)  *Missing* | 0.2 [0.2–0.2],  0.1–0.3  *2* | 0.2 [0.1–0.2],  0.1–0.2  *1* | 0.2 [0.2–0.2],  0.2–0.2  *2* | 0.2 [0.1–0.2],  0.1–0.2  *1* |
| Proportion [%] of NHRs with care level 3 and higher (median [IQR], min–max)  *Missing* | 78.8 [73.4–83.1]  67.1–100.0  *4* | 69.1 [62.9–74.8]  56.6–91.1  *2* | 78.8 [73.7–90.0]  73.7–90.0  *2* | 67.0 [57.1–79.0]  56.6–80.2  *-* |
| Proportion [%] of NHRs with care level 4 and higher (median [IQR], min–max)  *Missing* | 47.4 [38.5–55.1]  25.5–86.4  *4* | 34.5 [30.4–37.9]  15.6–67.9  *2* | 47.4 [38.4–73.3]  38.4–73.3  *2* | 33.4 [15.6–41.2]  19.2–40.1  *-* |
| Number of wards (median [IQR], min–max)  *Missing* | 3.0 [2.0–5.0],  1.0–8.0  *-* | 3.0 [2.0–4.0],  1.0–6.0  *-* | 2.0 [2.0–4.5],  2.0–6.0  *-* | 4.0 [1.5–5.8],  1.0–6.0  *-* |
| Cooperation agreements (multiple entries possible) (n [valid %]) |  |  |  |  |
| General practitioner according to §119b Abs. 2 SGBV | 4 [25.0] | 6 [40.0] | 1 [20.0] | 2 [50.0] |
| General practitioner-centered care according to §73b SGBV | 2 [12.5] | 5 [33.3] | 1 [20.0] | 2 [50.0] |
| Special medical care according to §140a SGBV | 1 [6.3] | 0 [0.0] | 0 [0.0] | 0 [0.0] |
| 24-hour availability of general practitioners every day of the week | 2 [12.5] | 1 [6.7] | 1 [20.0] | 0 [0.0] |
| No existing cooperation agreements | 6 [37.5] | 1 [6.7] | 1 [20.0] | 0 [0.0] |
| Others | 3 [18.8] | 4 [26.7] | 1 [20.0] | 2 [50.5] |
| *Missing* | *-* | *-* | *-* | *-* |
| Number of all general practitioners involved in medical care of NHRs (median [IQR], min–max)  *Missing* | 9.0 [5.0–15.9],  0.0–26.0  *1* | 7.0 [5.0–12.0],  2.0–20.0  *-* | 19.0 [13.0–24.3],  11.0–26.0  *1* | 4.5 [2.3–6.8],  2.0–7.0  *-* |
| Quality inspections by the Medical Review Board of the Statutory Health Insurance Funds (MD) (n [valid %])  *Missing* | 14 [93.3]  *1* | 14 [93.3]  *-* | 5 [100.0]  *-* | 4 [100.0]  *-* |
| Structural changes, yes (n [valid %])  *Missing* | 9 [60.0]  *1* | 5 [33.3]  *-* | 3 [60.0]  *-* | 2 [50.0]  *-* |

Abbreviations: *GP,* general practitioner; *IQR,* interquartile range; *max,* maximum; *min,* minimum; *NH,* nursing home; *NHD,* nursing home director; *NHR,* nursing home resident; *RN,* registered nurse; *T0a,* baseline assessment (before randomized allocation); *T2,* 12 months post randomization.

### Sample description: Nursing home residents

**Table 2.14** Characteristics of the nursing home residents

|  | **Quantitative process evaluation** | | **Qualitative process evaluation** | |
| --- | --- | --- | --- | --- |
|  | Intervention group | Control group | Intervention group | Control group |
| **Nursing home residents (Baseline)** |  |  |  |  |
| Number of participating NHRs in process evaluation (DSS≤4) | 166 | 157 | 48 | 43 |
| Support of relatives for medical care, regular (N [valid %])  *Missing* | 33 [21.4]  *12* | 27 [19.0]  *15* | 12 [27.9]  *5* | 6 [15.8]  *5* |
| Visits of NHRs at GPs office (within last 6 months), yes (N [valid %])  *Missing* | 35 [21.7]  *5* | 47 [30.3]  *2* | 10 [21.7]  *2* | 11 [25.6]  *-* |
| Medical care by current GP in recent 1–2 years (N [valid %])  *Missing* | 43 [27.6]  *10* | 33 [22.3]  *9* | 10 [22.2]  *3* | 14 [34.1]  *2* |
| **Nursing home residents (T2 measuring point)** |  |  |  |  |
| Number of participating NHRs in process evaluation (DSS≤4) | 94 | 96 | 34 | 21 |
| Support of relatives for medical care, regular (N [valid %])  *Missing* | 15 [16.9]  *5* | 18 [20.0]  *6* | 4 [12.1]  *1* | 2 [9.5]  *-* |
| Visits of NHRs at GPs office (within last 6 months), yes (N [valid %])  *Missing* | 21 [22.3]  *-* | 27 [28.1]  *-* | 4 [11.8]  *-* | 3 [14.3]  *-* |
| Medical care by current GP in recent 1–2 years (N [valid %])  *Missing* | 24 [26.1]  *2* | 26 [28.6]  *5* | 7 [21.2]  *1* | 6 [33.3]  *3* |

Abbreviations: *DSS,* Dementia Screening Scale [13]; *GP,* general practitioner; *NHR,* nursing home resident; *T2,* 12 months post randomization.

### Sample description: General practitioners’ offices

**Table 2.15** Characteristics of general practitioners’ offices

|  | **Quantitative process evaluation** | | **Qualitative process evaluation** | |
| --- | --- | --- | --- | --- |
|  | Intervention group | Control group | Intervention group | Control group |
| **General Practitioners‘ offices (Baseline)** |  |  |  |  |
| Size of practice location <100.000 inhabitants (n [valid %])  *Missing* | 18 [32.7]  *-* | 19 [40.4]  *-* | 10 [55.5]  *-* | 6 [54.6]  *-* |
| Academic teaching practice, yes (n [valid %])  *Missing* | 22 [40.7]  *1* | 17 [36.2]  *-* | 6 [33.3]  *-* | 3 [27.3]  *-* |
| Cooperation agreements (multiple entries possible) (n [valid %]) |  |  |  |  |
| General practitioner according to §119b Abs. 2 SGBV | 8 [14.5] | 10 [21.7] | 3 [17.6] | 3 [27.3] |
| General practitioner-centered care according to §73b SGBV | 13 [23.6] | 7 [15.2] | 4 [23.5] | 3 [27.3] |
| Special medical care according to §140a SGBV | 2 [3.6] | 0 [0] | 1 5.9] | 0 [0.0] |
| 24-hour availability of general practitioners every day of the week | 3 [5.5] | 1 [2.2] | 1 [5.9] | 0 [0.0] |
| No existing cooperation agreements | 34 [61.8] | 28 [60.9] | 9 [52.9] | 6 [54.5] |
| Others | 2 [3.6] | 4 [8.0] | 2 [11.8] | 0 [0.0] |
| *Missing* | *2* | *4* | *-* | *-* |
| Medical assistants with additional qualifications, yes (n [valid %])  *Missing* | 28 [50.9]  *-* | 28 [59.6]  *-* | 11 [61.1]  *-* | 7 [63.6]  *-* |
| **General Practitioners‘ offices (T2 measuring point)** |  |  |  |  |
| Academic teaching practice, yes (n [valid %])  *Missing* | 19 [44.2]  - | 10 [28.6]  - | 4 [33.3]  - | 1 [11.1] |
| Cooperation agreements (multiple entries possible) (n [valid %]) |  |  |  |  |
| General practitioner according to §119b Abs. 2 SGBV | 7 [17.7] | 4 [11.8] | 2 [18.2] | 4 [44.4] |
| General practitioner-centered care according to §73b SGBV | 9 [22.0] | 5 [14.7] | 3 [27.3] | 2 [22.2] |
| Special medical care according to §140a SGBV | 2 [4.9] | 0 [0.0] | 1 [9.1] | 1 [11.1] |
| 24-hour availability of general practitioners every day of the week | 3 [7.3] | 0 [0.0] | 1 [9.1] | 0 [0.0] |
| No existing cooperation agreements | 25 [61.0] | 23 [67.6] | 5 [45.5] | 4 [44.4] |
| Others | 2 [0.5] | 4 [11.8] | 2 [18.1] | 0 [0.0] |
| *Missing* | *9* | *5* | *5* | *-* |
| Medical assistants with additional qualifications, yes (n [valid %])  *Missing* | 20 [46.5]  - | 21 [60.0]  - | 6 [50.0]  - | 6 [66.7]  - |

Abbreviations: *T2,* 12 months postrandomization.

### Sample description: General practitioners

**Table 2.16** Characteristics of general practitioners

|  | **Quantitative process evaluation** | | **Qualitative process evaluation** | |
| --- | --- | --- | --- | --- |
|  | Intervention group | Control group | Intervention group | Control group |
| **General Practitioners (Baseline)** |  |  |  |  |
| Number | 55 | 47 | 18 | 11 |
| Age (median [IQR], min–max)  *Missing* | 56.0 [50.0–60.0],  35.0–76.0  *-* | 54.0 [47.7–60.0],  34.0–68.0  *1* | 57.5 [51.5–60.5],  44.0–65.0  *-* | 49.0 [43.0–61.0],  34.0–68.0  *-* |
| Sex, female (n [valid %])  *Missing* | 20 [36.4]  *-* | 16 [34.8]  *1* | 6 [33.3]  *-* | 6 [54.5]  *-* |
| Practice ownership (n [valid %]) |  |  |  |  |
| No | 7 [12.7] | 4 [8.5] | 1 [5.6] | 1 [9.1] |
| Yes, single | 17 [30.9] | 23 [48.9] | 8 [44.4] | 8 [72.7] |
| Yes, together with co-owners or employed GPs | 31 [56.4] | 20 [42.6] | 9 [50.0] | 2 [18.2] |
| *Missing* | *-* | *-* | *-* | *-* |
| Medical specialist education as GP completed, yes (n [valid %])  *Missing* | 35 [63.6]  *-* | 27 [57.4]  *-* | 10 [55.6]  *-* | 8 [72.7]  *-* |
| Years of experience as physician (median [IQR], min–max)  *Missing* | 26.0 [22.0–32.0],  7.5–50.0  *-* | 26.0 [18.0–32.0],  7.0–32.0  *2* | 28.0 [24.5–32.5],  17.0–36.0  *-* | 21.0 [17.0–34.0],  7.0–39.0  *-* |
| Years of experience as GP (median [IQR], min–max)  *Missing* | 16.0 [10.0–25.0],  1.0–40.0  *-* | 14.7 [8.5–24.0],  2.5–33.0  *1* | 19.5 [13.3–23.5],  2.5–32.0  *-* | 12.0 [5.0–27.0],  2.5–33.0  *-* |
| Years of experience with medical care for NHRs (median [IQR],  min–max)  *Missing* | 15.0 [10.0–23.0],  1.0–40.0  *-* | 14.0 [7.0–23.0],  2.5–33.0  *-* | 19.5 [11.0–23.3],  2.5–31.0  *-* | 10.0 [4.0–23.0],  2.5–33.0  *-* |
| Number of NHs with NHRs cared for (median [IQR], min–max)  *Missing* | 4.0 [3.0–6.0],  1.0–17.0  *1* | 4.0 [2.5–6.0],  1.0–17.0  *-* | 6.0 [4.0–7.0],  3.0–10.0  *1* | 5.0 [2.0–8.0],  1.0–13.0  *-* |
| Number of NHRs in participating NH cared for by this GP (median [IQR], min–max)  *Missing* | 10.0 [2.5–35.5],  1.0–110.0  *2* | 10.0 [3.0–21.0],  0.0–100.0  *-* | 5.5 [3.0–23.0],  1.0–70.0  2 | 5.5 [2.0–23.0],  0.0–42.0  *1* |
| Estimated proportion (%) of NHRs among total number of patients cared for (median [IQR], min–max)  *Missing* | 5.0 [2.8–9.3],  0.5–33.0  *2* | 5.0 [2.5–10.0],  0.5–20.0  *2* | 6.5 [4.8–10.0],  1.0–15.0  *-* | 5.0 [2.0–10.0],  0.5–15.0  *-* |
| Estimated hours per week required for medical care for NHRs (median [IQR], min–max)  *Missing* | 5.2 [3.0–7.9],  0.5–36.6  *1* | 5.0 [2.9–7.6],  0.0–15.0  *1* | 5.0 [3.0–7.9],  2.0–15.7  *-* | 5.0 [2.0–10.0],  0.0–15.0  *-* |
| Delegation of NH visits to medical assistants, yes (n [valid %])  *Missing* | 37 [67.3]  *-* | 35 [74.5]  *-* | 13 [72.2]  *-* | 7 [63.6]  *-* |
| **General Practitioners (T2 measuring point)** |  |  |  |  |
| Number | 43 | 35 | 12 | 9 |
| Practice ownership (n [valid %]) |  |  |  |  |
| No | 5 [11.6] | 2 [5.7] | 0 [0.0] | 0 [0.0] |
| Yes, single | 14 [32.6] | 20 [57.1] | 6 [50.0] | 8 [88.9] |
| Yes, together with co-owners or employed GPs | 24 [55.8] | 13 [37.1] | 6 [50.0] | 1 [11.1] |
| *Missing* | *-* | *-* | *-* | *-* |
| Number of NHs with NHRs cared for (median [IQR], min–max)  *Missing* | 4.0 [3.0–6.0],  1.0–17.0  *1* | 4.0 [3.0–6.0],  1.0–17.0  *-* | 7.0 [3.0–7.0],  3.0–9.0  *1* | 5.0 [2.0–7.0],  1.0–13.0  *-* |
| Number of NHRs in participating NH cared for by this GP (median [IQR], min–max)  *Missing* | 6.5 [2.3–24.8],  1.0–90.0  *-* | 6.0 [3.0–25.8],  0.0–100.0  *-* | 3.5 [2.0–14.8],  1.0–60.0  - | 5.0 [3.0–50.0],  0.0–54.0  *2* |
| Estimated proportion (%) of NHRs among total number of patients cared for (median [IQR], min–max)  *Missing* | 5.0 [2.9–10.0],  0.5–20.0  *1* | 5.0 [2.5–10.0],  0.5–15.0  *1* | 8.5 [5.0–10.0],  3.5–15.0  *-* | 6.0 [2.0–11.0],  0.5–15.0  *-* |
| Estimated hours per week required for medical care for NHRs (median [IQR], min–max)  *Missing* | 5.2 [3.0–7.9],  0.5–36.6  *-* | 5.0 [2.9–7.6],  0.0–15.0  *1* | 6.5 [3.5–8.0],  2.0–15.7  *-* | 5.0 [2.0–9.0],  0.0–15.0  *-* |
| Delegation of NH visits to medical assistants, yes (n [valid %])  *Missing* | 29 [67.4]  *-* | 26 [74.3]  *-* | 9 [75.0]  *-* | 6 [66.7]  *-* |

Abbreviations: *GP,* general practitioner; *IQR,* interquartile range; *max,* maximum; *min,* minimum; *NH,* nursing home; *NHR,* nursing home resident; *T2,* 12 months post randomization.

### Sample description: Registered Nurses

**Table 2.17** Characteristics of the registered nurses

|  | **Quantitative process evaluation** | | **Qualitative process evaluation** | |
| --- | --- | --- | --- | --- |
|  | Intervention group | Control group | Intervention group | Control group |
| **Registered Nurses (Baseline)** |  |  |  |  |
| Number | 72 | 72 | 28 | 25 |
| Age (median [IQR], min–max)  *Missing* | 39.0 [30.0–53.5],  23.0–61.0  *3* | 35.0 [28.5–44.5],  20.0–60.0  *7* | 48.0 [37.5–57.3],  24.0–61.0  *2* | 35.0 [28.0–45.0],  23.0–60.0  *2* |
| Sex, female (n [valid %])  *Missing* | 56 [78.9]  *1* | 57 [82.6]  *3* | 19 [70.4]  *1* | 16 [64.0]  *2* |
| Type of first cycle nursing degree, geriatric nursing care (n [valid %])  *Missing* | 57 [80.3]  *1* | 57 [82.6]  *3* | 23 [85.2]  *1* | 16 [69.6]  *2* |
| Additional nursing qualifications, yes (n [valid %])  *Missing* | 32 [44.4]  *-* | 30 [41.7]  *-* | 9 [32.1]  *-* | 10 [40.0]  *-* |
| Years of experience as registered nurse (median [IQR], min–max)  *Missing* | 14.0 [6.0–20.0],  1.0–43.0  *3* | 9.0 [4.0–14.0],  0.5–36.0  *7* | 15.0 [7.5–20.5],  1.0–40.0  *2* | 10.0 [6.0–14.0],  1.0–32.0  *2* |
| Years of experience as registered nurse in long-term care (median [IQR], min–max)  *Missing* | 12.5 [6.0–20.0],  1.0–40.0  *2* | 8.0 [4.0–11.8],  0.5–36.0  *8* | 14.0 [7.0–22.0],  1.0–40.0  *1* | 9.0 [3.5–12.0],  1.0–32.0  *4* |
| Years of experience as registered nurse in the participating NH (median [IQR], min–max)  *Missing* | 8.0 [3.0–14.3],  0.4–24.0  *2* | 3.0 [1.5–7.5],  0.3–28.0  *7* | 10.5 [7.0–19.5],  0.4–24.0  *-* | 2.0 [0.8–3.0]  0.3–17.0  *2* |
| Working hours per week (median [IQR], min–max)  *Missing* | 38.7 [35.5–40.0],  20.0–45.0  *8* | 38.5 [35.0–40.0],  19.0–40.0  *11* | 38.5 [36.8–39.0],  28.9–40.0  *7* | 38.5 [34.5–40.0],  30.0–40.0  *3* |
| **Registered Nurses (T2 measuring point)** |  |  |  |  |
| Number | 70 | 62 | 25 | 11 |
| Age (median [IQR], min–max)  *Missing* | 39.5 [31.0–50.0],  23.0–62.0  *8* | 34.0 [29.5–44.5],  21.0–68.0  *5* | 40.0 [32.5–50.3],  24.0–62.0  *3* | 37.0 [33.0–46.8],  22.0–50.0  *3* |
| Sex, female (n [valid %])  *Missing* | 51 [79.7]  *6* | 47 [77.0]  *1* | 14 [63.6]  *3* | 9 [81.8]  *-* |
| Type of first cycle nursing degree, geriatric nursing care (n [valid %])  *Missing* | 57 [87.7]  *5* | 54 [88.5]  *1* | 20 [87.0]  *2* | 10 [90.9]  *-* |
| Additional nursing qualifications, yes (n [valid %])  *Missing* | 25 [40.3]  *8* | 26 [51.0]  *11* | 9 [39.1]  *2* | 6 [54.5]  *-* |
| Years of experience as registered nurse (median [IQR], min–max)  *Missing* | 10.0 [4.5–20.0],  0.3–35  *5.0* | 9.0 [4.0–14.5],  0.1–36.0  *4* | 14.0 [5.0–20.0],  0.3–32.0  *2* | 11.0 [7.0–15.5],  0.2–26.0  *2* |
| Years of experience as registered nurse in long-term care (median [IQR], min–max)  *Missing* | 10.0 [5.0–18.4],  0.3–35.0  *6* | 9.3 [4.0–14.3],  0.5–35.0  *4* | 9.0 [5.0–19.0],  0.3–32.0  *2* | 10.5 [6.5–16.8],  3.0–20.0  *3* |
| Years of experience as registered nurse in the participating NH (median [IQR], min–max)  *Missing* | 6.0 [2.6–10.8],  0.5–27.0  *6* | 6.0 [2.0–10.0],  0.5–26.0  *3* | 10.0 [4.5–18.0],  1.0–27.0  *4* | 3.0 [2.0–7.5],  0.8–18.0  *2* |
| Working hours per week (median [IQR], min–max)  *Missing* | 39.0 [33.0–40.0],  20.0–40.0  *7* | 40.0 [38.5–40.0],  25.0–77.0  *6* | 38.5 [32.0–39.0],  28.0–39.0  *3* | 40.0 [38.5–40.0],  38.0–40.0  *2* |

Abbreviations: *IQR,* interquartile range; *max,* maximum; *min,* minimum; *NH,* nursing home; *T2,* 12 months post randomization.

### Sample description: *interprof* ACT agents (intervention group in quantitative process evaluation only)

**Table 2.18** Characteristics of *interprof* ACT agents and substitutes

|  | **Quantitative process evaluation** |
| --- | --- |
|  | Intervention group |
| ***interprof* ACT agents and substitutes (first/second training or retraining)** |  |
| Number | 45* |
| Age (median [IQR], min–max)  *Missing* | 39.0 [34.0–51.0],  25.0–65.0  *2* |
| Sex, female (n [valid %])  *Missing* | 38 [84.4]  *-* |
| Type of first cycle nursing degree, geriatric nursing care (n [valid %])  *Missing* | 28 [62.2]  *-* |
| Additional nursing qualifications, yes (n [valid %])  *Missing* | 35 [79.5]  *1* |
| Years of experience as registered nurse (median [IQR], min–max)  *Missing* | 14.0 [8.0–21.0],  0.2–40.0  *3* |
| Years of experience as registered nurse in long-term care (median [IQR], min–max)  *Missing* | 11.0 [8.0–20.0],  0.0–34.0  *3* |
| Years of experience as registered nurse in the participating NH (median [IQR], min–max)  *Missing* | 5.0 [1.1–10.0],  0.1–25.0  *1* |
| Working hours per week (median [IQR], min–max)  *Missing* | 39.0 [38.5–40.0],  30.0–45.0  *2* |

Abbreviations: *IQR,* interquartile range; *max,* maximum; *min,* minimum; *NH,* nursing home.

*The sample includes originally nominated *interprof* ACT agents (and their substitutes) and those who were nominated and trained later in the study to compensate for unexpected vacancies in the *interprof* ACT agent position, e.g., due to sickness or job leave.

## Implementation strategies and activities

### Use of implementation strategies

We developed several strategies to facilitate the implementation of the *interprof* ACT intervention package in intervention nursing homes (n=17). Details of the implementation strategies are described elsewhere [1]. Tables 2.19–2.21 provide an overview of the characteristics of the implementation strategies and data on how they were implemented and perceived by the target groups.

**Table 2.19** Designation and training of *interprof* ACT agents – characteristics and implementation

| Roles and tasks of *interprof* ACT agents and substitutes | - Provide information to the staff of the nursing home about the *interprof* ACT study and intervention package - Plan, implement and evaluate local activities for the implementation of the intervention components in the nursing home - Communicate with the study team members |
| --- | --- |
| **First training** | |
| *Aim* | To provide information to the *interprof* ACT agents in relation to the study, the objectives, components and procedures of the intervention package and their own roles and tasks |
| *Time point and duration* |  |
| Planned | Time point: Shortly after designation of *interprof* ACT  Duration: 2 hours |
| Implemented (median [IQR]) | Time point: 12 days [6–15] after randomization  Duration: 1:20 hours [1:10–1:25] |
| *Number of trainings* | n=17 (minutes are available for only 15 trainings) |
| *Number of participants per training (median [IQR])* | 2 [2–2] |
| Role of participants | - *interprof* ACT agents: n=15 - Substitutes of *interprof* ACT-agents: n=10   In addition, sometimes nursing home directors (n=6) or other registered nurses (n=1) were interested in the training and participated. |
| *Discussed topics* |  |
| Planned | - Topic 1: Background, aims, intervention components and study procedure of *interprof* ACT - Topic 2: Role of *interprof* ACT agents, procedure for obtaining the opinion picture in nursing home - Topic 3: Kick-off meeting (aims, procedure, tasks, organization) |
| Implemented | N trainings with all contents addressed as planned:   - Topic 1: n=10/15 - Topic 2: n=13/15 - Topic 3: n=11/15   (Adaptations are outlined below.) |
| *Methods* |  |
| Planned | - Presentation - Discussion |
| Implemented | N trainings without deviation from the methodological-didactical concept:   - Topic 1: n=13/15 - Topic 2: n=13/15 - Topic 3: n=11/15   (Adaptations are outlined below.) |
| *Adaptations* | Content adaptations:   - Study and the intervention package were already known (n=3) - Kick-off meeting was not explicitly discussed with *interprof* ACT agents, as moderation or organization of the kick-off meeting was discussed with the nursing home director instead (n=2)   Methodological-didactic adaptations:   - It was necessary to be motivating (n=1) - Less time was available (n=1) |
| Satisfaction with training | Participants: How satisfied were you with the training regarding the training content? (4-step Likert scale: 1=totally, 2=rather, 3=rather not, 4=not at all)   - n=26/34 rated “1” - n=8/33 rated “2”   Moderators: How satisfied were you with the training provided? (5-step Likert scale ranging from 1=highly satisfied to 5=highly dissatisfied)   - n=5/14 rated “1” - n=5/14 rated “2” - n=4/14 rated “3” - n=1 missing |
| Confidence into implementation abilities | How confident are you to implement the tasks and roles covered in the training at your workplace? (6-step Likert scale: 1=very confident, 2=mostly confident, 3=rather confident, 4=rather not confident, 5=mostly not confident, 6=not at all confident)   - n=10/33 rated “1” - n=15/33 rated “2” - n=8/33 rated “3” - n=1 missing |
| **Second training** | |
| *Aim* | First steps to pave the way for the local implementation of the *interprof* ACT intervention components as agreed-upon during the kick-off meeting |
| *Time point and duration* |  |
| Planned | Time point: 1–2 weeks after the kick-off meeting  Duration: 2 hours |
| Implemented (median [IQR]) | Time point: 12 days [8–21] after the kick-off meeting  Duration: 1:00 hours [0:50–1:05] |
| *Number of trainings* | n=17 |
| *Number of participants per training (median [IQR])* | 2 [2–2] |
| *Role of participants* | - *interprof* ACT-agents: n=14 - Substitutes of *interprof* ACT-agents: n=6   In addition, sometimes nursing home directors (n=4) or other registered nurses (n=3) were interested in the training and participated. |
| *Discussed topics* |  |
| Planned | - Topic 1: Tasks of *interprof* ACT agents - Topic 2: Implementation of *interprof* ACT intervention package - Topic 3: Handling barriers - Topic 4: Supervision by study team |
| Implemented | N trainings with all contents addressed as planned:   - Topic 1: n= 16/17 - Topic 2: n=15/17 - Topic 3: n=7/17 - Topic 4: n=15/17   (Adaptations are outlined below.) |
| *Methods* |  |
| Planned | - Presentation - Discussion |
| Implemented | N trainings without deviation from the methodological-didactical concept:   - Topic 1: n=16/17 - Topic 2: n=12/17 - Topic 3: n=12/17 - Topic 4: n=15/17   (Adaptations are outlined below.) |
| *Adaptations* | Content adaptations:   - Planned role play was not carried out when the intervention component “Shared goal setting” was not implemented (n=2) - Barriers were not discussed in detail because the *interprof* ACT agents wanted to learn the topic by studying on one’s own (n=4)   Methodological-didactic adaptations:   - Role-play was not conducted (n=2) - Less time was available (n=1) |
| *Satisfaction with training* | Participants: How satisfied were you with the training regarding the training content? (4-step Likert scale: 1=totally, 2=rather, 3=rather not, 4=not at all)   - n=30/32 rated “1” - n=1/32 rated “2” - n=1/32 rated “3”   Moderators: How satisfied were you with the training provided? (5-step Likert scale ranging from 1=highly satisfied to 5=highly dissatisfied)   - n=8/17 rated “1” - n=6/17 rated “2” - n=1/17 rated “3” - n=1/17 rated “4” - n=1/17 rated “5” |
| *Confidence into implementation abilities* | How confident are you to implement the tasks and roles covered in the training at your workplace? (6-step Likert scale: 1=very confident, 2=mostly confident, 3=rather confident, 4=rather not confident, 5=mostly not confident, 6=not at all confident)   - n=10/32 rated “1” - n=17/32 rated “2” - n=5/32 rated “3” |
| **Both trainings** | |
| *Usefulness of the trainings (T2, 12 months post randomization)* | When you take a look back and think about the trainings you received at the beginning of the study: How useful were they for you? (6-step Likert scale: 1=very useful, 2=mostly useful, 3=rather useful, 4=rather not useful, 5=mostly not useful, 6=not at all useful)   - n=6/22 rated “1” - n=12/22 rated “2” - n=3/22 rated “3” - n=1/22 rated “5” - n=2 missing |

Abbreviations: *IQR,* interquartile range.

**Table 2.20** Kick-off meetings – characteristics and implementation

| **Kick-off meeting** | |
| --- | --- |
| *Aim* | To achieve agreement between all involved parties per cluster (nursing home and general practitioner) on the *interprof* ACT intervention components to be implemented in the nursing home |
| *Time point and duration* |  |
| Planned | Time point: within 3 weeks after the first training  Duration: 2 hours |
| Implemented (median [IQR]) | Time point: 20 days [13–28] after the first training  Duration: 1:43 hours [1:33–1:52] |
| *Number of kick-off meetings* | n=17 |
| *Number of participants per kick-off meeting (median [IQR])* | 8 [5–10] |
| *Role of participants* | - Nursing home directors: n=23 - Nursing home residents: n=27 - Relatives of nursing home residents: n=19 - General practitioners: n=31 - Registered nurses: n=39 |
| *Discussed topics* |  |
| Planned | - Introduction of *interprof* ACT intervention package - Discussion of *interprof* ACT intervention package |
| Implemented | Decisions about the implementation of *interprof* ACT intervention components are described in Chapter 2.3 “Implementation of *interprof* ACT components”. |
| *Satisfaction with results of discussion* | I am satisfied with the results of the discussion. (5-step Likert scale: 1=agree completely, 2=rather agree, 3=partly agree, 4=rather disagree, 5=disagree completely)  Participants:   - n=71/105 rated “1” - n=29/105 rated “2” - n=4/105 rated “3” - n=1/105 rated “4” - n=12 missing   Moderators:   - n=16/31 rated “1” - n=13/31 rated “2” - n=2/31 rated “3” |
| *Confidence into successful implementation* | How confident are you that the implementation of the agreed intervention components will be successful? (6-step Likert scale: 1=very confident, 2=mostly confident, 3=rather confident, 4=rather not confident, 5=mostly not confident, 6=not at all confident)  Participants:   - n=31/103 rated “1” - n=53/103 rated “2” - n=16/103 rated “3” - n=3/103 rated “4” - n=14 missing   Moderators:   - n=5/31 rated “1” - n=19/31 rated “2” - n=7/31 rated “3” |

Abbreviations: *IQR,* interquartile range.

**Table 2.21** Supervision of *interprof* ACT agents – characteristics and implementation

| **Supervision of the *interprof* ACT agents by study team members** | |
| --- | --- |
| *Aim* | To reflect on the advancements and barriers occurring during the implementation of agreed-upon *interprof* ACT components and to identify potential strategies to address identified barriers |
| *Time point and duration* |  |
| Planned | Timepoint: After second training until end of study  Duration:   - First three months after kick-off meeting:   - Two to four phone/electronic contacts per month   - One to three face-to-face meetings - Remaining study period:   - One to two monthly phone/electronic contacts   - One face-to-face meeting every second month |
| Implemented (median [IQR]) | Timepoint after the second training:   - Face-to-face meetings: 56 days [49–84] - Phone/electronic contact: 21 days [13–28]   Number of supervisions:   - Face-to-face meetings per nursing home: 3 [1–3] - Phone/electronic contacts per nursing home: 20 [9–27] |
| *Role of participants* | Face-to-face meetings:   - *interprof* ACT agents: n=44 - Substitutes of *interprof* ACT agents: n=22 - Nursing home directors: n=7 - Registered nurses: n=1 - Representatives of quality management: n=2   Phone/electronic contacts:   - *interprof* ACT agent: n=167 - Substitutes of *interprof* ACT agents: n=37 - Nursing home directors: n=30 - Registered nurses: n=34 - Staff from the ward in nursing home: n=23 - Other persons: n=25 |
| *Discussed topics* | - Progress of implementation (total and for every intervention component) - Barriers and facilitators of implementation |
| *Usefulness of the supervisions (T2, 12 months post randomization)* | Over the past several months, you have been regularly supervised by a study team member in your work as *interprof* ACT agent or substitute, including phone calls and face-to-face meetings: How useful was this supervision for you? (6-step Likert scale: 1=very useful, 2=mostly useful, 3=rather useful, 4=rather not useful, 5=mostly not useful, 6=not at all useful)   - n=11/24 rated “1” - n=8/24 rated “2” - n=5/24 rated “3” |

Abbreviations: *IQR,* interquartile range.

### Implementation activities (*interprof* ACT agents)

**Fig. 2.1** Additional activities of IPAVs (T1, n=19 IPAVs from 13 NHs)

The reasons for missing questionnaires from k=4 NHs were as follows: no IPAV nominated at T1 (previous IPAV no longer employed at the NH (k=1)), IPAV had no time resources and was difficult to reach (k=1), prolonged absence due to illness (k=1), and no information (k=1).

Abbreviations: *GP,* general practitioner; *IPAV,* *interprof* ACT agent; *NH,* nursing home; *NHD,* nursing home director; *NHR,* nursing home resident; *RN,* registered nurse; *T1,* follow-up after 6 months.

### Incorporation of the *interprof* ACT components into routine care (NoMAD questionnaire)

**Fig. 2.2** Answers to the NoMAD questionnaire [3, 4]

Reasons for missing questionnaires from NHs:
IPAV T1 (k=4): no IPAV nominated at T1 (previous IPAV no longer employed at the NH) (k=1), IPAV had no time resources and was difficult to reach (k=1), prolonged absence due to illness (k=1), no information (k=1)

IPAV T2 (k=3): prolonged absence due to illness (k=1), beginning of the COVID-19 pandemic (k=1), no information (k=1)

RN T2 (k=2): no information (k=2).

Abbreviations: *IPAV,* *interprof* ACT agent; *NH,* nursing home; *NoMAD,* normalization measure development; *RN,* registered nurse; *T1,* follow-up after 6 months; *T2,* follow-up after 12 months.

## Implementation of *interprof* ACT components

### Decisions made during the kick-off meetings

For each intervention component, several predefined aspects had to be discussed during the kick-off meetings to reach a consensus about the required changes in existing local structures and procedures for successful implementation (Table 2.22). In the minutes of the kick-off meetings, the final decisions made for each of these aspects per component were recorded. Based on these data and predefined decision rules (Table 2.23), two authors independently classified the decisions on the implementation of each intervention component per nursing home into five categories: already implemented, implementation without adaptation, implementation with adaptation, implementation unclear, and no implementation (Figure 2.3). In cases of disagreement, a consensus was reached by discussion and/or by involving a third person.

**Table 2.22** Predefined aspects of intervention components to be discussed during the kick-off meetings

| **Name badges (4 aspects)** |
| --- |
| - GPs wear name badges (with function, if applicable) during home visits. - RNs wear name badges (with function, if applicable) during home visits. - Storage place for the name badges of GPs is to be defined. - GPs carries name badge with him or her or receives it from designated contact person in NH. |
| **Mandatory availability rules (8 aspects)** |
| - One RN carries the telephone with him or her at all times. - For times of nonavailability: use of an automatic answering machine, timely tapping of the phone. - Arrangements between GPs and RNs regarding availability outside of office hours, GP’s mobile phone number or special number (direct GP’s phone number in GPs’ office). - Use of an *interprof* ACT standardized fax sheet. - Respect the working hours of the GPs. - Sender is responsible for obtaining a response if this is not forthcoming (e.g., via a reminder by fax or telephone). - Designated contact person in the NH is responsible for information flow to other colleagues. - Integrate “fax culture” if necessary: RN or designated contact person send a fax with the names and reasons for standardized GPs’ home visit to GP one or two days in advance. GP confirms the date of home visit by fax and, if necessary, expresses wishes in advance or informs whether the home visit will not take place or will take place at a different time. |
| **Designated contact person (6 aspects)** |
| - For each shift during the day, an RN is the designated contact person for communication with GPs; staff stability (often the same contact person) is desirable in this case. - Tasks of contact person in NH:   - Ensures team-internal communication in the NH and external communication with the GP (telephone and faxes) and ensures availability.   - Organizes ward rounds or offers to accompany ward rounds and provides time resources for a scheduled ward round.   - Ensures that instructions are implemented. - GPs appoint a member of staff in their office as the designated contact person for the NH. - Designated contact person in GPs’ office directs and prioritizes information and requests from RNs to GPs and prepares it for submission to the GP. |
| **Standardized GPs‘ home visit (8 aspects)** |
| - GPs home visit is planned and carried out in an organized manner (standardized procedures). - Either planned on a weekly basis, announced two days in advance and with a time slot of approx. 2 hours agreed upon, or for an acute reason. - If relatives are present, they are informed about the process of the visit. - RN structures and prioritizes concerns in advance. - Discussion between GP and RN before the visit. - NHRs visit with or without RN accompaniment (RN is encouraged to accompany the GPs’ home visit of the NHR). - Final discussion or, if RN was not present during visit, follow-up discussion with GP. - GP documents clear instructions in the resident’s file. |
| **Pro re nata medication (6 aspects)** |
| - There are mandatory instructions from the GP for each NHR regarding pro re nata medication. - Description of needs for individual (expected, or not yet occurred) health problems. - Use of a standardized form “Pro re nata medication”. - Concrete and clear instructions for pro re nata medication for four given symptoms (fever, pain, nausea, constipation) documented by the GP. - Assist in the administration of pro re nata medications using a form with information on symptoms or side effects, dosage and maximum daily dose. - Regular monitoring (frequency to be defined) of pro re nata medication by GP is necessary. |
| **Shared goal setting (8 aspects)** |
| - Overall and long-term goals for medical care and NHRs-specific care and therapy goals are defined and documented by all involved parties (e.g. GP, NHR, RNs and, if applicable, relatives). - The goals and wishes of the NHR are the main focus. - NHRs receive a helpful information sheet (content: definition of shared goal setting, place for questions and important topics), other participants receive a pocket card (content: definition and aims of shared goal setting, examples of possible topics). - Use of the “shared goal setting” form. - “Shared goal setting” form is archived in the nursing documentation (copy for GP, if desired) and linked to the care planning. - Avoid double documentation, do not repeat the already existing goals from the care planning. - Three goals per meeting are aimed for. - Goals are to be evaluated once a quarter in NH, online or by telephone. |

Abbreviations: *GP,* general practitioner; *NH,* nursing home; *NHR,* nursing home resident; *RN,* registered nurse.

**Table 2.23** Decision rules for classification of the decisions on implementation of the intervention components in the nursing home of interest and associated general practitioners’ offices during the kick-off meeting

|  | | **≥50% of aspects of intervention component** | | | | |
| --- | --- | --- | --- | --- | --- | --- |
|  |  | Already implemented | Implementation without adaptation | Implementation with adaptation | Implementation unclear | No implementation |
| **<50% of aspects of intervention component** | Already implemented | Already implemented (no implementation) | Implementation without adaptation | Implementation with adaptation | No implementation | No implementation |
|  | Implementation without adaptation |  | Implementation without adaptation | Implementation with adaptation | Implementation without adaptation | Implementation without adaptation |
|  | Implementation with adaptation |  |  | Implementation with adaptation | Implementation with adaptation | Implementation with adaptation |
|  | Implementation unclear |  |  |  | Implementation unclear  (no implementation*) | No implementation |
|  | No implementation |  |  |  |  | No implementation |

*If intervention component has already been implemented to some degree.

**Fig. 2.3** Decision about implementation at the kick-off meeting

Abbreviations: *GP,* general practitioner; *NH,* nursing home.

**Table 2.24** Decision about the implementation of the *interprof* ACT components at the kick-off meeting

| **Intervention component** | **Clusters (nursing homes) where intervention component has already been implemented to some degree*** | **Decisions at the kick-off meeting** | | |
| --- | --- | --- | --- | --- |
|  |  | **Implementation without adaptations** | **Implementation with adaptations** | **No implementation** |
| 17  16  15  13  14  5  6  4  3  1  11  12  9  7  8  17  16  15  13  14  3  11  10  9  17  16  15  13  14  5  6  2  11  12  10  9  7  8  6  4  1  2  16  15  13  14  11  12  10  9  16  15  13  14  5  6  4  3  2  11  12  10  8  16  15  13  14  12  10  9  Name badges |  | 17  16  13  14  6  12  10  9  17  15  13  14  5  6  4  3  1  2  11  12  10  9  7  8  5  4  3  1  17  16  15  12  10  17  16  13  14  5  3  1  2  11  12  10  9  8  17  16  15  14  5  1  11  12  10  9  15  13  6  3  2  11  9  7  5  4  1  2  11  7  11  7  7  5  1 |  | 15  3  8  16  13  14  6  2  9  8  15  6  4  13  6  4  3  2  7  8  17  16  14  4  12  10 |
| Mandatory availability rules |  |  |  |  |
| Designated contact persons |  |  |  |  |
| Standardized GPs’ home visits |  |  |  |  |
| Pro re nata medication |  |  |  |  |
| Shared goal setting |  |  |  | 8 |

The numbers in boxes represent the IDs of the clusters (nursing homes). *Agreed judgment of the kick-off meeting participants.

### Sensitivity analysis for implementation performance

**Table 2.25** Sensitivity analysis of the quantitative findings on implementation performance

| **Quantitative findings:**  **±360° global performance indicator (mean values per cluster across rater perspectives)** | | |
| --- | --- | --- |
| **Parameter** | **IG (n=17)** | **CG (n=17)** |
| **Name badges** | | |
| Median [IQR] | 120.0  [-40.2–247.55] | 0  [-69.4–71.3] |
| Mann‒Whitney-U Test | 193.5  [Z = 1.69, p = 0.091] | |
| **Mandatory availability rules** | | |
| Median [IQR] | 83.3  [50.1–111.9] | 35.3  [-51.6–72.0] |
| Mann‒Whitney-U Test | 212.0  [Z = 2.33, p = 0.020] | |
| **Designated contact persons** | | |
| Median [IQR] | 90.0  [-45.0–135.0] | 0  [-135.0–135.0] |
| Mann‒Whitney-U Test | 173.0  [Z = 0.99, p = 0.320] | |
| **Standardized GPs’ home visits** | | |
| Median [IQR] | 13.5  [-36.8–103.5] | 2.3  [-57.4–71.4] |
| Mann‒Whitney-U Test | 155.0  [Z = 0.36, p = 0.718] | |
| **Pro re nata medication** | | |
| Median [IQR] | 72.0  [-9.0–162.6] | 72.0  [-18.0–120.0] |
| Mann‒Whitney-U Test | 157.0  [Z = 0.43, p = 0.666] | |
| **Shared goal setting** | | |
| Median [IQR] | 38.4  [3.4–142.8] | 60.0  [12.0–162.0] |
| Mann‒Whitney-U Test | 128.5  [Z = -0.55, p = 0.581] | |

Abbreviations: *CG,* control group; *GP,* general practitioner; *IG,* intervention group; *IPAV, interprof* ACT agent*; IQR,* interquartile range; *NH,* nursing home; *RN,* registered nurse.

### Changes in attitudes toward intervention components

**Table 2.26** Changes in attitudes regarding *interprof* ACT intervention components

|  | **General practitioners** | | **Registered nurses** | |
| --- | --- | --- | --- | --- |
|  | **Intervention group** | **Control group** | **Intervention group** | **Control group** |
|  | ±360° summary change measure  (median [IQR]/Certainty) | | | |
| Name badges | - | - | - | - |
| Mandatory availability rules | 0 [0–360]/82% | 0 [0–360]/65% | 0 [0–0]/76% | 0 [-360–0]/59% |
| Designated contact person | 0 [0–0]/82% | 0 [0–0]/65% | 0 [0–0]/76% | 0 [0–0]/59% |
| Standardized GPs’ home visit | 0 [-225–0]/82% | 0 [0–0]/65% | 0 [0–0]/76% | 0 [0–0]/59% |
| Pro re nata medication | - | - | - | - |
| Shared goal setting | 0 [0–0]/82% | 0 [0–0]/65% | 0 [-360–0]/76% | 0 [-360–0]/59% |

±360° summary change measure = aggregated change measure for each outcome subdomain/dimension separated for each cluster (Chapter 1.2 “Data analysis”).

Certainty = The “certainty” represents the proportion of items/subscores with sufficient data to estimate the degree of change per perspective. In cases where the ±360° global performance indicator contains uncertainties due to missing data for more than 50% of the items (i.e., not displayable changes), the ±360° global performance indicator was highlighted as “uncertain”.

Abbreviations: *GPs,* general practitioners; *IQR,* interquartile range.

## Context factors of implementation

### Relevant context factors for the six intervention components

**Table 2.27** Descriptive results of relevant context factors for the intervention component “name badges”

| **Domain** | **Subdomain** | Dimension | **≥ median of mean ±360° global performance indicator per IG cluster (Table 5)** | | < **median of mean ±360° global performance indicator per IG cluster  (Table 5)** | |
| --- | --- | --- | --- | --- | --- | --- |
|  |  |  | **median [IQR]/ valid %** | **N** | **median [IQR]/ valid %** | **N** |
| **Organizational context at meso-level** | **Nursing home characteristics** | Number of places in long-term care | 86.0 [50.3–191.0] | 8 | 93.5 [65.3–114.8] | 8 |
|  |  | Proportion [%] of NHRs with care levels 3 and higher (T0a, median per cluster) | 77.4 [70.7–85.1] | 7 | 74.0 [58.9–79.5] | 8 |
|  |  | RN-NHR ratio (T0a, median per cluster) | 0.17 [0.13–0.20] | 8 | 0.16 [0.11–0.23] | 6 |
|  |  | Total number of general practitioners involved in medical care of NHRs | 12.0 [5.0–21.0] | 7 | 12.0 [5.3–19.0] | 8 |
|  |  | Preexisting cooperation agreements with GPs (T0a) | 37.5 | 3 | 62.5 | 5 |
|  | **GP office characteristics** | Involvement in academic teaching (T0a, % per cluster) | 33.3 [22.5–75.0] | 9 | 42.9 [0.0–100.0] | 7 |
|  |  | GP: Number of NHRs cared for (T0a, median per cluster) | 18.0 [3.3–26.5] | 9 | 14.0 [9.0–18.0] | 7 |
|  | **Structures of collaboration and medical care procedures** | ±360° summary change estimate –Staff resources (RNs’, and IPAVs’ perspective) (Scale: -360° (most negative changes) … 360° (most positive changes)) | 0.0 [-180.0–270.0] | 9 | -90.0 [-180.0–135.0] | 8 |
|  |  | ±360° summary change estimate – Financial resources (GPs’ perspective) (Scale: -360° (most negative changes) … 360° (most positive changes)) | 0.0 [-180.0–360.0] | 9 | 0.0 [-270.0–360.0] | 8 |
| **Staff-related context at micro-level** | **Professional charac-teristics** | Professional experience as RN (RNs’ perspective, T0a, median per cluster) | 12.0 [8.5–15.0] | 8 | 15.0 [5.8–20.0] | 8 |
|  |  | Professional experience as GP (T0a, median per cluster) | 26.0 [23.0–28.0] | 9 | 27.5 [25.0–31.0] | 7 |

±360° Summary change estimate = Aggregated change measure for each outcome subdomain/dimension separated for each cluster (Chapter 1.2 “Data analysis”).

Abbreviations: *GP,* general practitioner; *IG*, intervention group; *IPAV,* *interprof* ACT agent; *IQR,* interquartile range; *NHR,* nursing home resident; *RN,* registered nurse; *T0a,* baseline assessment (before randomized allocation).

**Table 2.28** Descriptive results of relevant context factors for the intervention component “mandatory availability rules”

| **Domain** | **Subdomain** | Dimension | **≥ median of mean ±360° global performance indicator per IG cluster (Table 5)** | | < **median of mean ±360° global performance indicator per IG cluster  (Table 5)** | |
| --- | --- | --- | --- | --- | --- | --- |
|  |  |  | **median [IQR]/ valid %** | **N** | **median [IQR]/ valid %** | **N** |
| **Organizational context at meso-level** | **Nursing home characteristics** | Number of places in long-term care | 97.0 [63.0–180.5] | 9 | 75.0 [60.0–105.0] | 7 |
|  |  | Proportion [%] of NHRs with care levels 3 and higher (T0a, median per cluster) | 76.3 [67.8–83.9] | 8 | 73.6 [65.6–81.4] | 7 |
|  |  | RN-NHR ratio (T0a, median per cluster) | 0.18 [0.14–0.27] | 8 | 0.14 [0.10–0.19] | 6 |
|  |  | Total number of general practitioners involved in medical care of NHRs | 12.0 [5.0–21.0] | 9 | 14.0 [5.8–17.8] | 6 |
|  |  | Preexisting cooperation agreements with GPs (T0a) | 44.4 | 4 | 57.1 | 4 |
|  | **GP office characteristics** | Involvement in academic teaching (T0a, % per cluster) | 40.0 [26.7–75.0] | 9 | 33.3 [0.0–100.0] | 7 |
|  |  | GP: Number of NHRs cared for (T0a, median per cluster) | 18.0 [14.5–20.5] | 9 | 9.0 [1.0–42.0] | 7 |
|  | **Structures of collaboration and medical care procedures** | ±360° summary change estimate –Staff resources (RNs’, and IPAVs’ perspective) (Scale: -360° (most negative changes) … 360° (most positive changes)) | -180.0 [-180.0–90.0] | 9 | 0.0 [-135.0–315.0] | 8 |
|  |  | ±360° summary change estimate –Financial resources (GPs’ perspective) (Scale: -360° (most negative changes) … 360° (most positive changes)) | 360.0 [0.0–360.0] | 9 | 0.0 [-360.0–0.0] | 8 |
| **Staff-related context at micro-level** | **Professional charac-teristics** | Professional experience as RN (RNs’ perspective, T0a, median per cluster) | 15.0 [9.0–16.5] | 9 | 10.0 [8.0–16.0] | 7 |
|  |  | Professional experience as GP (T0a, median per cluster) | 26.0 [22.5–30.3] | 9 | 26.0 [25.0–30.0] | 7 |

±360° Summary change estimate = Aggregated change measure for each outcome subdomain/dimension separated for each cluster (Chapter 1.2 “Data analysis”).

Abbreviations: *GP,* general practitioner; *IG*, intervention group; *IPAV,* *interprof* ACT agent; *IQR,* interquartile range; *NHR,* nursing home resident; *RN,* registered nurse; *T0a,* baseline assessment (before randomized allocation).

**Table 2.29** Descriptive results of relevant context factors for the intervention component “designated contact persons”

| **Domain** | **Subdomain** | Dimension | **≥ median of mean ±360° global performance indicator per IG cluster (Table 5)** | | < **median of mean ±360° global performance indicator per IG cluster  (Table 5)** | |
| --- | --- | --- | --- | --- | --- | --- |
|  |  |  | **median [IQR]/ valid %** | **N** | **median [IQR]/ valid %** | **N** |
| **Organizational context at meso-level** | **Nursing home characteristics** | Number of places in long-term care | 94.5 [50.3–194.5] | 8 | 88.0 [63.8–104.8] | 8 |
|  |  | Proportion [%] of NHRs with care levels 3 and higher (T0a, median per cluster) | 70.7 [65.6–77.4] | 7 | 78.6 [73.8–83.3] | 8 |
|  |  | RN-NHR ratio (T0a, median per cluster) | 0.17 [0.14–0.20] | 8 | 0.16 [0.10–0.23] | 6 |
|  |  | Total number of general practitioners involved in medical care of NHRs | 14.5 [5.0–21.0] | 8 | 12.0 [6.0–16.0] | 7 |
|  |  | Preexisting cooperation agreements with GPs (T0a) | 50.0 | 4 | 50.0 | 4 |
|  | **GP office characteristics** | Involvement in academic teaching (T0a, % per cluster) | 33.3 [10.0–75.0] | 9 | 42.9 [33.3–100.0] | 7 |
|  |  | GP: Number of NHRs cared for (T0a, median per cluster) | 16.0 [7.3–26.5] | 9 | 15.0 [1.0–20.0] | 7 |
|  | **Structures of collaboration and medical care procedures** | ±360° summary change estimate –Staff resources (RNs’, and IPAVs’ perspective) (Scale: -360° (most negative changes) … 360° (most positive changes)) | 0.0 [-180.0–360.0] | 9 | -90.0 [-180.0–135.0] | 8 |
|  |  | ±360° summary change estimate –Financial resources (GPs’ perspective) (Scale: -360° (most negative changes) … 360° (most positive changes)) | 0.0 [0.0–360.0] | 9 | 0.0 [-360.0–270.0] | 8 |
| **Staff-related context at micro-level** | **Professional charac-teristics** | Professional experience as RN (RNs’ perspective, T0a, median per cluster) | 12.0 [8.0–15.0] | 8 | 15.0 [6.3–19.8] | 8 |
|  |  | Professional experience as GP (T0a, median per cluster) | 26.0 [23.0–30.0] | 9 | 26.0 [25.0–30.5] | 7 |

±360° Summary change estimate = Aggregated change measure for each outcome subdomain/dimension separated for each cluster (Chapter 1.2 “Data analysis”).

Abbreviations: *GP,* general practitioner; *IG*, intervention group; *IPAV,* *interprof* ACT agent; *IQR,* interquartile range; *NHR,* nursing home resident; *RN,* registered nurse; *T0a,* baseline assessment (before randomized allocation).

**Table 2.30** Descriptive results of relevant context factors for the intervention component “standardized GPs’ home visits”

| **Domain** | **Subdomain** | Dimension | **≥ median of mean ±360° global performance indicator per IG cluster (Table 5)** | | < **median of mean ±360° global performance indicator per IG cluster  (Table 5)** | |
| --- | --- | --- | --- | --- | --- | --- |
|  |  |  | **median [IQR]/ valid %** | **N** | **median [IQR]/ valid %** | **N** |
| **Organizational context at meso-level** | **Nursing home characteristics** | Number of places in long-term care | 95.0 [63.0–180.5] | 9 | 75.0 [60.0–104.0] | 7 |
|  |  | Proportion [%] of NHRs with care levels 3 and higher (T0a, median per cluster) | 74.0 [58.8–76.9] | 8 | 81.4 [70.7–85.1] | 7 |
|  |  | RN-NHR ratio (T0a, median per cluster) | 0.18 [0.13–0.29] | 7 | 0.17 [0.11–0.19] | 7 |
|  |  | Total number of general practitioners involved in medical care of NHRs | 12.0 [5.0–21.0] | 9 | 12.0 [7.3–16.3] | 6 |
|  |  | Preexisting cooperation agreements with GPs (T0a) | 66.7 | 6 | 28.6 | 2 |
|  | **GP office characteristics** | Involvement in academic teaching (T0a, % per cluster) | 42.9 [10.0–100.0] | 9 | 33.3 [25.0–66.7] | 7 |
|  |  | GP: Number of NHRs cared for (T0a, median per cluster) | 16.0 [12.0–25.0] | 9 | 9.0 [1.0–21.0] | 7 |
|  | **Structures of collaboration and medical care procedures** | ±360° summary change estimate –Staff resources (RNs’, and IPAVs’ perspective) (Scale: -360° (most negative changes) … 360° (most positive changes)) | -180.0 [-180.0–0.0] | 9 | 90.0 [-135.0–315.0] | 8 |
|  |  | ±360° summary change estimate –Financial resources (GPs’ perspective) (Scale: -360° (most negative changes) … 360° (most positive changes)) | 360.0 [-360.0–360.0] | 9 | 0.0 [0.0–0.0] | 8 |
| **Staff-related context at micro-level** | **Professional charac-teristics** | Professional experience as RN (RNs’ perspective, T0a, median per cluster) | 14.0 [6.5–16.0] | 9 | 15.0 [8.0–16.0] | 7 |
|  |  | Professional experience as GP (T0a, median per cluster) | 26.0 [24.5–30.3] | 9 | 26.0 [25.0–30.0] | 7 |

±360° Summary change estimate = Aggregated change measure for each outcome subdomain/dimension separated for each cluster (Chapter 1.2 “Data analysis”).

Abbreviations: *GP,* general practitioner; *IG*, intervention group; *IPAV,* *interprof* ACT agent; *IQR,* interquartile range; *NHR,* nursing home resident; *RN,* registered nurse; *T0a,* baseline assessment (before randomized allocation).

**Table 2.31** Descriptive results of relevant context factors for the intervention component “pro re nata medication”

| **Domain** | **Subdomain** | Dimension | **≥ median of mean ±360° global performance indicator per IG cluster (Table 5)** | | < **median of mean ±360° global performance indicator per IG cluster  (Table 5)** | |
| --- | --- | --- | --- | --- | --- | --- |
|  |  |  | **median [IQR]/ valid %** | **N** | **median [IQR]/ valid %** | **N** |
| **Organizational context at meso-level** | **Nursing home characteristics** | Number of places in long-term care | 94.5 [60.8–160.8] | 10 | 85.0 [58.5–107.5] | 6 |
|  |  | Proportion [%] of NHRs with care levels 3 and higher (T0a, median per cluster) | 74.4 [61.1–78.9] | 9 | 79.0 [71.6–87.9] | 6 |
|  |  | RN-NHR ratio (T0a, median per cluster) | 0.18 [0.16–0.27] | 8 | 0.12 [0.10–0.19] | 6 |
|  |  | Total number of general practitioners involved in medical care of NHRs | 12.0 [5.0–21.0] | 10 | 12.0 [6.5–18.0] | 5 |
|  |  | Preexisting cooperation agreements with GPs (T0a) | 50.0 | 5 | 50.0 | 3 |
|  | **GP office characteristics** | Involvement in academic teaching  (T0a, % per cluster) | 41.5 [23.8–100.0] | 10 | 33.3 [0.0–75.0] | 6 |
|  |  | GP: Number of NHRs cared for (T0a, median per cluster) | 17.0 [11.9–23.8] | 10 | 9.5 [1.0–27.5] | 6 |
|  | **Structures of collaboration and medical care procedures** | ±360° summary change estimate –Staff resources (RNs’, and IPAVs’ perspective) (Scale: -360° (most negative changes) … 360° (most positive changes)) | -180.0 [-180.0–90.0] | 10 | 0.0 [-180.0–180.0] | 7 |
|  |  | ±360° summary change estimate –Financial resources (GPs’ perspective) (Scale: -360° (most negative changes) … 360° (most positive changes)) | 360.0 [-90.0–360.0] | 10 | 0.0 [-360.0–0.0] | 7 |
| **Staff-related context at micro-level** | **Professional charac-teristics** | Professional experience as RN (RNs’ perspective, T0a, median per cluster) | 12.0 [7.3–15.5] | 10 | 15.0 [9.5–17.5] | 6 |
|  |  | Professional experience as GP (T0a, median per cluster) | 26.0 [23.3–30.1] | 10 | 26.0 [25.0–31.5] | 6 |

±360° Summary change estimate = Aggregated change measure for each outcome subdomain/dimension separated for each cluster (Chapter 1.2 “Data analysis”).

Abbreviations: *GP,* general practitioner; *IG*, intervention group; *IPAV,* *interprof* ACT agent; *IQR,* interquartile range; *NHR,* nursing home resident; *RN,* registered nurse; *T0a,* baseline assessment (before randomized allocation).

**Table 2.32** Descriptive results of relevant context factors for the intervention component “shared goal setting”

| **Domain** | **Subdomain** | Dimension | **≥ median of mean ±360° global performance indicator per IG cluster (Table 5)** | | < **median of mean ±360° global performance indicator per IG cluster  (Table 5)** | |
| --- | --- | --- | --- | --- | --- | --- |
|  |  |  | **median [IQR]/ valid %** | **N** | **median [IQR]/ valid %** | **N** |
| **Organizational context at meso-level** | **Nursing home characteristics** | Number of places in long-term care | 86.5 [52.5–130.0] | 8 | 99.5 [61.5–114.8] | 8 |
|  |  | Proportion [%] of NHRs with care levels 3 and higher (T0a, median per cluster) | 76.3 [67.8–83.0] | 8 | 73.6 [65.6–81.4] | 7 |
|  |  | RN-NHR ratio (T0a, median per cluster) | 0.18 [0.13–0.29] | 7 | 0.15 [0.11–0.19] | 7 |
|  |  | Total number of general practitioners involved in medical care of NHRs | 12.0 [5.0–21.0] | 7 | 14.0 [6.5–19.3] | 8 |
|  |  | Preexisting cooperation agreements with GPs (T0a) | 37.5 | 3 | 62.5 | 5 |
|  | **GP office characteristics** | Involvement in academic teaching (T0a, % per cluster) | 42.9 [36.7–100.0] | 9 | 25.0 [0.0–66.7] | 7 |
|  |  | GP: Number of NHRs cared for (T0a, median per cluster) | 18.0 [7.5–26.5] | 9 | 10.0 [5.5–20.0] | 7 |
|  | **Structures of collaboration and medical care procedures** | ±360° summary change estimate –Staff resources (RNs’, and IPAVs’ perspective) (Scale: -360° (most negative changes) … 360° (most positive changes)) | -180.0 [-180.0–0.0] | 9 | 90.0 [-135.0–315.0] | 8 |
|  |  | ±360° summary change estimate –Financial resources (GPs’ perspective) (Scale: -360° (most negative changes) … 360° (most positive changes)) | 0.0 [-180.0–360.0] | 9 | 0.0 [-270.0–270.0] | 8 |
| **Staff-related context at micro-level** | **Professional charac-teristics** | Professional experience as RN (RNs’ perspective, T0a, median per cluster) | 12.5 [8.5–16.5] | 8 | 14.0 [8.0–16.0] | 8 |
|  |  | Professional experience as GP (T0a, median per cluster) | 26.0 [22.5–30.3] | 9 | 26.0 [25.0–30.0] | 7 |

±360° Summary change estimate = Aggregated change measure for each outcome subdomain/dimension separated for each cluster (Chapter 1.2 “Data analysis”).

Abbreviations: *GP,* general practitioner; *IG*, intervention group; *IPAV,* *interprof* ACT agent; *IQR,* interquartile range; *NHR,* nursing home resident; *RN,* registered nurse; *T0a,* baseline assessment (before randomized allocation).

### Context factors mentioned by *interprof* ACT agents during supervision

**Table 2.33** Context factors at the meso- and micro-levels mentioned by *interprof* ACT agents during supervision

| **Context factor** | **1^st^ quarter of implementation*** | | **2^nd^ quarter of implementation*** | | **3^rd^ quarter of implementation*** | | **4^th^ quarter of implementation***  **(Fig. 2.4)** | |
| --- | --- | --- | --- | --- | --- | --- | --- | --- |
|  | **Scale: 1 (very positive/helpful for implementation) … 5 (very negative/hindering for implementation)** | | | | | | | |
|  | **median [IQR]** | n | **median [IQR]** | n | **median [IQR]** | n | **median [IQR]** | n |
| **Meso-level** | | | | | | |  | |
| **Organizational leadership and work environment** | | | | | | |  | |
| Readiness/commitment of nursing home directors for implementation | 2.0 [1.0–2.5] | 5 | 2.0 [1.0–2.5] | 13 | 2.0 [1.0–2.0] | 11 | 2.0 [1.0–2.0] | 12 |
| Collaboration with colleagues in the nursing team | 2.0 [1.5–3.0] | 5 | 2.0 [1.0–3.0] | 15 | 2.0 [1.0–2.0] | 12 | 2.0 [1.0–3.0] | 11 |
| Readiness/commitment of colleagues in the nursing team for implementation | 2.0 [1.5–3.5] | 5 | 2.0 [2.0–2.3] | 14 | 2.0 [1.0–2.0] | 12 | 2.0 [1.0–3.0] | 12 |
| **Structures of interprofessional collaboration and medical care** | | | | | | |  | |
| Cooperation with nursing home directors | 2.0 [1.0–3.0] | 5 | 2.0 [1.0–2.5] | 13 | 2.0 [1.0–2.0] | 11 | 1.0 [1.0–2.0] | 12 |
| Collaboration with the general practitioners | 2.0 [2.0–3.5] | 5 | 2.0 [2-0–2.0] | 15 | 2.0 [2.0–3.0] | 12 | 2.0 [1.3–2.0] | 12 |
| Readiness/commitment of general practitioners for implementation | 2.0 [2.0–4.0] | 5 | 2.0 [2.0–3.0] | 15 | 2.0 [2.0–3.8] | 12 | 2.0 [2.0–2.0] | 12 |
| Number of registered nurses in the nursing team | 1.5 [1.0–3.5] | 4 | 2.0 [1.0–4.0] | 14 | 2.0 [2.0–3.0] | 11 | 3.0 [2.0–3.8] | 12 |
| Stability of the nursing team | 1.0 [1.0–4.0] | 5 | 2.0 [1.8–3.0] | 14 | 2.0 [2.0–3.0] | 11 | 2.0 [2.0–3.8] | 12 |
| Time resources for *interprof* ACT-work | 2.0 [2.0–4.5] | 5 | 3.0 [2.0–4.0] | 14 | 2.0 [2.0–3.0] | 12 | 3.0 [2.0–4.0] | 12 |
| Experienced recognition from colleagues for my *interprof* ACT work | 3.0 [2.5–3.0] | 5 | 2.5 [2.0–3.0] | 14 | 2.0 [2.0–3.0] | 12 | 3.0 [2.0–3.0] | 12 |
| Experienced recognition from nursing home directors for my *interprof* ACT work | 2.0 [1.0–3.0] | 5 | 2.0 [1.0–3.0] | 11 | 2.0 [2.0–2.0] | 11 | 2.5 [1.3–3.0] | 12 |
| Experienced recognition from general practitioners for my *interprof* ACT work | 2.0 [2.0–2.8] | 4 | 2.5 [2.0–3.0] | 14 | 2.0 [2.0–3.0] | 12 | 2.5 [2.0–3.0] | 12 |
| Experienced recognition from nursing home residents/relatives for my *interprof* ACT work | 3.0 [2.0–3.0] | 5 | 2.0 [1.0–3.0] | 14 | 3.0 [2.0–3.0] | 11 | 3.0 [2.3–3.0] | 12 |
| **Micro-level** | | | | | | |  | |
| **Competencies** | | | | | | |  | |
| Medical and nursing competencies of colleagues in the nursing team | 2.0 [1.5–2.5] | 5 | 2.0 [2.0–2.3] | 14 | 2.0 [2.0–2.0] | 12 | 2.0 [2.0–3.0] | 12 |
| Medical competencies of general practitioners | 2.0 [1.5–2.5] | 5 | 2.0 [2.0–2.0] | 14 | 2.0 [1.0–2.0] | 12 | 2.0 [1.0–2.0] | 12 |
| My own professional experience and competencies | 2.0 [1.0–2.0] | 5 | 2.0 [1.0–2.0] | 15 | 2.0 [1.0–2.0] | 12 | 1.5 [1.0–2.0] | 12 |
| Previous experiences/results of my *interprof* ACT work | 2.0 [2.0–3.0] | 5 | 2.0 [1.8–2.0] | 14 | 2.0 [1.0–2.0] | 12 | 2.0 [1.0–2.0] | 12 |

1st quarter (1–3 months post randomization), 2^nd^ quarter (4–6 months post randomization), 3^rd^ quarter (7–9 months post randomization), 4^th^ quarter (10–12 months post randomization)

Abbreviations: *IQR,* interquartile range; *n,* number of supervisions per quarter.

*If more than one supervision per quarter was implemented, only the last supervision per nursing home in the quarter of interest is represented in this table.

**Fig. 2.4** Context factors at the meso- and micro-levels mentioned by *interprof* ACT agents during supervision at the end of implementation (4^th^ quarter: 10–12 months post randomization)

## Interprofessional collaboration and medical care

**Table 2.34** Changes in attitudes toward interprofessional collaboration

|  | **Nursing home director** | | **General practitioners** | | **Registered nurses** | | ***interprof* ACT agent** |
| --- | --- | --- | --- | --- | --- | --- | --- |
|  | **Intervention group** | **Control group** | **Intervention group** | **Control group** | **Intervention group** | **Control group** | **Intervention group** |
|  | ±360° summary change measure  (median [IQR]/Certainty) | | | | | | |
| Involvement of nursing home residents | - | - | - | - | - | - | - |
| Interprofessional communication | - | - | - | - | - | - | 0 [0–360]/65% |
| Contribution of involved professions | - | - | 0 [-225–225]/94% | 0 [0–225]/94% | 0 [0–225]/88% | 0 [0–0]/71% | 0 [0–0]/41% |
| Coordination of care decisions | - | - | 0 [-225–225]/82% | 225 [0–225]/59% | 0 [0–225]/76% | 0 [-225–0]/53% | - |
| General interprofessional collaboration | 0 [0–360]/88% | 360 [0–360]/88% | - | - | - | - | - |
| General quality of (medical) care for nursing home residents | - | - | - | - | - | - | - |

Abbreviation: *IQR,* interquartile range.

**References**

1. Steyer L, Kortkamp C, Müller C, Tetzlaff B, Fleischmann N, Weber CE, et al. Implementation, mechanisms of change and contextual factors of a complex intervention to improve interprofessional collaboration and the quality of medical care for nursing home residents: study protocol of the process evaluation of the interprof ACT intervention package. Trials. 2022;23:561.

2. Richter C, Berg A, Langner H, Meyer G, Köpke S, Balzer K, et al. Effect of person-centred care on antipsychotic drug use in nursing homes (EPCentCare): a cluster-randomised controlled trial. Age Ageing. 2019;48:419–425.

3. Rapley T, Girling M, Mair FS, Murray E, Treweek S, McColl E, et al. Improving the normalization of complex interventions: part 1 - development of the NoMAD instrument for assessing implementation work based on normalization process theory (NPT). BMC Med Res Methodol. 2018;18:133.

4. Finch TL, Girling M, May CR, Mair FS, Murray E, Treweek S, et al. Improving the normalization of complex interventions: part 2 - validation of the NoMAD instrument for assessing implementation work based on normalization process theory (NPT). BMC Med Res Methodol. 2018;18:135.

5. Holle D, Halek M, Mayer H, Bartholomeyczik S. Die Auswirkungen der verstehenden Diagnostik auf das Belastungserleben Pflegender im Umgang mit Menschen mit Demenz in der stationären Altenhilfe [The influence of understanding diagnostics on perceived stress of nurses caring for nursing home residents with dementia]. Pflege. 2011;24:303–316.

6. Holle D, Halek M, Mayer H, Bartholomeyczik S. Die Auswirkungen der verstehenden Diagnostik auf das Belastungserleben Pflegender im Umgang mit Menschen mit Demenz in der stationären Altenhilfe [The influence of understanding diagnostics on perceived stress of nurses caring for nursing home residents with dementia]. Pflege. 2011;24:303–316.

7. van den Bussche H, Jahncke-Latteck Ä-D, Ernst A, Tetzlaff B, Wiese B, Schramm U. Zufriedene Hausärzte und kritische Pflegende – Probleme der interprofessionellen Zusammenarbeit in der Versorgung zu Hause lebender Menschen mit Demenz [Satisfied general practitioners and critical nursing staff - problems of interprofessional cooperation in the home care of dementia patients]. Gesundheitswesen. 2013;75:328–333.

8. Center for the advancement of collaborative strategies in health. Partnership self-assessment tool - questionnaire. 2002. https://atrium.lib.uoguelph.ca/xmlui/bitstream/handle/10214/3129/Partnership_Self-Assessment_Tool-Questionnaire_complete.pdf. Accessed 30 Jan 2022.

9. Schroder C, Medves J, Paterson M, Byrnes V, Chapman C, O’Riordan A, et al. Development and pilot testing of the collaborative practice assessment tool. J Interprof Care. 2011;25:189–195.

10. Lakeit S. Nurse-Physician Collaboration. A Systematic Literature Review measuring Nurse-Physician Collaboration, and the Development and Psychometric Testing of one of these Instruments for a German Target Group. Universität zu Köln (University of Cologne)

11. Bitzer E, Dierks M, Dörning H, Schwartz F. Zufriedenheit in der Arztpraxis aus Patientenperspektive - Psychometrische Prüfung eines standardisierten Erhebungsinstrumentes. [Patient satisfaction with ambulatory care physicians - psychometric testing of a standardized questionnaire]. Z Gesundh Wiss. 1999;7:196–209.

12. Klingenberg A, Bahrs O, Szecsenyi J. [How do patients evaluate general practice? German results from the European Project on Patient Evaluation of General Practice Care (EUROPEP)]. Z Arztl Fortbild Qualitatssich. 1999;93:437–445.

13. Köhler L, Weyerer S, Schäufele M. Proxy screening tools improve the recognition of dementia in old-age homes: results of a validation study. Age Ageing. 2007;36:549–554.
